# Identification of a Crosstalk among TGR5, GLIS2, and TP53 Signaling Pathways in the Control of Undifferentiated Germ Cell Homeostasis and Chemoresistance

**DOI:** 10.1002/advs.202200626

**Published:** 2022-04-18

**Authors:** Laura Thirouard, Hélène Holota, Mélusine Monrose, Manon Garcia, Angélique de Haze, Christelle Damon‐Soubeyrand, Yoan Renaud, Jean‐Paul Saru, Alessia Perino, Kristina Schoonjans, Claude Beaudoin, David H. Volle

**Affiliations:** ^1^ INSERM U1103 Université Clermont Auvergne CNRS UMR‐6293 GReD Institute Team‐Volle Clermont‐Ferrand F‐63037 France; ^2^ INSERM U1103 Université Clermont Auvergne CNRS UMR‐6293 GReD Institute Bio‐informatic facility Clermont‐Ferrand F‐63037 France; ^3^ Laboratory of Metabolic Signaling Institute of Bioengineering School of Life Sciences Ecole Polytechnique Fédérale de Lausanne Lausanne CH‐1015 Switzerland

**Keywords:** chemodrugs, germ cells, GLIS2, male fertility, stem cell regeneration, TGR5, TP53

## Abstract

Spermatogonial stem cells regenerate and maintain spermatogenesis throughout life, making testis a good model for studying stem cell biology. The effects of chemotherapy on fertility have been well‐documented previously. This study investigates how busulfan, an alkylating agent that is often used for chemotherapeutic purposes, affects male fertility. Specifically, the role of the TGR5 pathway is investigated on spermatogonia homeostasis using in vivo, in vitro, and pharmacological methods. In vivo studies are performed using wild‐type and *Tgr5*‐deficient mouse models. The results clearly show that *Tgr5* deficiency can facilitate restoration of the spermatogonia homeostasis and allow faster resurgence of germ cell lineage after exposure to busulfan. TGR5 modulates the expression of key genes of undifferentiated spermatogonia such as Gfra1 and Fgfr2. At the molecular level, the present data highlight molecular mechanisms underlying the interactions among the TGR5, GLIS2, and TP53 pathways in spermatogonia associated with germ cell apoptosis following busulfan exposure. This study makes a significant contribution to the literature because it shows that TGR5 plays key role on undifferentiated germ cell homeostasis and that modulating the TGR5 signaling pathway could be used as a potential therapeutic tool for fertility disorders.

## Graphical abstract

The present data show that TGR5 signaling interferes with GLIS2 and TP53 signaling pathways, which control stem cell renewal and survival, explaining its impacts on germ cells following injuries as illustrated using chemodrug exposures. The study makes a significant contribution to the literature because it shows that modulating the TGR5 signaling pathway could be used as a potential therapeutic tool for fertility disorders.

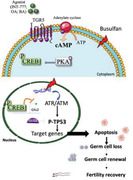

## 1 Introduction

The process of spermatogenesis is responsible for the generation of male gametes and thus, is critical for male fertility. The duration of each stage of spermatogenesis is well defined and in mouse, spermatogonia progress to spermatocytes in approximately 1–2 weeks, which then give rise to spermatids in approximately 2 weeks. Spermatogenesis relies on a balance between the self‐renewal and differentiation of germ cells to enable sperm production.^[^ ^1^, ^2^ ^]^ During adult mammalian life, the spermatogonial stem cell (SSC) pool is essential to maintain spermatogenesis.^[^ ^3^ ^]^ However, much remains to be defined regarding the regulation of SSC homeostasis. A property of SSCs is their ability to regenerate following injuries, allowing the recovery of fertility over time. Interestingly, in addition to SSC, the progenitor population of spermatogonia had been observed to participate in recovery following injury.^[^ ^4^, ^5^ ^]^ Neurogenin‐3 (Ngn3)‐positive progenitors helped replenish the pool of GFR*α*1‐positive spermatogonial cells during regeneration.^[^ ^6^ ^]^

Among all the studied models, rodents that have been acutely exposed to busulfan (Bu) is a classic model for studying spermatogenesis, as the germ cell lineage is very sensitive to chemotherapies such as alkylating agents due to their high proliferation rate.^[^ ^7^ ^]^ Thus, animal models exposed to chemodrugs are important in studying the factors involved in the regenerative capacities of undifferentiated germ cells.

Bu is a chemotherapy molecule commonly used to condition a progenitor cell transplant for the treatment of chronic myeloid leukemia. Treatment could be associated with secondary side effects. Bu treatment results in prolonged azoospermia, which impacts the germ cell lineage.^[^ ^8^ ^]^ In mice, a single dose of Bu alters spermatogenesis.^[^ ^9^ ^]^ The effect of Bu is transient and dose‐dependent; fertility could be restored, but the duration of recovery depends on the dose taken.^[^ ^7^, ^10^ ^]^ Therefore, this transient germ cell loss is useful to decipher the mechanisms involved in germ cell homeostasis associated with survival, proliferation, and/or cell differentiation processes. This is important because the deleterious impacts of chemotherapies (mainly alkylating agents) on post‐treatment quality of life, especially on fertility, is a major problem for cancer survivors. Thus, understanding the mechanisms involved in germ cell chemosensitivity is key to limit the long‐term deleterious consequences of anticancer treatments.

Bu acts preferentially by adding an alkyl group between two guanines of the DNA or between a guanine and an adenine. This leads to the formation of intra‐strand DNA bridges, resulting in single strand breaks that block DNA replication and transcription. In the longer time frame, inhibition of cell proliferation and differentiation was observed.^[^ ^9^ ^]^ DNA damages caused by Bu resulted in increased TP53 expression and activation via posttranslational modifications, such as phosphorylation, which are associated with cell apoptosis.^[^ ^11^ ^]^ This leads to increased permeability of the mitochondrial membrane, leading to the release of cytochrome C and induction of apoptosis. Furthermore, Bu‐treated testes exhibited an increase in lipid peroxidation after 1–2 weeks,^[^ ^12^, ^13^ ^]^ suggesting an increase in reactive oxygen species (ROS) production, leading to apoptosis following Bu exposure.^[^ ^14^ ^]^

Over the last decades, bile acids have been demonstrated to act as signaling molecules that regulate many physiological functions, including male fertility.^[^ ^15^, ^16^ ^]^ Bile acids act through the nuclear receptor FXR*α* (NR1H4) and the G‐protein‐coupled bile acid receptor (GPBAR‐1; TGR5).^[^ ^16^ ^]^ It was initially described that TGR5 binds to the ligand, resulting in the internalization of the receptor into the cytoplasm, leading to an increase in intracellular cyclic AMP (cAMP) concentrations and activation of the protein kinase A signaling pathway.^[^ ^17^, ^18^ ^]^ Using TGR5 knockout mice (*Tgr5^–/–^*), it was found that several metabolic pathways were under the influence of TGR5 activity, even if the *Tgr5^–/–^* mice did not show major apparent deleterious phenotypes in normal conditions.^[^ ^19^, ^20^ ^]^ TGR5 receptor appears to control bile homeostasis, energy expenditure, glucose metabolism, immunity and inflammation.^[^ ^17^, ^21^ ^]^

Within the testis, some roles of bile acids have been associated with the signaling pathways of FXR*α*.^[^ ^15^ ^]^ It controls the testicular endocrine function,^[^ ^22^, ^23^ ^]^ establishment of spermatogonial stem cells during neonatal life,^[^ ^24^, ^25^ ^]^ and adult germ cell survival.^[^ ^26^ ^]^ Parallelly, signaling pathways of bile acids through TGR5 have been defined in adult mouse testis.^[^ ^27^, ^28^ ^]^ The absence of TGR5 protects against the deleterious effects of diet‐supplemented bile acids on the testis and ultimately, on fertility.^[^ ^27^ ^]^ A diet enriched in bile acids leads to a decrease in fertility associated with testicular defects, without impacting testosterone levels. Initially, bile acids alter the expression of cell‐cell interaction genes such as Connexin‐43 and N‐Cadherin via TGR5. This leads to germ cells detaching from the seminiferous epithelium and the disruption of the blood‐testicular barrier.^[^ ^27^ ^]^

Bile acids have also been shown to have a deleterious impact on sperm quality.^[^ ^28^ ^]^ The offspring of male mice, when exposed to a bile acid‐supplemented diet, exhibited significant perinatal lethality associated with a defect in bile acid homeostasis, reduced postnatal growth, and impaired carbohydrate metabolism in adulthood.^[^ ^28^, ^29^ ^]^ These phenotypes are maintained for up to two generations of individuals and appear to be associated with lower levels of DNA methylation in the sperm of individuals that have been exposed to bile acids, compared to control animals. All these alterations are not found in *Tgr5* knockout individuals.

Together, these findings suggest major roles for TGR5 in testicular physiology and pathophysiology. TGR5 was demonstrated to be expressed in the testicular germ cell lineage in mouse and human.^[^ ^17^, ^20^, ^27^ ^]^ Consistently, analyses of published single‐cell data have shown that TGR5 expression was observed in mouse and human spermatogonia; additionally, pseudotime analyses have determined that TGR5 belongs to a group of genes corresponding to undifferentiated germ cells (supplemental data in^[^ ^30^, ^31^ ^]^). TGR5 was also found in a group of early genes along the spermatogonial trajectory, representing a pattern consistent with the SSC specification (supplemental data in^[^ ^31^ ^]^). These data suggest that within the spermatogonia, TGR5 may be involved in the transition phase from one stage to another, controlling the proliferation and/or differentiation processes. However, the roles of TGR5 in spermatogonia homeostasis have not been studied yet.

This study aimed to decipher the role of TGR5 on the germ cell response to chemotherapy using Bu and subsequent regeneration of the cells. A combination of in vivo, in vitro, and pharmacological approaches was used to define the roles of the TGR5 signaling pathway on spermatogonia homeostasis.

## 2 Results

### 2.1 Absence of TGR5 Lowers the Impacts of Bu on Male Fertility

**Figure 1:**
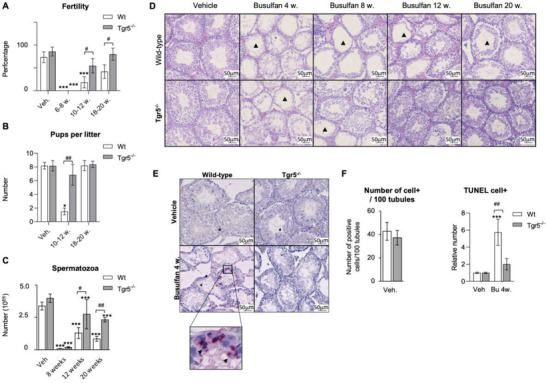
A) Percentage of fertile *Wt* or *Tgr5^–/–^* males 6–8, 10–12, or 18–20 weeks after busulfan or vehicle treatments. *n* = 10 to 15 males from 3 to 5 independent experiments. Data are expressed as the means ± SEM. ANOVA2 followed by Holm‐Sidak's test for multiple comparisons. ***, p < 0.001 versus respective vehicle group for each genotype. #, *p* < 0.05 between genotypes exposed to same treatments. The horizontal square brackets underline the groups statistically compared between two conditions of different genotypes. Veh: vehicle and Bu: Busulfan. B) Number of pups per litter obtained from fertile *Wt* or *Tgr5^–/–^* males 10–12 or 18–20 weeks after busulfan or vehicle treatments. *n* = 12–15 litters from 3 to 5 independent experiments. Data are expressed as the means ± SEM. ANOVA2 followed by Holm‐Sidak's test for multiple comparisons. *, *p* < 0.05 versus respective vehicle group for each genotype. ## *p* < 0.01 between genotypes exposed to same treatments. The horizontal square brackets underline the groups statistically compared between two conditions of different genotypes. Veh: vehicle and Bu: Busulfan. C) Number of sperm count in the epididymis head of *Wt* or *Tgr5^–/–^* males 8, 12 and 20 weeks after busulfan or vehicle treatments. *n* = 12–40 from 3 to 6 independent experiments. Data are expressed as the means ± SEM. ANOVA2 followed by Holm‐Sidak's test for multiple comparisons. ***, *p* < 0.001 versus respective vehicle group for each genotype. #, *p* < 0.05; ##; *p* < 0.01; between genotypes exposed to same treatments. The horizontal square brackets underline the groups statistically compared between two conditions of different genotypes. Veh: vehicle and Bu: Busulfan. D) Representative micrographs of hematoxylin/eosin‐stained testes of *Wt* or *Tgr5^–/‐^* males treated with the vehicle or 4, 8, 12 and 20 weeks after busulfan treatment. The arrowhead indicates tubules with germ cell loss. E) Representative micrographs of testis of vehicle or Bu treated Wt or *Tgr5^–/‐^* males stained for TUNEL. F) (Left panel) Quantification of the raw number of TUNEL positive cells for 100 seminiferous tubules of *Wt* or *Tgr5^–/–^* males treated with the vehicle treatment. (Right panel) Quantification of the relative number of TUNEL positive cells in *Wt* or *Tgr5^–/–^* males treated with the vehicle or busulfan (4 weeks after treatment). *n* = 10–24 from 3 to 6 independent experiments. Vehicle groups of each genotype were arbitrarily set at 1. Data are expressed as the means ± SEM. ANOVA2 followed by Holm‐Sidak's test for multiple comparisons. ***, *p* < 0.001 versus respective vehicle group for each genotype. ##; *p* < 0.01 between genotypes exposed to same treatments. The horizontal square brackets underline the groups statistically compared between two conditions of different genotypes. Veh: vehicle and Bu: Busulfan.

To decipher the role of TGR5 on the responsiveness of germ cells to chemotherapies, and on their subsequent regenerative capacities, 12‐week‐old wild type (*Wt*) males and *Tgr5*‐deficient (*Tgr5^–/–^*) male mice were administered a single injection of Bu (15 mg/kg).^[^ ^7^ ^]^ Exposure to Bu had almost no adverse impact on body weight in *Wt* and *Tgr5^–/–^* male mice (Figure S1A, Supporting Information). The impact of Bu on male fertility was analyzed between 6 and 20 weeks after treatment. No statistical difference between groups was found in the percentage of plugged females (Figure S1B, Supporting Information) and 6 to 8 weeks after exposure, all *Wt* and *Tgr5^–/–^* males exposed to Bu were sterile (0 out of 10 males in both genotypes) (**Figure** **1**A). Interestingly, 12 weeks after Bu exposure, only 18% (2 out of 11) of *Wt* males recovered fertility, while 55% (6 of 11) of *Tgr5^–/–^* males produced offspring (Figure 1A). After 20 weeks, 80% (8 of 10) of the *Tgr5^–/–^* busulfan‐treated males and 42% (5 of 12) of *Wt* males regained fertility (Figure 1A). In addition, after 12 weeks, treatment with Bu resulted in a drastic decrease in the number of pups per litter in *Wt* (1.5 pups per litter); whereas almost no impact was observed on *Tgr5^–/–^* males (6.8 pups per litter) compared to their respective vehicle‐treated controls (Figure 1B). These data indicate that *Tgr5^–/–^* males recovered their fertility faster than *Wt* males after exposure to the chemical drug.

These results on reproductive capacities were supported by analyzing the number of sperm cells produced; the sperm cell count in the head of the epididymis was used for this purpose. As expected in *Wt* males, Bu treatment was associated with a significant and progressive decrease in sperm cell production from 2 weeks, with a major effect at 8 weeks after treatment (Figure S1C, Supporting Information; Figure 1C, Supporting Information). Thereafter, sperm cell production increased and gradually recovered at 12 and 20 weeks after treatment with Bu (Figure 1C, Supporting Information). Consistent with reproductive capacity, no statistical difference was noticed between *Wt* and *Tgr5^–/–^* males at 8 weeks following Bu exposure; however, at 12 and 20 weeks the sperm cell counts were higher in *Tgr5^–/–^* males compared to that in *Wt* males (Figure 1C).

To better understand the impacts of Bu on sperm production in *Wt* and *Tgr5^–/–^* males, we focused on the testis. The H&E staining results showed no major impacts on testicular histology at 1 and 2 weeks (Figure S1D, Supporting Information). In contrast, *Wt* and *Tgr5^–/–^* males showed significant loss of germ cells, as shown by the thickness of the epithelium at 4 and 8 weeks (Figure 1D, Supporting Information); this was consistent with the pattern of alterations of fertility and sperm counts. No significant difference was observed in the number of apoptotic cells between genotypes under control conditions (Figure 1E,F, left panel); the significant germ cell loss, mainly observed 4 weeks after treatment, was correlated with an increased rate of apoptotic germ cells, which was greater in *Wt* males than in *Tgr5^–/–^* males (Figure 1F right panel). This lower apoptotic level in *Tgr5^–/–^* testis was followed by a more rapid regeneration phase of the germ cell lineage from 8 to 20 weeks after treatment compared to *Wt* (Figure 1D). These data indicate a primary role for TGR5 as a modulator of male reproductive capacity after injury.

### 2.2 TGR5 is a Master Gene for Undifferentiated Spermatogonia Homeostasis in Response to Bu

**Figure 2:**
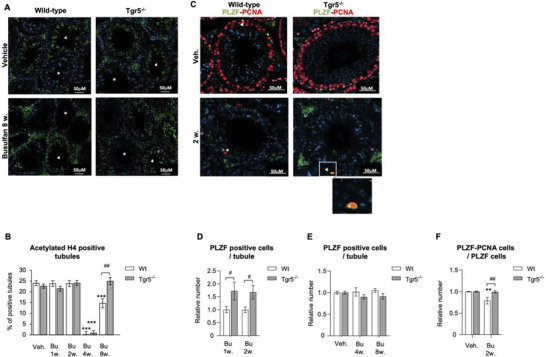
A) Representative micrographs of vehicle or Bu treated Wt or *Tgr5^–/‐^* testis stained for acetylated histone H4 (acetylated H4). B) Quantification of the number of acetylated H4 positive seminiferous tubules in testis of *Wt* or *Tgr5^–/‐^* males treated with the vehicle or busulfan (1, 2, 4, and 8 weeks after treatment). The arrowhead indicates acetylated H4 positive seminiferous tubules, and the star indicates acetylated H4 negative tubules. C) Representative micrographs of vehicle or Bu treated Wt or *Tgr5^–/‐^* testis stained for PLZF green) and PCNA (red). The arrowhead indicates PLZF‐PCNA positive cells, and the star indicates PCNA negative and PLZF positive cells. D) Quantification of the relative number of PLZF positive cells per seminiferous tubule in *Wt* or *Tgr5^–/–^* testis treated with busulfan (1 week and 2 weeks after treatment). The mean number of PLZF+ cells observed in *Wt* males treated with Bu was arbitrarily set at 1 for comparison with the number of PLZF+ cells observed one and two weeks after treatment. E) Quantification of the relative number of PLZF positive cells per seminiferous tubule in *Wt* or *Tgr5^–/–^* males treated with the vehicle or busulfan (4 weeks or 8 weeks after treatment). Vehicle groups of each genotype were arbitrarily set at 1. F) Quantification of the relative number of double stained PLZF‐PCNA cells per seminiferous tubule in *Wt* or *Tgr5^–/‐^* males treated with the vehicle or busulfan (2 weeks after treatment). Vehicle groups of each genotype were arbitrarily set at 1. In all panels, *n* = 11–23 from 3 to 6 independent experiments. Data are expressed as the means ± SEM. ANOVA2 followed by Holm‐Sidak's test for multiple comparisons. **, *p* < 0.01; ***, *p* < 0.001 versus respective vehicle group for each genotype. #, *p* < 0.05; ##, *p* < 0.01 between genotypes exposed to same treatments. The horizontal square brackets underline the groups statistically compared between two conditions of different genotypes. Veh: vehicle and Bu: Busulfan.

Analysis of spermatogenesis using specific markers showed that the number of seminiferous tubules with post‐meiotic spermatid cells (acetylated histone H4 positive cells; acetylated‐H4+) was significantly reduced 4 weeks after treatment with Bu in both the *Wt* and *Tgr5^–/–^* males (**Figure** **2**A,B). *Tgr5^–/–^* males showed faster recovery of acetylated‐H4+ spermatids, as was observed 8 weeks after exposure (Figure 2B). This pattern of recovery was consistent with the differential impact of Bu on sperm cell production and fertility between *Wt* and *Tgr5^–/–^* males.

Spermatogonia support spermatogenesis; therefore, any alteration of spermatogonia might define the capacities of fertility recovery following injury. Thus, we next analyzed the undifferentiated spermatogonia population, revealed by the PLZF (PromyeLocytic Zinc Finger; ZBTB16) marker.^[^ ^24^, ^25^ ^]^ No difference was observed in the raw number of PLZF+ cells per seminiferous tubule between the *Wt* and *Tgr5^–/‐^* males in vehicle treated groups (Figure S2A, Supporting Information). The present data showed that the undifferentiated germ cell population (PLZF+) was the first to be affected after exposure to Bu, with initial loss beginning 1 day after treatment (Figure S2A, Supporting Information). The effect of Bu on PLZF+ cell loss was similar between *Wt* and *Tgr5^–/‐^* males at 5 days after treatment with Bu, where almost 80% of these cells were lost (Figure S2A, Supporting Information). The present data showed that the relative number of PLZF+ cells was higher in *Tgr5^–/–^* male mice than in *Wt* 1 and 2 weeks after treatment with Bu (Figure 2C,D). At 4 weeks after Bu exposure, the relative number of PLZF+ cells regained normal levels and was comparable to vehicle‐treated groups in both genotypes (Figure 2E). These data suggest that the absence of *Tgr5* modulates the regenerative capacities of the PLZF+ cells, allowing an earlier emergence of germ cells. This difference was in part associated with a lower rate of proliferating spermatogonial cells in *Wt* mice where only 79% of PLZF+ cells were in proliferation (PCNA+), whereas no impact of Bu was observed in *Tgr5^–/–^* mice (Figure 2F). This may partly explain how *Tgr5^–/–^* males recovered faster than *Wt* males.

These data support the findings that Bu first affected spermatogonia, resulting in germ cell loss, which was then followed by the regeneration of the germ cell lineage, initiated from the remaining undifferentiated spermatogonia, to give rise to a new wave of spermatogenesis. In addition, these in vivo data suggests that Bu mediated its effects via alterations of the apoptotic and proliferation processes, all of which were minimized in *Tgr5^–/–^* males compared to *Wt* mice.

### 2.3 The TGR5 Signaling Pathway in Spermatogonia Plays a Major Role in the Effects of Bu Exposure

**Figure 3:**
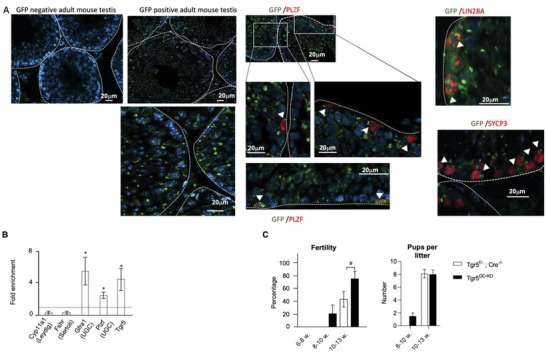
A) (Left panel) Representative micrographs of GFP immuno‐staining in GFP‐negative mouse and in hTGR5‐T2A‐GFP mouse model. (Right panel) Representative micrographs of hTGR5‐T2A‐GFP mouse model testis stained for GFP (green) and PLZF (red), or LIN28 (red) or SYCP3 (red). The white arrowheads indicate co‐stained cells. Experiment was performed on 4 different hTGR5‐T2A‐GFP males. The dotted lines delineate the seminiferous tubules. B) *Cyp11a1*, *Fshr*, *Gfra1*, *Plzf*, and *Tgr5* mRNA accumulations normalized to *β*‐actin in THY1+ cells isolated from 6 to 7 days old males. *n* = 5. The THY1‐ unsorted cell group was arbitrary set at 1 (Dotted line). Data are expressed as the means ± SEM. *T*‐test statistical analysis; *, *p* < 0.05. C) (Left panel) Percentage of fertile *Tgr5^f/‐^* ^;^ *Cre^–/‐^*, and *Tgr5^GC‐KO^* males 6–8, 8–10, and 10–13 weeks after vehicle or busulfan treatment following breeding with C57Bl6J females for 10 days. (Right panel) Number of pups per litter obtained in breeding with C57Bl6J females with *Tgr5^f/‐^* ^;^ *Cre^–/^*,*^‐^* or *Tgr5^GC‐KO^* males 8–10 and 10–13 weeks after busulfan treatment. *n* = 12–20 from 3 independent experiments. Data are expressed as the means ± SEM. ANOVA2 followed by Holm‐Sidak's test for multiple comparisons. #, *p* < 0.05; ##; *p* < 0.01; ###, *p* < 0.001 between genotypes exposed to same treatments. The horizontal square brackets underline the groups statistically compared between two conditions of different genotypes. Veh: vehicle and Bu: Busulfan.

As mentioned in the introduction, previous published data demonstrated that *Tgr5* is expressed in the germ cell lineage;^[^ ^27^ ^]^ additionally, data from recent single cell analyses showed that *TGR5* mRNA expression was observed in mouse and human spermatogonia (supplemental data in^[^ ^30^, ^31^ ^]^). This is consistent with the fact that TGR5 must play a direct role within spermatogonia, which explains why *Tgr5^–/–^* mice recovered spermatogenesis faster than *Wt* males after chemotherapeutic exposure. Next, we wanted to better decipher the roles of TGR5 within the germ cell lineage. However, none of the antibodies in our possession raised against mouse TGR5; therefore, we analyzed the expression of TGR5 by detecting GFP in a mouse TGR5‐T2A‐GFP model expressing the transgene under the control of endogenous mouse *Tgr5* promoter.^[^ ^32^ ^]^ The present results showed that the sequence driving the expression of *Tgr5* were connected to the detection of the protein in all germ cell lineage (**Figure** **3**A). Immunostaining was observed in cells near the basal membrane, mainly in spermatogonia and spermatocytes as revealed by the co‐staining of GFP with PLZF (spermatogonia), LIN28 (spermatogonia), or SYCP3 (spermatocytes) (Figure 3A). This was consistent with previous published mRNA data.^[^ ^27^ ^]^ In addition, samples from purified spermatogonia cells using Magentic cell sorting with THY1 as marker confirmed that *Tgr5* was expressed in the THY1+ spermatogonial population (Figure 3B).

Thus, to explore the roles of TGR5 within the germ cell lineage, we generated mice with a specific deletion of the *Tgr5* gene in germ cells, using *Tgr5*‐floxed mice and a model controlling recombinase‐CRE expression in germ cells through the Vasa promoter.^[^ ^33^ ^]^ As the use of recombinase‐CRE in germ cells led to the recombined allele being transmitted to the next generation, the mice used in this work were *Tgr5^f/–^* and the phenotypes of *Tgr5^f/‐^* were analyzed by comparing the response to Bu in *Cre^–/–^* and *Cre* ^+/‐^ male mice. The *Tgr5^f/–^*; *Cre* ^+/‐^ (referred to as *Tgr5^GC‐KO^*) corresponded to germ cell‐specific knockout males (Figure S2B, Supporting Information). Analysis of fertility revealed no difference among genotypes (*Tgr5^f/–^*; *Cre* ^–/‐^ and *Tgr5^GC‐KO^)* in control DMSO‐treated condition (vehicle: veh.) (Figure S2C, Supporting Information). Bu led to a complete sterility in both genotypes, with all males being sterile (0%, 0 of 10) 8 weeks after treatment (Figure 3C, left panel). Interestingly, *Tgr5^GC‐KO^* males showed an early recovery of fertility after Bu exposure compared to *Tgr5^f/–^; Cre^–/–^* males (Figure 3C, left panel). This confirmed the interaction of the Bu and TGR5 signaling pathways in the germline. Indeed, even if the difference was not significant, 20% (2 of 10) of *Tgr5^GC‐KO^* male mice recovered fertility at 10 weeks whereas all *Tgr5^f/–^; Cre^–/–^* males remained sterile (0%; 0 of 10) (Figure 3C, left panel). The fertility capacities were statistically different between genotypes 13 weeks after Bu exposure, as 73% (11 of 15) of *Tgr5^GC‐KO^* males produced offspring while only 42% (8 of 19) of *Tgr5^f/–^; Cre^–/–^* males did (Figure 3C, left panel). At 8 weeks, no progenies were obtained from *Tgr5^f/–^*; *Cre* ^–/–^ males, whereas *Tgr5^GC‐KO^* males give rise to 1.5 pups per litter, highlighting the start of fertility recovery (Figure 3C, right panel). 13 weeks after Bu exposure, the number of pups per litter was similar between groups (Figure 3C, right panel). These data support the conclusion that TGR5 acts within germ cells to modulate the effects of Bu.

### 2.4 TGR5 Controls Undifferentiated Germ Cell Homeostasis

**Figure 4:**
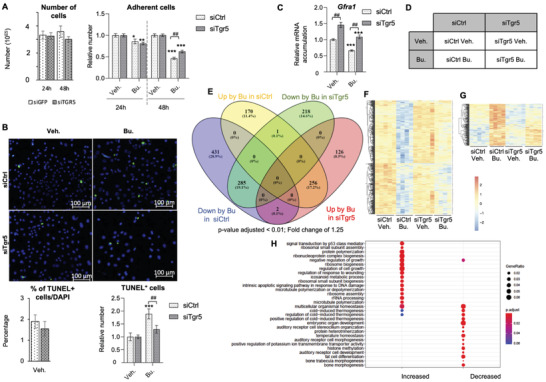
A) (Left panel) Raw number of adherent cells after 24 or 48 h of treatment with vehicle in siCtrl and siTgr5 GC1spg transfected cells; and (Right panel) relative number of adherent cells after 24 or 48 h of treatment with vehicle or busulfan in siCtrl and siTgr5 GC1spg transfected cells. Vehicle groups of each genotype were arbitrarily set at 1. In panel, *n* = 20–30 from 6 independent experiments. Data are expressed as the means ± SEM. ANOVA2 followed by Holm‐Sidak's test for multiple comparisons. *, *p* < 0.05; **, *p* < 0.01; ***, *p* < 0.001 versus respective vehicle group for each genotype. ##, *p* < 0.01 between genotypes exposed to same treatments. The horizontal square brackets underline the groups statistically compared between two conditions of different genotypes. Veh: vehicle and Bu: Busulfan. B) (Upper panel) Representative micrographs of GC1spg cells transfected with siCtrl or siTgr5 and treated with vehicle or Bu for 24 h and stained for TUNEL. (Lower left panel) Quantification of the raw number of TUNEL positive GC1spg cells transfected with siCtrl and siTgr5 and treated with vehicle. (Lower right panel) Quantification of the relative number of TUNEL positive GC1spg cells transfected with siCtrl and siTgr5 and treated with vehicle or Bu for 24 h. *n* = 5–15 from 3 to 5 independent experiments. Vehicle groups of each genotype were arbitrarily set at 1. Data are expressed as the means ± SEM. ANOVA2 followed by Holm‐Sidak's test for multiple comparisons. ***, *p* < 0.001 versus respective vehicle group for each genotype. ##, *p* < 0.01 between genotypes exposed to same treatments. The horizontal square brackets underline the groups statistically compared between two conditions of different genotypes. Veh: vehicle and Bu: Busulfan. C) Relative Gfra1 mRNA accumulation normalized to *β*‐actin on GC1spg cells transfected with siCtrl or siTgr5 and treated 24 h with vehicle or with 200 × 10^−6^ m of Bu. In panel, *n* = 25–27 from 6 independent experiments. Data are expressed as the means ± SEM. ANOVA2 followed by Holm‐Sidak's test for multiple comparisons. ***, *p* < 0.001 versus respective vehicle group for each genotype. ##, *p* < 0.01between genotypes exposed to same treatments. The horizontal square brackets underline the groups statistically compared between two conditions of different genotypes. Veh: vehicle and Bu: Busulfan. D) Representation of the RNAseq groups: GC1spg cells transfected with siCtrl or siRNA directed against Tgr5 (siTgr5) and treated 24 h with vehicle or with 200 × 10^−6^ m of Bu. E) Venn diagram for differentially expressed genes in GC1spg cells transfected with siCtrl or siTgr5 and treated 24 h with vehicle or with 200 × 10^−6^ m of Bu. F) Heatmap of differentially downregulated genes following 24 h exposure to Bu specifically in GC1spg cells transfected with siCtrl based on Table S1c1 in the Supporting Information. G) Heatmap of differentially increased genes following 24 h exposure to Bu specifically in GC1spg cells transfected with siCtrl based on Table S1c2 in the Supporting Information. H) Gene ontology analysis based on the list of differentially expressed genes specifically in GC1spg cells transfected with siCtrl following Bu exposure compared to siTgr5‐transfected cells treated with Bu. Based on gene lists Table S1c1,c2 in the Supporting Information. In panel D to H, RNAseq was performed from one experiment with *n* = 5 per group. Statistical analyses were performed as mentioned in Materials and Methods section. P value was set to *p* < 0.01 between genotypes and/or treatment.

Treatment with Bu led to a significant modulation of testicular cellularity with germ cell loss; therefore, molecular analyses on the whole testis may not be totally reliable. To better define how TGR5 modulates the effects of Bu in germ cells, we used the spermatogonial cell line GC1spg. The effects of Bu were analyzed on GC1spg cells transfected with a control siRNA (siCtrl) or an siRNA directed against *Tgr5* (siTgr5) (Figure S3A, Supporting Information). The siRNA directed against *Tgr5* has been previously used.^[^ ^27^ ^]^ As no reliable antibody raised against mouse TGR5 could be used, the impact of the knock‐down of *Tgr5* was confirmed by analyzing the expression of known TGR5 target genes that have been defined in different studies, such as *Dnmt3b* ^[^ ^28^ ^]^ or *Pcg1a* ^[^ ^34^ ^]^ (Figure S3A, Supporting Information). Analyses of transfected cells showed that in the control condition (vehicle: veh.), no difference was observed in the raw number of cells between in siCtrl or siTgr5‐transfected cells (**Figure** **4**A, left panel). To determine the respective sensitivity of siCtrl or siTgr5‐transfected cells to Bu, the vehicle‐treated cells were set to 1. Data showed that Bu exposure resulted in a similar decrease in the number of cells in siCtrl‐GC1spg and siTgr5‐GC1spg groups (around 14 to 19% respectively) 24 h after treatment with Bu (Figure 4A, right panel). The impact of Bu was then exacerbated after 48 h in siCtrl‐GC1spg cells with a 54% decrease in cell numbers (Figure 4A, right panel); however, in cells transfected with siTgr5, a lower impact was observed (Figure 4A, right panel). These data were consistent with in vivo results and suggested that lower levels of *Tgr5* attenuate the long‐term deleterious effects of Bu on spermatogonial cells.

No difference was observed in the germ cell apoptotic rate between genotypes in vehicle treated condition (Figure 4B, left panel). However, consistent with in vivo data, the decrease in the number of siCtrl‐GC1spg cells after Bu treatment was associated with a higher number of apoptotic cells compared to cells treated with vehicle (Figure 4B, right panel). This increase in Bu‐induced apoptosis was less pronounced in siTgr5‐transfected cells (Figure 4B, right panel).

The *Gfra1* gene encodes the GDNF receptor‐alpha1, which is related to undifferentiated spermatogonia self‐renewal and survival. Interestingly, mRNA accumulation of *Gfra1* was upregulated in the siTgr5‐transfected cells compared to siCtrl‐transfected cells (Figure 4C). Moreover, *Gfra1* was identified among the genes that were differentially modulated by Bu in siCtrl‐transfected cells. As confirmed by qPCR approaches, *Gfra1* expression was decreased upon Bu treatment in siCtrl‐transfected cells and the effect was less pronounced in siTgr5‐transfected cells (Figure 4C).

To better define the molecular mechanisms associated with the impacts of TGR5 signaling on Bu sensitivity, we then progressed using RNAseq approach. Four groups were analyzed (Figure 4D) corresponding to GC1spg cells transfected with siCtrl or siTgr5 and exposed to the vehicle or Bu (200 µM) for 24 h.

To better understand the functions of TGR5 in spermatogonia in the context of Bu exposure, we generated lists of genes that were differentially expressed after Bu exposure in cells transfected with siCtrl (siCtrl‐Bu vs siCtrl‐DMSO; Table S1A, Supporting Information) or with siTgr5 (siTgr5‐Bu vs siTgr5‐DMSO; Table S1B, Supporting Information).

In cells transfected with siCtrl, 1145 genes were significantly modulated by Bu (Table S1A, Supporting Information). A majority of the 718 genes (62.7%) were suppressed (Table S1A‐1, Supporting Information), while 427 (37.3%) were upregulated (Table S1A‐2, Supporting Information) compared to the vehicle‐treated group (Figure 4E). Applying the same criteria, analyses performed on cells transfected by siTgr5 showed that 888 genes were significantly modulated by Bu (Table S1B, Supporting Information). Of these, 504 (56.76%) genes were suppressed (Table S1B‐1, Supporting Information), while 384 (43.16%) genes were upregulated compared to the group treated with vehicle (Table S1B‐2, Supporting Information). To better understand how the low levels of TGR5 modulated the long‐term impacts of Bu, these lists were compared to differentiate the 601 genes that were specifically affected by Bu in cells transfected by siCtrl (Table S1C, Supporting Information). Of these, 431 were downregulated and 170 were upregulated (Table S1C1,C2, Supporting Information, respectively) (Figure 4E–G).

The lists of genes affected by Bu specifically in siCtrl‐transfected cells (Table S1C1,C2, Supporting Information) were subjected to an overrepresentation analysis of gene ontology (GO) terms according to a classification by molecular and biological process using MouseMine. The analyses revealed multiple processes such as apoptosis/DNA damage, TP53 transduction signal, or cell fate differentiation (Figure 4H). GeneMania® analysis showed the interconnections of genes that were regulated by Bu, specifically in cells transfected by siCtrl (Figure S3B, Supporting Information).

### 2.5 TGR5 Acts as a Major Regulator of the Effect of Bu on the TP53 Signal Pathway

**Figure 5:**
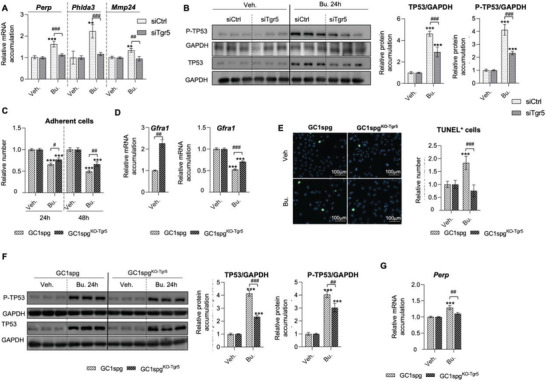
A) *Perp*, *Phlda3*, and *Mmp24* mRNA accumulations normalized to *β*‐actin on GC1spg cells transfected with siCtrl or siTgr5 and treated 24 h with vehicle or with 200 × 10^−6^ m of Bu. Vehicle groups of each genotype were arbitrarily set at 1. B) Representative western blots of GAPDH, TP53 and Phospho‐TP53 (P‐TP53), and quantification of ratios in GC1spg cells transfected with siCtrl and siTgr5 and treated with vehicle or Bu for 24 h. Vehicle groups of each genotype were arbitrarily set at 1. C) Number of adherent GC1spg and GC1spg^KO‐Tgr5^ cells exposed to veh. or Bu 200µM for 24 or 48 h. Vehicle groups of each genotype were arbitrarily set at 1. D) (Left) *Gfra1* mRNA accumulation normalized to *β*‐actin in GC1spg and GC1spg^KO‐Tgr5^ cells; GC1spg were arbitrarily set at 1. (Right) *Gfra1* mRNA accumulation normalized to *β*‐actin in GC1spg and GC1spg^KO‐Tgr5^ cells exposed to vehicle or Bu 200µM for 24 h. Vehicle groups of each genotype were arbitrarily set at 1. E) (Left) Representative micrographs of GC1spg and GC1spg^KO‐Tgr5^cells exposed to vehicle or Bu 200µM for 24 h and stained for TUNEL. (Right) Quantification of the relative number of TUNEL positive GC1spg cells and GC1spg^KO‐Tgr5^ cells exposed to vehicle or Bu 200µM for 24 h. Vehicle groups of each genotype were arbitrarily set at 1. F) Representative western blots of GAPDH, TP53 and Phospho‐TP53 (P‐TP53), and quantification of ratios in GC1spg cells and GC1spg^KO‐Tgr5^ cells treated with vehicle or Bu for 24 h. Vehicle groups of each genotype were arbitrarily set at 1. G) *Perp* mRNA accumulation normalized to *β*‐actin in GC1spg cells and GC1spg^KO‐Tgr5^ cells treated with vehicle or with 200 × 10^−6^ m of Bu for 24 h. Vehicle groups of each genotype were arbitrarily set at 1. In all panels, *n* = 6–18 from 3 to 6 independent experiments. Data are expressed as the means ± SEM. ANOVA2 followed by Holm‐Sidak's test for multiple comparisons. **, *p* < 0.01; ***, *p* < 0.001 versus respective vehicle group for each genotype. #, *p* < 0.05; ##, *p* < 0.01; ###, *p* < 0.001 between genotypes exposed to same treatments. The horizontal square brackets underline the groups statistically compared between two conditions of different genotypes. Veh: vehicle and Bu: Busulfan.

Analysis using the CisTarget software revealed that 62% (106/170) of the genes positively regulated by Bu in siCtrl‐transfected cells, with an effect that was counteracted by siTgr5 transfection (Table S2, Supporting Information), were associated with the TP53 transcription factor as supported by the defined DNA binding site (Figure S3C, Supporting Information). This was consistent with the known role of TP53 on the apoptotic process, even after Bu treatment.^[^ ^14^ ^]^ Present data showed that the impacts of Bu on apoptosis in vivo and in vitro were moderated in the context of *Tgr5* loss (*Tgr5^–/–^* mice and siTgr5 transfected cells). Among the genes identified in the TP53 network, several genes such as *Perp*, *Phlda3*, and *Mmp24*, have been validated using qPCR (**Figure** **5**A). These data highlight the identification of the crosstalk between the TGR5 and TP53 signaling pathways, as for all these genes the effect of Bu was reduced in siTgr5‐transfected cells (Figure 5A). In agreement with the above data, the western blot analyses showed that protein levels of Phospho‐TP53 (P‐TP53) and TP53 were increased in response to Bu treatment (24 h) in siCtrl‐transfected cells. These effects were less pronounced in siTgr5‐transfected cells (Figure 5B). Current data reveal how TGR5 is involved in the control of germ cell apoptosis in response to chemotherapies and highlight links between the TGR5 and TP53 signaling pathways.

To fully validate the data obtained using the siRNA‐based approach, we generated a GC1spg cell line deficient for the *Tgr5* gene (GC1spg^KO‐Tgr5^ cells), using the Crispr/Cas9 technology (see the Experimental Section and Figure S4A–E, Supporting Information). qPCR analyses showed no expression of *Tgr5* in the knock‐out cells (Figure S4D, Supporting Information). As no reliable antibody raised against mouse TGR5 could be used, the efficiency of the *Tgr5* knock‐out was confirmed using TGR5 agonists, namely INT‐777^[^ ^34^ ^]^ and oleanolic acid (OA),^[^ ^35^ ^]^ in combination with the transfection of a cAMP‐responsive element fused to luciferase (Figure S4F, Supporting Information). No induction of the luciferase activity was noticed following treatment with INT‐777 (25 or 12.5 µM) or OA in GC1spg^KO‐Tgr5^ cells (Figure S4F, Supporting Information). In contrast, when the GC1spg^KO‐Tgr5^ cells were co‐transfected with a plasmid encoding the mouse TGR5, the agonists induced luciferase activity (Figure S4F, Supporting Information).

No difference in the number of adherent cells was observed between GC1spg and GC1spg^KO‐Tgr5^ cells under control conditions (Figure S4G, Supporting Information). Bu treatment resulted in a decrease of the number of adherent cells after 24 h; the impact of Bu was exacerbated in GC1‐spg cells after 48 h with a 50% decrease in the relative cell numbers. These effects were less pronounced in the GC1spg^KO‐Tgr5^ at 24 and 48 h after Bu exposure (Figure 5C). These data are fully consistent with what was observed using the siRNA approach.

Interestingly, the data confirmed that the accumulation of *Gfra1* mRNA was increased in GC1spg^KO‐Tgr5^ compared to GC1spg (Figure 5D, left panel). In addition, the decreased expression of *Gfra1* in response to Bu was less pronounced in GC1spg^KO‐Tgr5^ cells compared to GC1spg (Figure 5D, right panel).

Consistent with the data obtained using the siRNA based‐approach, the increase in the rate of Bu‐induced apoptosis observed in GC1‐spg cells was counteracted in GC1spg^KO‐Tgr5^ cells (Figure 5E). Consistently, the impacts of Bu on P‐TP53 and TP53 levels were less pronounced in GC1spg^KO‐Tgr5^ cells compared to GC1‐spg cells (Figure 5F). It should be noted that this impact of Bu on P‐TP53 was not secondary to the induction of the apoptotic process, because there was no differential impact on the rate of apoptosis between GC1spg and GC1spg^KO‐Tgr5^ cells 6 h after treatment with Bu (Figure S5A, Supporting Information), while the effect of Bu on P‐TP53 was already observed in the siCtrl transfected cells and Gc1spg cells (Figure S5,C, Supporting Information). At 6 h after treatment, the impact on P‐TP53 was lowered in siTgr5 and GC1spg^KO‐Tgr5^ cells (Figure S5,C, Supporting Information). In addition, mRNA accumulation of the TP53 target gene, namely *Perp* was less modulated by Bu in GC1spg^KO‐Tgr5^ cells than in GC1‐spg cells (Figure 5G). These data using GC1spg^KO‐Tgr5^ cells confirmed the primary role of TGR5 to counteract the impact of Bu within germ cells.

### 2.6 TGR5‐GLIS2 Pathway Acts as a Major Regulator of Spermatogonia Homeostasis

**Figure 6:**
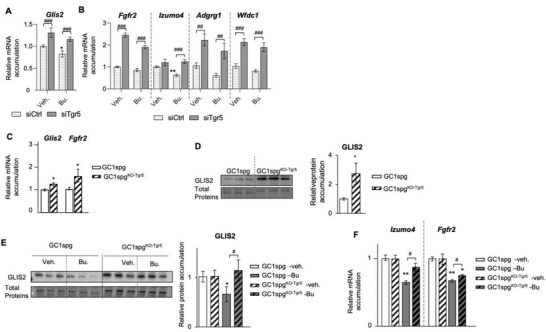
A) *Glis2* mRNA accumulation normalized to *β*‐actin on GC1spg cells transfected with siCtrl or siTgr5 and treated 6 h with vehicle or with 200 × 10^−6^ m of Bu. Vehicle‐treated siCtrlt‐transfected cells were arbitrarily set at 1. B) *Fgfr2*, *Izumo4*, *Adgrg1* and *Wfdc1* mRNA accumulation normalized to *β*‐actin on GC1spg cells transfected with siCtrl or siTgr5 and treated 24 h with vehicle or with 200 × 10^−6^ m of Bu. For quantification, vehicle‐treated siCtrlt‐transfected cells were arbitrarily set at 1. C) *Glis2* and *Fgfr2* mRNA accumulations normalized to *β*‐actin in vehicle treated GC1spg cells and GC1spg^KO‐Tgr5^. For quantification, GC1spg cells were arbitrarily set at 1. D) Representative western blots of GLIS2 in GC1spg cells and GC1spg^KO‐Tgr5^ cells treated with vehicle. Normalization was performed against total protein using stain‐free gels. For quantification, GC1spg cells were arbitrarily set at 1. E) Representative western blots of GLIS2 and quantification of ratios in GC1spg cells and GC1spg^KO‐Tgr5^ cells treated with vehicle or Bu for 24 h. Normalization was performed against total protein using stain‐free gels. Vehicle groups of each genotype were arbitrarily set at 1. F) *Izumo4* and *Fgfr2* mRNA accumulations normalized to *β*‐actin in GC1spg cells and GC1spg^KO‐Tgr5^ treated for 24 h with vehicle treated or Bu 200 × 10^−6^ m. Vehicle groups of each genotype were arbitrarily set at 1. In all panels, *n* = 9–26 from 3 to 5 independent experiments. Data are expressed as the means ± SEM. ANOVA2 followed by Holm‐Sidak's test for multiple comparisons. *, *p* < 0.05; **, *p* < 0.01 versus respective vehicle group for each genotype. #, *p* < 0.05; ##, *p* < 0.01; ###, *p* < 0.001 between genotypes exposed to same treatments. The horizontal square brackets underline the groups statistically compared between two conditions of different genotypes. Veh: vehicle and Bu: Busulfan.

Analysis of genes that were down‐regulated by Bu in siCtrl‐transfected cells (Table S3, Supporting Information), using Cis‐Target, showed that 53% (229/431) of these genes were associated with GLIS2 transcription factor, as defined by the identification of the DNA binding site (Figure S6A, Supporting Information). GLIS2 has previously been shown to be involved in maintaining stem cell pluripotency and is also associated with chemosensitivity.^[^ ^36^ ^]^ Interestingly, *Glis2* mRNA was itself down regulated by Bu in siCtrl‐transfected cells (Figure S6B, Supporting Information, data from RNAseq). Moreover, qPCR analyses validated the downregulation of *Glis2* expression 6 h after exposure to Bu in cells transfected with siCtrl, and these effects were less pronounced in siTgr5‐transfected cells (**Figure** **6**A). To ensure that the impact on GLIS2 had affected its downstream pathway, we then analyzed the GLIS2 target genes using qPCR. The impact of TGR5 signaling on the GLIS2 pathway was supported by qPCR analyses showing that the mRNA accumulations of *Fgfr2*, *Izumo4*, *Adgrg1*, and *Wfdc1* were modified by Bu specifically in cells transfected with siCtrl, with a lower impact of Bu in siTgr5‐transfected cells (Figure 6B). Note that the levels of these GLIS2 target genes were higher in siTgr5‐transfected cells compared to cells transfected with siCtrl in both veh‐ and Bu‐treated groups (Figure 6B). These data were confirmed using GC1spg^KO‐Tgr5^ cells with higher levels of *Glis2* and *Fgfr2* compared to GC1spg cells (Figure 6C). In addition, higher levels of GLIS2 protein were also observed in GC1spg^KO‐Tgr5^ cells compared to GC1spg cells (Figure 6D). A differential impact of Bu was observed on GLIS2 protein levels (Figure 6E) and on the mRNA accumulations of its target genes, namely *Izumo4* and *Fgfr2* (Figure 6F) between GC1spg and GC1spg^KO‐Tgr5^.

**Figure 7:**
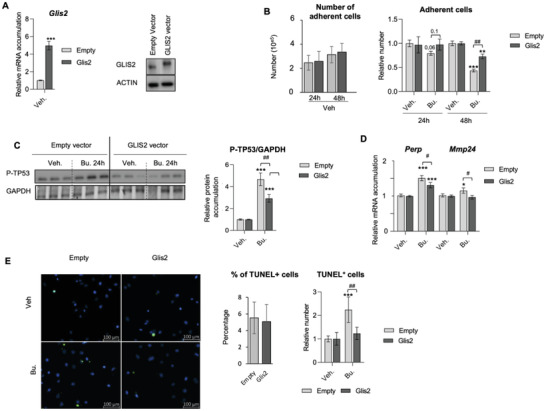
A) (Left) *Glis2* mRNA accumulation normalized to *β*‐actin on GC1spg cells transfected with an empty vector or a vector for overexpression of GLIS2. Cells transfected with empty vector were arbitrarily set at 1. (Right) Representative western blots of GLIS2 in GC1spg cells transfected with an empty vector or a vector for overexpression of GLIS2. B) (Left panel) Raw number of adherent cells transfected with an empty vector or a vector for overexpression of GLIS2 and treated with vehicle for 24 or 48 h. (Right panel) Relative number of adherent cells transfected with an empty vector or a vector for overexpression of GLIS2 and treated with vehicle or Bu for 24 or 48 h. Vehicle groups of each genotype were arbitrarily set at 1. C) Representative western blots of Phospho‐TP53 (P‐TP53), and quantification of ratios in GC1spg cells transfected with an empty vector or a vector for overexpression of GLIS2 and treated with vehicle or Bu for 24 h. Vehicle groups of each genotype were arbitrarily set at 1. D) *Perp* and *Mmp24* mRNA accumulation normalized to *β*‐actin in GC1spg cells transfected with an empty vector or a vector for overexpression of GLIS2 and treated with vehicle or Bu for 24 h. Vehicle groups of each genotype were arbitrarily set at 1. E) (Left) Representative micrographs of GC1spg cells transfected with an empty vector or a vector for overexpression of GLIS2 and treated with vehicle or Bu for 24 h and stained for TUNEL. (Right) Quantification of the raw number of the number of TUNEL positive GC1spg cells transfected with an empty vector or a vector for overexpression of GLIS2 and treated with vehicle 24 h. Vehicle groups of each genotype were arbitrarily set at 1 Quantification of the relative number of TUNEL positive GC1spg cells transfected with an empty vector or a vector for overexpression of GLIS2 and treated with vehicle or Bu for 24 h. Vehicle groups of each genotype were arbitrarily set at 1. In all panels, *n* = 5–26 from 3 to 5 independent experiments. Data are expressed as the means ± SEM. ANOVA2 followed by Holm‐Sidak's test for multiple comparisons. *,**, *p* < 0.01; ***, *p* < 0.001 versus respective vehicle group for each genotype. #, *p* < 0.05; ##, *p* < 0.01between genotypes exposed to same treatments. The horizontal square brackets underline the groups statistically compared between two conditions of different genotypes. Veh: vehicle and Bu: Busulfan.

To determine whether GLIS2 might be involved in germ cell sensitivity to Bu, GC1spg cells were transfected with a plasmid for GLIS2 overexpression to counteract the decrease in *Glis2* expression in response to Bu and to mimic the increase in GLIS2 expression observed in siTgr5‐transfected cells and in the GC1spg^KO‐Tgr5^ cells compared to control cells. Overexpression of GLIS2 was confirmed at mRNA and protein levels 24 h after transfection (**Figure** **7**A). Overexpression of GLIS2 led to a lower impact of Bu on the number of adherent cells at 24 and 48 h, compared to the control group (Figure 7B). In addition, a lower impact of Bu was observed on the accumulation of Phospho‐TP53 (P‐TP53) in cells overexpressing GLIS2 compared to control cells (Figure 7C). GLIS2 overexpression did not alter the impact of Bu on the TP53 protein levels (Figure S6C, Supporting Information). The qPCR analyses showed that GLIS2 overexpression reduced the impact of Bu on TP53 target genes, namely *Perp* and *Mmp24* (Figure 7D). GLIS2 overexpression was also associated with a lower rate of apoptosis in response to Bu, as identified by the TUNEL experiments (Figure 7E).

**Figure 8:**
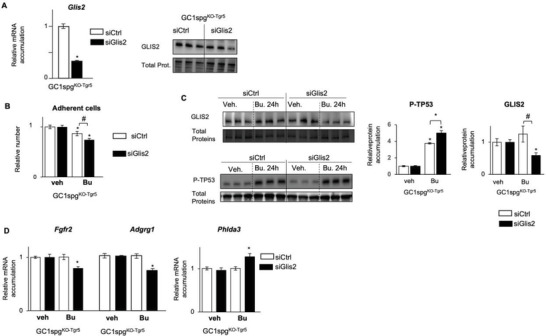
A) (Left panel) Relative Glis2 mRNA accumulation normalized to *β*‐actin on GC1spg^KO‐TGR5^ cells transfected with siCtrl or siGlis2. siCtrl‐transfected cells were arbitrarily set at 1. (Right panel) Representative western blots of GLIS2 in GC1spg^KO‐TGR5^ cells transfected with siCtrl or siGlis2. Normalization was performed against total protein using stain‐free gels. B) Number of adherent GC1spg^KO‐TGR5^ cells transfected with siCtrl or siGlis2 and treated with vehicle or Bu for 24 h. Vehicle treated cells of each genotype were arbitrarily set at 1. C) Representative western blots of GLIS2 and Phospho‐TP53 (P‐TP53), and quantification of ratios in GC1spg^KO‐Tgr5^ cells transfected with siCtrl or siGlis2 and treated with vehicle or Bu for 24 h. Normalization was performed against total protein using stain‐free gels. Vehicle groups of each genotype were arbitrarily set at 1. D) *Fgfr2*, *Adgrg1* and *Phlda3* mRNA accumulations normalized to *β*‐actin on GC1spg^KO‐TGR5^ cells transfected with siCtrl or siGlis2 and treated 24h with vehicle or with 200 × 10^−6^ m of Bu. Vehicle groups of each genotype were arbitrarily set at 1. In all panels, *n* = 10–15 from 3 to 5 independent experiments. Data are expressed as the means ± SEM. ANOVA2 followed by Holm‐Sidak's test for multiple comparisons. *, *p* < 0.05; **, *p* < 0.01 versus respective vehicle group for each genotype. #, *p* < 0.05 between genotypes exposed to same treatments. The horizontal square brackets underline the groups statistically compared between two conditions of different genotypes. Veh: vehicle and Bu: Busulfan.

GLIS2 was overexpressed in GC1spg^KO‐Tgr5^ cells (Figure 6D,E), and GLIS2 overexpression was implicated in Bu resistance (Figure 7); therefore, we hypothesized that Glis2 knock‐down might sensitize GC1spg^KO‐Tgr5^ cells to Bu. To answer this question, GC1spg^KO‐Tgr5^ cells were transfected with siRNA to decrease *Glis2* expression. The efficiency of the siRNA directed against *Glis2* was validated 24 h after transfection by qPCR and western blot analyses (**Figure** **8**A). Data showed that decreasing *Glis2* expression led to sensibilization of the GC1spg^KO‐Tgr5^ cells with a stronger impact of Bu at 24 h, as revealed by the lower relative number of adherent cells compared to the siCtrl transfected cell groups (Figure 8B). In addition, a stronger impact of Bu on the accumulation of Phospho‐TP53 (P‐TP53) was observed in si*Glis2* transfected‐cells compared to control cells (Figure 8C). In the same line of evidence, the re‐sensibilization of GC1spg^KO‐Tgr5^ cells to Bu following *Glis2* knock‐down was also revealed by lower GLIS2 protein accumulation levels in siGlis2‐transfected cells treated to Bu compared to vehicle group, whereas this effect of Bu was not observed in si‐Ctrl transfected GC1spg^KO‐Tgr5^ cells (Figure 8C). Then, the qPCR analyses showed that *Glis2* knock‐down led to a higher impact of Bu on its target genes *Fgfr2* and *Adgrg1*, and on the TP53 target gene, namely *Phlda3* in GC1spg^KO‐Tgr5^ cells (Figure 8D). These data define a crosstalk between GLIS2 and TP53 in response to Bu, which is controlled by TGR5. All these data clearly demonstrate that GLIS2 signaling plays a major role on the impacts of Bu.

### 2.7 The Absence of TGR5 Protects Germ Cells from the Effects of Other Chemodrugs

Taken together, the afore‐mentioned data suggest that TGR5 is involved in the chemosensitivity of germ cells. We then tested whether this impact of TGR5 signaling pathway could be extrapolated to other chemodrugs such as cisplatin,^[^ ^37^ ^]^ cyclophosphamide,^[^ ^12^ ^]^ treosulfan,^[^ ^38^ ^]^ or hepsulfam.^[^ ^39^ ^]^ Cyclophosphamide, treosulfan, and hepsulfam represent either alkylating agents and/or compounds that are used for similar clinical applications as Bu, even though their exact mechanisms have not yet been described. A first screen was performed in vitro using GC1‐spg cells and GC1spg^KO‐Tgr5^ cells. Data showed that the lack of *Tgr5* did not protect GC1spg cells from the deleterious effects of cisplatin (Figure S7A, Supporting Information). In contrast, results demonstrated that the lack of *Tgr5* decreased or counteracted the effects of treosulfan, cyclophosphamide and hepsulfam (Figure S7A, Supporting Information).

We next proceeded with in vivo analysis on *Wt* and *Tgr5^–/–^* mice and data showed that the effects of cyclophosphamide or hepsulfam exposures led to decreased number of PLZF+ cells in *Wt* mice 1 week after exposure (Figure S7B, Supporting Information). Interestingly, *Tgr5^–/–^* males showed lower impacts due to chemodrugs compared to *Wt* males (Figure S7B, Supporting Information).

Interestingly, data showed that hepsulfam treatment had a lower impact on P‐TP53 in the GC1spg^KO‐Tgr5^ cells, compared to GC1spg cells (Figure S7C, Supporting Information). It was observed that hepsulfam led to increased mRNA accumulations of TP53 target gene such as *Mmp24* in GC1‐spg cells specifically, and these effects were not observed in GC1spg^KO‐Tgr5^ cells (Figure S7D, Supporting Information). In addition, a slight but significant decrease of *Glis2* mRNA accumulation was observed in GC1‐spg cells treated with hepsulfam (Figure S7E, Supporting Information). This impact was confirmed at the protein level (Figure S7C, Supporting Information).

### 2.8 In Vivo Analyses Validate the In Vitro Data

**Figure 9:**
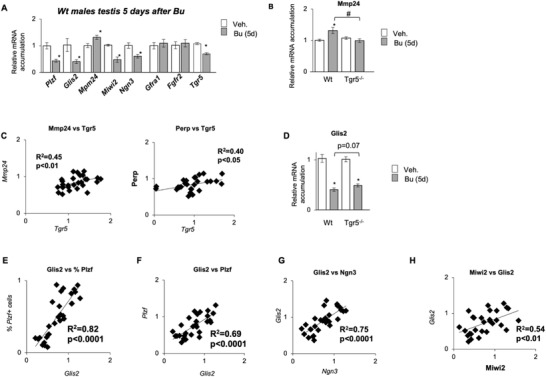
A) *Plzf*, *Glis2*, *Mmp24*, *Miwi2*, *Ngn3*, *Gfra1*, *Fgfr2* and *Tgr5* mRNA accumulations normalized to *β*‐actin in testis of Wt mice 5 days after the treatment with vehicle or Bu. In panel, *n* = 10 to 15 from 3 independent experiments. Vehicle groups of each genotype were arbitrarily set at 1. Data are expressed as the means ± SEM. Statistical analysis: *, *p* < 0.05 versus respective vehicle group for each genotype. Veh: vehicle and Bu: Busulfan. B) *Mpm24* mRNA accumulations normalized to *β*‐actin in testis of Wt and Tgr5^–/–^ mice 5 days after the treatment with vehicle or Bu. In panel, *n* = 10 to 15 from 3 independent experiments. Vehicle groups of each genotype were arbitrarily set at 1. Data are expressed as the means ± SEM. ANOVA2 followed by Holm‐Sidak's test for multiple comparisons. *, *p* < 0.05 versus respective vehicle group for each genotype. #, *p* < 0.05 between genotypes exposed to same treatments. The horizontal square brackets underline the groups statistically compared between two conditions of different genotypes. Veh: vehicle and Bu: Busulfan. C) Correlation analyses of the mRNA levels of *Tgr5* with *Mmp24* or *Perp* normalized to *β*‐actin in testis of Wt mice 5 days after the treatment with vehicle or Bu. In panel, *n* = at least 15 from 3 independent experiments. Spearman Statistical analysis: *, *p* < 0.05. D) *Glis2* mRNA accumulations normalized to *β*‐actin in testis of *Wt* and *Tgr5^–/–^* mice 5 days after the treatment with vehicle or Bu. In panel, *n* = 15 from 3 independent experiments. Vehicle groups of each genotype were arbitrarily set at 1. Data are expressed as the means ± SEM. ANOVA2 followed by Holm‐Sidak's test for multiple comparisons. *, *p* < 0.05 versus respective vehicle group for each genotype. The horizontal square brackets underline statistical analysis between two conditions of different genotypes. Veh: vehicle and Bu: Busulfan. E) Correlation analyses of the mRNA levels of *Glis2* with the % of PLZF + cells in testis of Wt mice 5 days after the treatment with vehicle or Bu. In panel, *n* = at least 15 from 3 independent experiments. Spearman Statistical analysis: *, *p* < 0.05. F) Correlation analyses of the mRNA levels of *Glis2* with Plzf mRNA accumulations normalized to *β*‐actin in testis of Wt mice 5 days after the treatment with vehicle or Bu. In panel, *n* = at least 15 from 3 independent experiments. Spearman Statistical analysis: *, *p* < 0.05. G) Correlation analyses of the mRNA levels of *Glis2* with *Ngn3* mRNA accumulations normalized to *β*‐actin in testis of Wt mice 5 days after the treatment with vehicle or Bu. In panel, *n* = at least 15 from 3 independent experiments. Spearman Statistical analysis: *, *p* < 0.05. H) Correlation analyses of the mRNA levels of *Glis2* with *Miwi2* mRNA accumulations normalized to *β*‐actin in testis of Wt mice 5 days after the treatment with vehicle or Bu. In panel, *n* = at least 15 from 3 independent experiments. Spearman Statistical analysis: *, *p* < 0.05.

To validate the molecular targets defined in vitro in the GC1spg cell line and thus evaluate the main role of TGR5 in germ cells, we used in vivo samples. We analyzed the expression of markers in Bu‐treated testis even if we were aware of the potential bias due to the alteration of cellularity induced by Bu. The present analysis showed that in early timing (5 days after Bu exposure), the data supported in vitro findings, showing a lower mRNA accumulation of *Tgr5*, *Gfra1*, *Plzf*, and *Glis2*, in addition to *Ngn3* and *Miwi2* in *Wt* mice treated with Bu, compared to the group treated with vehicle (**Figure** **9**A). In addition, a higher mRNA accumulation of *Mmp24* was observed in the Bu treated males compared to vehicle treated mice (Figure 9A).

The data demonstrated that the initial loss of germ cell was similar between *Wt* and *Tgr5^–/–^* mice up to 5 days post‐treatment (Figure S2A, Supporting Information), allowing us to compare data between *Wt* and *Tgr5^–/‐^* males. The present data showed that *Mmp24* mRNA accumulation was less impacted in the testis of *Tgr5^–/–^* males following Bu exposure, compared to *Wt* males (Figure 9B). In addition, in the *Wt* busulfan treated males (for 1 d to 5 d), the mRNA accumulation of *Tgr5* was correlated with the expression of *Mmp24* and *Perp* (respectively, R^2^ = 45; *p* < 0.01 and R^2^ = 039; p < 0.05) (Figure 9C). This was consistent with the fact that there was lower level of apoptosis in *Tgr5^–/–^* males following Bu exposure.

qPCR analyses on the whole testis showed only a modest trend for a slightly lower impact of Bu on *Glis2* mRNA accumulation in the testis of *Tgr5^–/–^* males compared to Wt males (Figure 9D); as following Bu exposure a 60% decrease of *Glis2* mRNA accumulation was observed in Wt testis compared to a 50% decrease in *Tgr5^–/–^* testis (Figure 9D). However, data showed that GLIS2 may have a major role in the spermatogonia cell population. Indeed, the *Glis2* expression was highly correlated with the number of PLZF+ cells (R^2^ = 0.82; P<0.0001) and with the *Plzf* mRNA (R^2^ = 0.69; P<0.0001) (Figure 9E,F). This highlighted the main role of GLIS2 in the response to Bu in testis, as was defined in vitro. Interestingly, the mRNA levels of *Glis2* were correlated with those of *Ngn3* (R^2^ = 0.75; P<0.0001) (Figure 9G) and with the expression of *Miwi2* (R^2^ = 0.54; P<0.01) (Figure 9H). These data highlighted a potential link between GLIS2 and the progenitor population of spermatogonia, which had been described to participate in the recovery following injury.^[^ ^4^, ^5^ ^]^ *Ngng3* and *Miwi2* mRNA accumulations were correlated to each other (Figure S8, Supporting Information). Additionally, *Ngng3* and *Miwi2* mRNA levels were correlated with the relative percentage of PLZF+ cells (R^2^ = 0.69P<0.0001 and R^2^ = 0.43; P<0.01 respectively) and with the expression of *Plzf* (R^2^ = 0.52; P<0.01 and R^2^ = 0.52; P<0.0001) (Figure S8, Supporting Information). Interestingly, the *Stra8* mRNA accumulation was correlated with the relative percentage of PLZF+ cells, with the expression of *Plzf*, of *Ngn3* and of *Miwi2* (Figure S9, Supporting Information). Moreover, the mRNA accumulation of *Glis2* (R^2^ = 0.78; P<0.0001) was correlated with the mRNA level of *Stra8* (Figure S9, Supporting Information), suggesting the potential association with germ cell differentiation and enhanced maintenance of germ cell lineage.

All these data sustained the idea that TGR5 pathways are associated with regenerative capacity of germ cells in part through the interaction with GLIS2 and TP53 pathways. Our data also support the idea of a putative crosstalk with the Ngn3‐Miwi2 pathway in progenitor cells that could participate to expand the pool of spermatogonial cells with stem cell activity under regenerative conditions.

All the present in vivo data supported the in vitro observations. To better decipher the crosstalk between TGR5 and Bu, we performed analyses at 1‐week after Bu exposure, when the number of PLZF+ cells was different between genotypes. To avoid the bias of cellularity induced by Bu between genotypes, we analyzed the expression of markers of SSC (*Gfra1*; *Fgfr2*) and progenitors (*Ngn3* and *Miwi2*) in the THY1+ purified spermatogonia.

**Figure 10:**
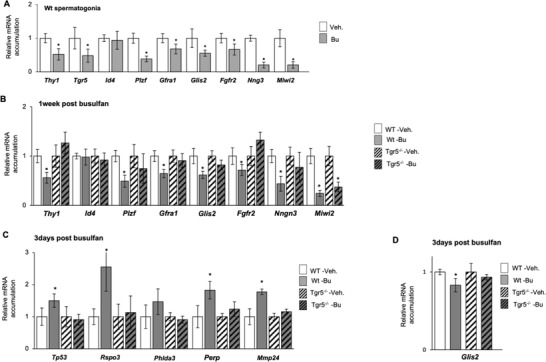
A) *Thy1*, *Tgr5*, *Id4*, *Plzf*, *Gfra1*, *Glis2, Fgfr2*, Ngn3 and Miwi2 mRNA accumulations normalized to *β*‐actin in THY1+ isolated spermatogonia of adult Wt mice 1 week after the treatment with vehicle or Bu. In panel, *n* = 10 to 15 from 3 independent experiments. Vehicle groups of each genotype were arbitrarily set at 1. Data are expressed as the means ± SEM. statistical analysis: *, *p* < 0.05 versus respective vehicle group for each genotype. Veh: vehicle and Bu: Busulfan. B) *Thy1*, *Id4*, *Plzf*, *Gfra1*, *Glis2*, *Fgfr2*, *Ngn3*, and *Miwi2*, mRNA accumulations normalized to *β*‐actin in THY1+ isolated spermatogonia of adult Wt and Tgr5*^–/‐^* mice 1 week after the treatment with vehicle or Bu. In panel, *n* = 10 to 15 from 3 independent experiments. Vehicle groups of each genotype were arbitrarily set at 1. Data are expressed as the means ± SEM. ANOVA2 followed by Holm‐Sidak's test for multiple comparisons. *, *p* < 0.05 versus respective vehicle group for each genotype. Veh: vehicle and Bu: Busulfan. C) *Tp53*, *Rspo3*, *Phlda3*, *Perp* and *Mmp24* mRNA accumulations normalized to *β*‐actin in THY1+ isolated spermatogonia of adult Wt and Tgr5*^–/‐^* mice 3 days after the treatment with vehicle or Bu. In panel, *n* = 5. Vehicle groups of each genotype were arbitrarily set at 1. Data are expressed as the means ± SEM. ANOVA2 followed by Holm‐Sidak's test for multiple comparisons. *, *p* < 0.05 versus respective vehicle group for each genotype. Veh: vehicle and Bu: Busulfan. D) *Glis2* mRNA accumulations normalized to *β*‐actin in isolated spermatogonia of adult Wt and Tgr5*^–/‐^* mice 3 days after the treatment with vehicle or Bu. In panel, *n* = 5. Vehicle groups of each genotype were arbitrarily set at 1. Data are expressed as the means ± SEM. ANOVA2 followed by Holm‐Sidak's test for multiple comparisons. *, *p* < 0.05 versus respective vehicle group for each genotype. Veh: vehicle and Bu: Busulfan.

Data showed that 1‐week after Bu exposure, the mRNA accumulation of *Tgr5* in *Wt* males was decreased in THY1+ cells (**Figure** **10**A), consistent to what was observed in GC1‐spg cell line. One week after exposure, all the analyzed markers such as *Thy1*, *Gfra1*, *Fgfr2*, and *Plzf*, in addition to *Ngn3* and *Miwi2*, were downregulated in the spermatogonia isolated from Bu‐treated *Wt* males compared to their respective control group (Figure 10A,B); a lower effect of Bu was observed in *Tgr5^–/–^* males (Figure 10B).

Interestingly, in the THY1+ population, the impacts of Bu on spermatogonia were associated with an increase of TP53 signaling pathway 3 days after Bu exposure, as revealed by the mRNA accumulations of *Tp53* and of its target genes such as *Mmp24*, *Phlda3*, *Perp*, and *Rspo3* (Figure 10C). For these targets, an impact of Bu was observed in *Wt* spermatogonia, whereas lower impacts of Bu were observed on Tgr5^–/‐^ spermatogonia (Figure 10C).

This was also associated with an alteration of GLIS2 pathway by Bu, with a decrease in the mRNA accumulations of *Glis2* at both 3 days and 1 week following Bu exposure (Figure 10B,D). Additionally, the GLIS2 target gene (*Fgfr2*) was altered in *Wt* spermatogonia only at 1 week after treatment and not at 3 days post‐treatment (Figure 10A,B; Figure S10A, Supporting Information). No effect was observed in Tgr5^–/‐^ spermatogonia (Figure 10B). Such a delay between GLIS2 and its targets was also observed in GC1spg as alteration of *Tgr5* and *Glis2* mRNA accumulations were concomitantly noticed at 6 and 12 h after Bu administration, whereas GLIS2 target genes such *Fgfr2*, *Izumo4*, and *Wfdc1* were altered only at 12 or 24 h (Figure S10B, Supporting Information).

Taken together, all these data supported the validity of the data obtained from the GC1spg cell line and confirmed, in vivo, the link and key role of the TGR5; GLIS2 and TP53 pathways in germ cells in response to Bu.

### 2.9 Regulation of GLIS2 and P‐TP53 by PKA Explains the Interaction between TGR5 and Bu Signaling Pathways

TGR5 is known to act through PKA, and it has been demonstrated previously that the expression of CREB1 was lowered in the brown adipose tissue of *Tgr5^–/‐^* mice, compared to that in the *Wt* mice.^[^ ^34^ ^]^

**Figure 11:**
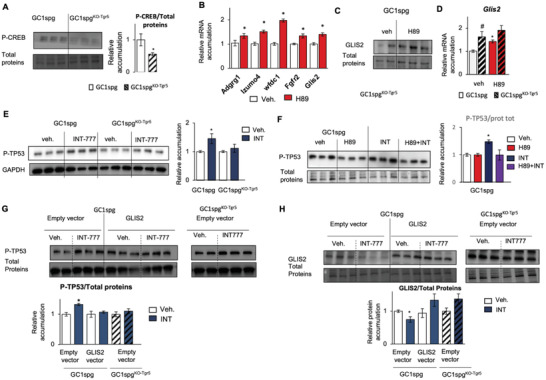
Representative western blots of Phospho‐CREB (P‐CREB) and quantification of ratios in GC1spg and GC1spg^KO‐Tgr5^. GC1spg cells were arbitrarily set at 1. *Adgrg1*, *Izumo4*, *Wfdc1*, *Fgfr2* and *Glis2* mRNA accumulations normalized to *β*‐actin in GC1spg cells treated for 45 min with vehicle or H89 and harvested 24 h later. Vehicle groups were arbitrarily set at 1. Representative western blots of GLIS2 and quantification ratios in GC1spg cells treated with vehicle or H89 for 45 min and harvested 24 h later. Normalization was performed against total protein using stain‐free gels. *Glis2* mRNA accumulations normalized to *β*‐actin in GC1spg and GC1spg^KO‐Tgr5^ cells treated for 45 min with vehicle or H89 and harvested 24 h later. Vehicle treated GC1spg cells were arbitrarily set at 1 Representative western blots of Phospho‐TP53 (P‐TP53), and quantification of ratios in GC1spg and GC1spg^KO‐Tgr5^ cells treated with vehicle or INT‐777 for 24h. Vehicle treated cells were arbitrarily set at 1. Representative western blots of Phospho‐TP53 (P‐TP53), and quantification ratios in GC1spg cells treated with vehicle or H89 for 45 min and then with vehicle or INT‐777 for 24 h. Vehicle treated cells were arbitrarily set at 1. Normalization was performed against total protein using stain‐free gels. Representative western blots of Phospho‐TP53 (P‐TP53), and quantification of ratios in GC1spg cells transfected with an empty vector or a vector for overexpression of GLIS2 and treated with vehicle or INT‐777 (25 × 10^−6^ m) for 24 h as well as GC1spg^KO‐Tgr5^ cells transfected with an empty vector and treated with vehicle for 24 h. Normalization was performed against total protein using stain‐free gels. Vehicle treated cells were arbitrarily set at 1. Representative western blots of GLIS2, and quantification of ratios in GC1spg cells transfected with an empty vector or a vector for overexpression of GLIS2 and treated with vehicle or INT‐777 (25 × 10^−6^ m) for 24 h as well as GC1spg^KO‐Tgr5^ cells transfected with an empty vector and treated with vehicle for 24 h. Normalization was performed against total protein using stain‐free gels. Vehicle treated cells were arbitrarily set at 1. In all panels, *n* = 15 from 3 independent experiments. Data are expressed as the means ± SEM. ANOVA2 followed by Holm‐Sidak's test for multiple comparisons. *, *p* < 0.05 versus respective vehicle group for each genotype. Veh: vehicle and Bu: Busulfan.

Analysis using Genomatix of the available human and mouse *Glis2* regulatory sequences referenced led us to identify potential CREB binding sites (Figure S11, Supporting Information), supporting the idea that TGR5 could modulate *Glis2* expression at the promoter level through PKA pathway. This was consistent with the fact that *Glis2* was defined as one of the target genes of the **CREB1** transcription factor in ChIP‐seq datasets from the ENCODE Transcription Factor Targets dataset.^[^ ^40^, ^41^ ^]^ These data highlighted a potential TGR5‐PKA‐cAMP‐GLIS2 signaling cascade. In that line, the present data show lower P‐CREB levels in GC1spg^KO‐Tgr5^ cells than in GC1spg cells (**Figure** **11**A). We questioned whether TGR5 could regulate *Glis2* and its target genes through the PKA pathway. Interestingly, the data showed that PKA inhibition with H89 led to an increase in mRNA accumulation of *Glis2* and its target genes (Figure 11B). The impact of H89 on GLIS2 was confirmed at the protein level in GC1spg cells (Figure 11C). H89 treatment resulted in a significant 1.5‐fold increase in Glis2 mRNA accumulation in GC1spg cells (Figure 11D). These data suggest that the increased accumulation of Glis2 mRNA was associated with inhibition of the PKA pathway. Of note, H89 had no effect on Glis2 mRNA accumulation in GC1spg^KO‐Tgr5^ cells compared with vehicle‐treated GC1spg^KO‐Tgr5^ cells (Figure 11D). Interestingly, as previously shown in vehicle‐treated cells, Glis2 mRNA accumulation was significantly greater in GC1spg^KO‐Tgr5^ cells than in GC1spg cells (Figure 11D). This result was consistent with the lower levels of phosphorylated CREB1 in GC1spg^KO‐Tgr5^ cells compared with GC1spg cells (Figure 11A); this may reflect weaker PKA activation in GC1spg^KO‐Tgr5^ cells and explain how Glis2 mRNA accumulation was higher in GC1spg^KO‐Tgr5^ cells compared with GC1spg cells.

The association among TGR5, PKA, GLIS2, and P‐TP53 was further supported by data showing that INT‐777 treatment led to an increase in P‐TP53 (Figure 11E), which was counteracted by PKA inhibition (H89) (Figure 11F) and by the overexpression of GLIS2 (Figure 11G). Note that INT‐777 treatment led to a decrease of GLIS2 protein accumulation in GC1‐spg cells, whereas no effect was observed in GC1spg^KO‐Tgr5^ cells (Figure 11H).

Taken together, these data suggested that TGR5 regulates GLIS2 through the PKA pathway, which then regulated TP53 phosphorylation. These data suggested that the crosstalk between TGR5 and TP53 must be at least at the post‐translational level via PKA and GLIS2.

We have demonstrated that the inhibition of PKA, and thus P‐CREB levels, by H89 administration leads to an increase in GLIS2 levels. The lower P‐CREB levels in GC1spg^KO‐Tgr5^ cells than in GC1spg cells allows for re‐examination of the effects of Bu and the link to TGR5 signaling, and the potential involvement of PKA signaling, thus leading to the overexpression of GLIS2 in connection with the lower impact of Bu in GC1spg^KO‐Tgr5^ cells.

### 2.10 Activation of TGR5 Sensitized Cells to Bu

**Figure 12:**
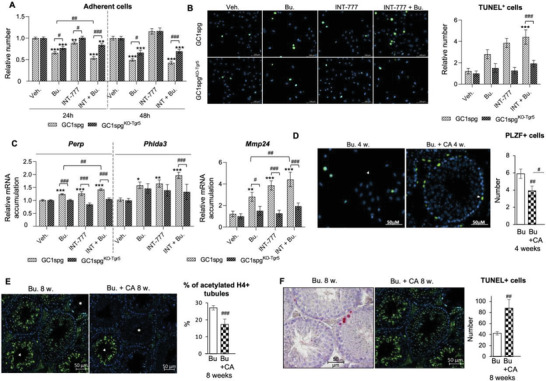
A) Relative number of adherent cells in GC1spg and GC1spg^KO‐Tgr5^ cells pre‐exposed 24h with INT‐777 and then to Bu (200µM) for 24 or 48 h. Vehicle treated cells were arbitrarily set at 1. B) (Left) Representative micrographs of GC1spg and GC1spg^KO‐Tgr5^ cells pre‐exposed 24h with INT‐777 and then to Bu 200µM for 48 h and stained for TUNEL. (Right) Quantification of the relative number of TUNEL positive GC1spg and GC1spg^KO‐Tgr5^ cells pre‐exposed 24h with INT‐777 and then with Bu (200µM) for 48 h. Vehicle treated cells were arbitrarily set at 1. C) *Perp*, *Phld3a* and *Mmp24* mRNA accumulations normalized to *β*‐actin in GC1spg cells and GC1spg^KO‐Tgr5^ cells pre‐exposed 24h with INT‐777 and then to Bu 200µM for 24 h. Vehicle treated cells were arbitrarily set at 1. In A to C panels, *n* = 6–24 from 3 independent experiments. Data are expressed as the means ± SEM. ANOVA2 followed by Holm‐Sidak's test for multiple comparisons. **, *p* < 0.01, ***, *p* < 0.001 versus respective vehicle group for each genotype. #, *p* < 0.05; ##, *p* < 0.01; ###, *p* < 0.001 between genotypes exposed to same treatments. The horizontal square brackets underline the groups statistically compared between two conditions of different genotypes. Veh: vehicle and Bu: Busulfan. (D) (Left) Representative micrographs of testis of Bu or Bu+CA treated Wt males stained for PLZF. (Right) Quantification of the number of positive PLZF cells per seminiferous tubule of males treated with the Bu or Bu+CA (4 weeks after treatment). E) (Left) Representative micrographs of testis of Bu or Bu+CA treated Wt males stained for acetylated H4. (Right) Quantification of the number of acetylated H4 positive seminiferous tubules of males treated with the Bu or Bu+CA (8 weeks after treatment). F) (Left) Representative micrographs of testis of Bu or Bu+CA treated Wt males stained for TUNEL. (Right) Quantification of the number of TUNEL positive cells per seminiferous tubule of testes from Wt males treated with Bu or Bu+CA (8 weeks after treatment). In D to F panels, *n* = 10–24 from 3 independent experiments. Data are expressed as the means ± SEM. Statistical analysis: *, *p* < 0.05; ** *p* < 0.01; *** *p* < 0.001 versus Bu treated group. Bu: Busulfan and Bu+CA: Busulfan+ cholic acid.

The regulation of GLIS2 and P‐TP53 by PKA explain the crosstalk between the TGR5 and Bu signaling pathways. Because the absence of TGR5 protects germ cells from the long‐term effects of Bu, we decided to analyze the impact of TGR5 activation using its agonist INT‐777 in combination with Bu. GC1‐spg cells were pretreated for 24 h with INT‐777 and then with Bu for 24 or 48 h, and the data were compared to cells exposed to Bu alone. As was shown previously, there was no statistical difference between cell number in vehicle condition between GC1spg and GC1spg^KO‐Tgr5^ cells; therefore, we set vehicle groups at 1 to better decipher the effects of the molecules. The results showed that cell counts were lower when GC1spg cells were exposed to INT‐777 and Bu, compared to when they were exposed to Bu for 24 and 48 h (**Figure** **12**A). This additive effect of INT and Bu was not observed in GC1spg^KO‐Tgr5^ cells (Figure 12A).

To explain the lower number of adherent cells in the INT+Bu condition compared to Bu, we examined whether the activation of TGR5 by INT‐777 could additively with Bu modified the proliferation/apoptotic balance. No effect of INT+Bu was observed on proliferation as revealed by BrdU incorporation (Figure S12A, Supporting Information). Interestingly, at 48 h after Bu‐treatment, a significantly higher increase in apoptosis cell rates was observed in GC1spg *Wt* cells treated by both Bu and INT‐777 compared to Bu, INT or Veh (Figure 12B). Note that no significant effect was observed in the GC1spg^KO‐Tgr5^ (Figure 12B).

In that line, additive alterations in mRNA accumulations of the TP53 target genes by INT‐777 and Bu compared to Bu alone were observed at 24 h for *Perp*, *Phld3a*, and *Mmp24* (Figure 12C). All these effects were mediated by TGR5, as was revealed using GC1spg^KO‐Tgr5^ cells (Figure 12).

To confirm the involvement of the TGR5 signaling pathway, we performed similar experiments using another TGR5 agonist, namely oleanolic acid (OA), which acts through TGR5 in GC1spg cells; this was demonstrated using the cAMP‐response element fused to luciferase (Figure S4F, Supporting Information). Consistently, co‐exposure to Bu and OA led to additive effects on the number of adherent GC1spg, which were found to decrease (Figure S12B, Supporting Information). A small additive effect of Bu and OA was observed on P‐TP53 levels (Figure S12C, Supporting Information) and on mRNA accumulation of the TP53 target genes *Mmp24* and *Phld3a* (Figure S12D, Supporting Information). The level of apoptotic cell rate was higher in Bu+OA treated cells compared to cells treated with Bu alone (Figure S12E, Supporting Information).

Based on the results obtained upon treating GC1spg cells with a combination of Bu, INT‐777, or OA, we speculated that a combination of Bu and bile acids (endogenous TGR5 ligands) could lead to synergistic deleterious effects on germ cell in vivo. Wild‐type males were treated once with Bu at the beginning of the experiment, and received a diet supplemented with 0.5% cholic acid (CA) for 8 weeks. Male mice treated with Bu and sacrificed after 4 weeks showed an almost complete recovery of PLZF+ cell counts (Figure 12D). In contrast, we observed a decrease of around 50% in the number of PLZF+ cells in mice treated with Bu+CA at 4 weeks after treatments (Figure 12D). Consistently, 8 weeks after Bu treatment, the number of seminiferous tubules with positive acetylated‐H4 spermatid cells was lower in the Bu+CA treated group than in Bu treated group (Figure 12E). This prolonged germ cell loss in the Bu+CA group 8 weeks after treatment was associated with a higher rate of apoptotic cells compared to the Bu‐treated group (Figure 12F). These data confirmed the interactions between BA‐TGR5 and Bu signaling pathways.

## 3 Discussion

Recently, bile acid homeostasis has been demonstrated to play some roles in controlling male fertility. The nuclear receptor of bile acid, namely FXR*α*, plays a key role in the homeostasis of undifferentiated germ cells as the FXR*α* knockout mice presented an increased number of undifferentiated germ cells throughout their postnatal life.^[^ ^24^ ^]^ This was associated with the maintenance of reproductive capacities during aging. Moreover, data obtained from mice have shown that the G‐protein coupled bile acid receptor TGR5 is involved in cholestasis‐induced fertility disorders.^[^ ^27^ ^]^ Under physiological conditions, no difference was observed between *Wt* and *Tgr5^–/–^* males. In this study, we used mice treated with Bu as a classical model of transient germ cell depletion to demonstrate that the TGR5 signaling pathway plays critical roles in germ cell homeostasis following injuries.

Regarding the responsiveness to Bu, the present work showed that Bu acted primarily on undifferentiated spermatogonial cells, which was in agreement with the results from other studies.^[^ ^14^ ^]^ Bu led to a decrease in the number of undifferentiated germ cells during the first days following treatment. In turn, this effect on undifferentiated germ cells led to a major impact on spermatocytes, which then affected spermatids and sperm cells, thereby affecting fertility. This confirmed that a Bu‐based study design can help understand the properties of undifferentiated germ cells. It has to be noted that this approach relied on single injection, in contrast to clinical conditions where patients undergo chronic exposure to Bu, in combination with fludarabine or cyclophosphamide. Bu is given at a dose of 3.2 mg/kg, which is repeated over 2 to 4 consecutive days (around 12 mg/kg as total dose).^[^ ^42^ ^]^ Thus, the final dose used in this study (15 mg/kg) was consistent with what is used in the clinic, although it is possible that there is a difference between acute and chronic exposure.

The impact of chemotherapeutic treatments depends on their bioavailability, which depends on their elimination and the various detoxification mechanisms involved. The half‐life of Bu is approximately 3 h, and therefore, it seems unlikely that TGR5 is involved in Bu detoxification as the initial effects of Bu on testicular physiology and on germ cell lineage are almost similar between *Wt* and *Tgr5^–/–^* males. Differences are observed between genotypes mainly at the time of germ cell lineage recovery, resulting from the differential ability of *Tgr5^–/‐^* undifferentiated germ cells to proliferate, survive, and/or differentiate. Thus, the present data demonstrate that TGR5 is a key factor for cellular repair and tissue regeneration following injuries.

Regarding undifferentiated spermatogonia, the present results demonstrated that Bu exposure decreased *Gfra1* and *Fgfr2* mRNA accumulation, and these effects were lowered in a low TGR5 state. Both FGFR2 and GFRA1 were defined as critical actors of the self‐renewal of spermatogonial stem cells.^[^ ^43^, ^44^ ^]^ Moreover, recent data highlighted the role of FGFR2 in inducing germ cell differentiation.^[^ ^45^ ^]^ All these data support the fact that TGR5 may play a critical role in the maintenance of the SSC pool and in the mechanisms of cell fate and regeneration following injuries, as illustrated here using anti‐cancer therapies. This may explain in part how the germ cells of *Tgr5^–/–^* males were able to rapidly initiate a new wave of spermatogenesis to repopulate the seminiferous tubules and give birth to offspring.

Next to the SCC pool, it has been demonstrated that the NGN3+ spermatogonial progenitor population retain stem cell potential to contribute to regeneration after injury.^[^ ^6^ ^]^ Moreover, after injury, within the NGN3+ population, MIWI2‐expressing cells exhibited stem cell activity that is essential for the efficient regenerative capacity of the testis. The data from this study identified strong correlations between *Glis2* and *Tgr5* with *Ngn3* and *Miwi2*, reinforcing the identification of molecular mechanisms resulting in germ cell re‐emergence after chemotherapy‐induced injury. Thus, TGR5 might act on either SCC and/or progenitor spermatogonia, affecting germ cell and fertility recovery after injury.

**Figure 13:**
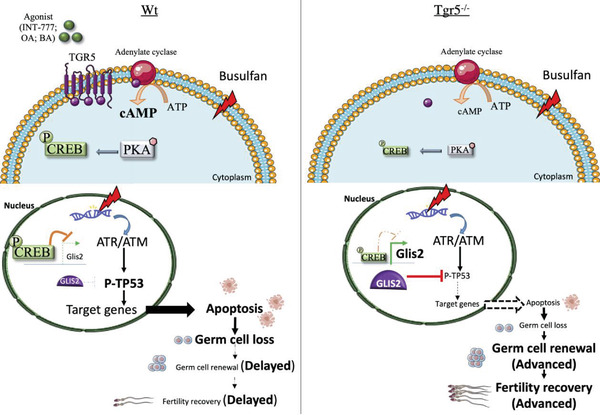
Proposed model for the crosstalk between TGR5 and Bu signaling pathways.

At the molecular level, we demonstrated the alterations of apoptotic process as one of the main mediators of the effects of Bu on germ cells. Analysis of RNAseq data allowed us to identify two key pathways associated with TGR5, namely GLIS2 and TP53. In addition, the data demonstrate the link between GLIS2 and TP53 in germ cell sensitivity to Bu. We used genetic tools and pharmacological approaches to demonstrate that this GLIS2‐TP53 pathway is regulated by TGR5. Furthermore, all the data presented confirm the primary role of TGR5 in the impact of Bu via the GLIS2‐TP53 pathway. To date, very few links have been established between GLIS2 and TP53. However, the model in this study is consistent with a previous study suggesting that the absence of GLIS2 stabilizes the accumulation of TP53 and the post‐translational modulation of TP53.^[^ ^46^ ^]^ Therefore, the results of the present study led us to derive a scheme connecting the TGR5 and Bu signaling pathways (**Figure** **13**). Bu has been demonstrated to induce TP53 phosphorylation. Parallelly, activation of TGR5 by agonists led to the activation of PKA and phosphorylation of CREB. This was associated with the repression of GLIS2 accumulation, which was consistent with the fact that GLIS2 was defined as a CREB1 target gene.^[^ ^40^ ^]^ The lower expression of GLIS2 in Bu treated‐conditions allows for a higher accumulation of P‐TP53, which was then associated with germ cell apoptosis. This substantial germ cell loss delayed the capacity of germ cell lineage emergence and consequently, male fertility. In contrast, in the context of TGR5 knock‐out or knock‐down, the lower activation of the PKA/CREB pathway was associated with higher levels of GLIS2 accumulation; this inhibited the accumulation of P‐TP53 induced by Bu and lowered germ cell apoptosis. This allowed for germ cell renewal and the earlier recovery of male fertility.

It is interesting to note that the activation of TP53 has also been associated with an increase in lipid peroxidation in Bu‐treated testes after 1–2 weeks,^[^ ^12^, ^13^ ^]^ suggesting an increase in reactive oxygen species (ROS) production. Indeed, mice treated with melatonin after Bu injection show enhanced spermatogenesis.^[^ ^47^ ^]^ Melatonin drives the expression of MnSOD (MaNganese SuperOxide Dismutase), which counteracts the apoptosis caused by high levels of Bu‐induced ROS.^[^ ^14^ ^]^ In parallel, melatonin stimulates the expression of SIRT1, which participates in TP53 deacetylation, leading to TP53 degradation and resulting in cellular resistance to apoptosis. Although we did not detect lipid peroxidation or ROS‐related gene clusters in the RNA‐seq approach, the involvement of such a mechanism cannot be completely excluded because busulfan was found to modulate the level of acetylated‐TP53 (ac‐TP53) and this impact was lowered in siTGR5‐transfected cells (Figure S13A, Supporting Information). However, this effect is not a major mechanism because GLIS2 overexpression, which counteracts the impact of Bu on cell apoptosis, has no impact on the Bu‐induced effect on ac‐TP53 (Figure S13B, Supporting Information).

All the results presented in this study show that TGR5 plays a major role in testicular physiopathology. Invalidation of the *Tgr5* gene, or its inactivation by an antagonist, could facilitate protection of the germ line to limit the deleterious impacts of chemotherapy on fertility. Even though there is still a long way to go before it is clinically used, we propose that TGR5 modulation could be integrated into anti‐cancer treatment protocols. This modulation will need to be vectorized to induce a modulation of TGR5 specifically in the tissue of interest, thus avoiding effects on other cell types. However, the role of TGR5 in testicular physiology and pathophysiology remains to be fully understood.

Chemotherapies such as Bu, like other alkylating agents, will cause DNA damages by forming intra‐strand bridges in the DNA, leading to DNA breaks, thereby causing cell death.^[^ ^48^ ^]^ Their impact will depend on the type of chemotherapy and dose used. In addition to impacting the number of spermatozoa, chemotherapies could also have a major impact on their quality, because they can cause mutations in the germ cells.

The results of this study demonstrate that *Tgr5* invalidation minimizes the long‐term effects of Bu by causing an early restart of spermatogenesis, leading to an early return of fertility. However, it would be important to study sperm quality in *Tgr5^–/–^* individuals after Bu treatment. Because spermatogenesis is restored earlier in Tgr5^–/–^ males compared to control mice, it is important to avoid potential problems related to DNA repair. The cells rejuvenate faster in Tgr5^–/–^ males, but this could be associated with poor DNA repair, leading to defects in sperm quality that may result in developmental defects in the offspring. The fertility analyses performed in this study showed that 6 to 8 weeks after Bu treatment, all animals (*W*t or Tgr5^–/–^) were sterile. When spermatogenesis was restored, Bu‐treated Tgr5^–/–^ males, which were then bred with an untreated female after 20 weeks, gave birth to a normal number of offspring per litter without major impact on the number of fetuses that died in utero. However, it is conceivable that Bu may have an impact on sperm quality, which could translate into an impact on the offspring. The fact that TGR5‐mediated apoptosis leads in part to delayed healing does not necessarily exclude it from participating in a protective mechanism to avoid DNA damage in germ cells and thus safeguard gamete quality. Thus, although it is beyond the scope of the present study, many experiments remain to be performed to get a better idea of the beneficial impact of positive or negative modulation of TGR5 signaling on sperm quantity and quality. We believe that this issue will need to be further evaluated using relevant models in future experiments.

The qualitative impact on spermatozoa is relevant to the continuation of this study, as it is known that *Tgr5* invalidation counteracts testicular defects and degradation of sperm quality, which is induced by a cholic acid‐supplemented diet with a major metabolic impact on multiple generations of individuals.^[^ ^28^ ^]^ This suggests that *Tgr5* plays an important role in sperm quality, and this effect appears to be dependent on the de novo DNA methyltransferase enzyme DNMT3B. A diet supplemented with cholic acid leads to a decrease in *Dnmt3b* expression through TGR5 in germ cells, resulting in a decrease in DNA methylation. Thus, before proposing the modulation of TGR5 in the clinic to safeguard fertility after chemotherapy, it is essential to realize its impact on sperm quality and offspring over several generations in experimental models. This will allow us to avoid defects in sperm quality that could lead to the transgenerational transmission of abnormalities.

The role of TGR5 in response to Bu was evaluated because it is a commonly used molecule in the study of spermatogenesis. The results showed that the invalidation of *Tgr5* in mice minimized the effects of Bu on spermatogenesis, which raised the question of the impact of TGR5 in response to other chemotherapies. Interestingly, the data showed that the impact of the lack of *Tgr5* was also observed using other chemodrugs such as treosulfan, hepsulfam, and cyclophosphamide. In contrast, *Tgr5* invalidation did not alter the response of GC1spg cells to cisplatin. It is interesting to note that cisplatin gives rise to double DNA strand breaks,^[^ ^49^ ^]^ whereas Bu, cyclophosphamide,^[^ ^50^ ^]^ and treosulfan^[^ ^51^ ^]^ generate single strand DNA breaks. These data must help to further define the underlying molecular mechanisms associating the lack of *Tgr5* to protection against chemodrugs.

Next to the protective effect of the lack of TGR5 to minimize the impacts of chemodrugs, the present data show that co‐exposure to Bu and bile acids (endogenous ligands of TGR5) lead to synergic deleterious effects on testicular physiology in vivo. These results were obtained using a cholic acid supplemented diet concomitant with Bu treatment, and they showed that the presence of high levels of bile acids could increase the effectiveness of the treatment by leading to greater and prolonged cell death. This additive effect between Bu and TGR5 activation was confirmed on GC1spg cells. These data support the idea that in the pathophysiological conditions of cancer‐treatment using chemotherapy, it is critical to consider pre‐existent hepatotoxicity. It could lead to a more permanent effect of the chemotherapy on germ cell capacity to recover. As highlighted here, the activation of TGR5 by its agonists led to repression of GLIS2 expression and activation of TP53. This molecular mechanism must explain the crosstalk between TGR5‐GLIS2 and TP53 (Figure 13). These data must be useful to understand the variability of the long‐term impacts of cancer treatments on reproductive capacity among patients.

All the data highlighted the major role of bile acid signaling pathway through TGR5 in germ cell lineage. Interestingly, recent publications demonstrated that the reduction of the richness and/or diversity of intestinal microbiota (IM) in the context of exposure to anticancer drugs (such as Bu), negatively impacted testicular physiology, leading to reduced sperm production.^[^ ^52^ ^]^ Microbiota is involved in the synthesis of secondary BA, which are endogenous ligands of TGR5. The potential association between TGR5 and BA produced by IM^[^ ^53^ ^]^ will have to be studied to better understand how microbiota might impact male fertility following treatments with anticancer drugs.

In conclusion, the present work defines the hitherto unidentified roles of the TGR5 on the physiology of spermatogonial stem cells and progenitor cells. The early germ cell resurgence in Tgr5^–/–^ males after Bu exposure and the prolonged effect of Bu when combined with a TGR5 agonist demonstrate that this pathway is involved in controlling germ cell fate. Specifically, these results identify the key involvement of TGR5‐mediated modulation of GLIS2‐ TP53 pathways in the regulation of stem cell renewal and survival gene expression.

These data are relevant because it has been shown that 69% of patients who underwent chemotherapy (Bu or other chemotherapies such as melphalan) before hematopoietic stem cell transplantation had an increased risk of persistent azoospermia.^[^ ^54^ ^]^ The present work work helps to further understand fertility disorders in the context of cancer treatments and provides new perspectives for developing therapeutic solutions targeting TGR5 for fertility disorders to promote germ cell lineage regeneration. Among chemotherapies, alkylating agents induce long‐term testicular dysfunction and impair fertility.^[^ ^55^ ^]^ However, apart from the cryopreservation of sperm, which is not always possible, little progress has been made to preserve male fertility following cancer treatments, particularly chemotherapy. Therefore, the side effects of cancer treatments on the quality of life, including the maintenance of fertility, is a key issue. It is therefore important to pursue research programs to decipher the underlying molecular mechanisms to improve cancer treatments while minimizing their long‐term side effects, particularly on fertility. This is a public health issue, because with advances in the effectiveness of cancer treatments, patient survival rates have increased,^[^ ^55^, ^56^ ^]^ and it is estimated that 1 in 530 young adults aged 20 to 40 years is a cancer survivor;^[^ ^57^ ^]^ this relative number is expected to increase in the coming decades.

## 4 Experimental Section

### Animals

C57Bl/6J mice were purchased from Charles River Laboratories (L'Arbresle, France). Mice were acclimated at least 2 weeks before experiments. The *Tgr5^−/−^* mice used have been previously described.^[^ ^27^ ^]^ The mice used in this study were maintained in a C57BL/6J background.

To better decipher the role of TGR5 within the germ cell lineage, specific germ cell knock‐out mice were generated using the *Tgr5* floxed mice^[^ ^58^ ^]^ and the model driving the expression of the recombinase‐CRE under the control of the Vasa‐promoter.^[^ ^33^ ^]^ The hTGR5‐T2A‐GFP mouse line was previously described.^[^ ^32^ ^]^

Mice were housed in temperature‐controlled rooms with 12 h light/dark cycles. Mice had ad libitum access to food and water. The refinement is based on the housing and monitoring of the animals as well as the development of protocols that consider the animal welfare. This has been achieved by enriching the cages (cardboard tunnel and mouse houses). The mice were housed in social groups with cage sizes that complied with the legislation according to the number of mice.

12‐week‐old mice were exposed to busulfan, hepsulfam or cyclophosphamide (once, IP, 15mg/kg) and organs were harvested at several time points after from 1 day up to 20 weeks after exposure. The number of animals per group was defined on independent experiments to validate the reproducibility of results.

To minimize the confounders, several independent experiments were done, and groups of animals were not sacrifice in the same order from one experiment to another. These protocols have already been performed in adult mice where no suffering was reported in the time frame of our experiments. However, we have performed daily monitoring of the animals in the first few days following the injection. Wehave monitored food consumption, the possibility of diarrhea. We have checked for signs of suffering activity disorders or physiological changes. No signs of suffering were reported.

This study was conducted in accordance with current regulations and standards approved by Institut National de la Santé et de la Recherche Médicale Animal Care Committee and by the animal care committee (CE2EA Auvergne; protocol CE07‐12 & APAFIS#19626‐2020072312102562v3).

### Fertility Test

Each male was mated at night with a female C57Bl6J (Charles River) for 10 days. Mating was monitored daily for vaginal plugging to determine if mating had occurred. After 17 days of gestation, the mating efficiency was inspected and the number of pups per litter was counted.

### Histology

The testes were collected, fixed in 4% PFA and embedded in paraffin, and 5 µm‐thick sections were prepared and stained with H&E.

### In Vivo TUNEL Analysis

TUNEL experiments were performed, as previously described^[^ ^27^ ^]^ on 5 µm sections of testis fixed in paraformaldehyde 4%. Raw numbers of TUNEL positive cells are given for each vehicle groups in dedicated graphs and then the impact of pharmacological molecules is compared to vehicle groups. For that purpose, vehicle groups of each genotype have been arbitrarily set at 1 and the results are expressed as the relative number of TUNEL positive cells.

### Immunohistochemistry

5 µm sections were mounted on positively charged glass slides (Superfrost plus), deparaffinized, rehydrated, treated for 20 min at 93–98°C in 0.01 M citric buffer‐tween 0.1% (pH 6), rinsed in osmosed water (2 × 5 min), and washed (2 × 5 min) in Phosphate‐buffered saline (PBS). Immunohistochemical studies were conducted according to the manufacturer's recommendations. Slides were then counterstained with Hoechst medium (1 mg/mL) and then mounted on PBS/glycerol (50/50). The antibodies used are reported in Table S4 in the Supporting Information.

For some experiments, raw numbers of positive cells are given for vehicle‐treated groups (control; DMSO 1/1000) in dedicated graphs and then the impact of molecules is analyzed regarding respective vehicle group of each genotype. For that purpose, the vehicle groups of each genotypes have been arbitrarily set at 1.

### Cell Line Approaches

GC1spg cells (ATCC; CRL‐2053) were used as previously described.^[^ ^27^ ^]^ GC1spg Tgr5 knockout cells (GC1spg^KO‐Tgr5^) were generated as following:

### Cell Line Approaches—Generation of Crispr/CAS9 Tgr5 Deficient GC1spg Cells

For the generation of the GC1spg Tgr5 knockout cells, using Crispr/CAS9 approach (see Figure S4 in the Supporting Information). Guides were defined based on the mm9 version of the mouse genome and using the website http://crispor.tefor.net/. The sequences used are for guide‐1: GGCTGCGCAAGTGGCGGTCC and for guide‐2: GCCGGAACCATCAGGGCTAC. Then, the guides were introduced on PX458: pSpCas9(BB)‐2A‐GFP (PX458) (Addgene Plasmid #48138) (Addgene, Watertown, MA, UDA) and PX459: pSpCas9(BB)‐2A‐Puro (PX459) V2.0 (Addgene Plasmid #62988). pSpCas9(BB)‐2A‐Puro (PX459) was a gift from Feng Zhang (Addgene plasmid # 48139; http://n2t.net/addgene:48139; RRID:Addgene_48139).^[^ ^59^ ^]^ Guides were cloned on vectors at the BbsI restriction site.

GC1spg cells were plated on 6‐well plates and transfected, using Jet PEI (Ozyme) with 1 µg of guide 1 in PX458 (expressing pfg) and 2 µg of guide 2 in PX459. 24 h after transfection, using GFP expression, GC1spg cells were sorted by FACS (BD FACSMelody™ Cell Sorter from BD Biosciences) and plated as individual cell in a 96‐well plate in GC1spg conditioned medium (DMEM and 1% SVF). The clones were then validated by sequencing and genotyping. Primer sequences for PCR are giver in Table S5 in the Supporting Information.

For non‐transfected GC1spg or GC1spg^KoTgr5^ cells, cells were plated in 6 well plates. 24 h (24 h) after plating, cells were treated with vehicle (DMSO, 1/1,000), INT777 (25 µM; Sigma‐Aldrich, St. Louis, MO) or busulfan 200 × 10^−6^ m, cyclophosphamide (75 × 10^−6^ m), hepsulfam (3 × 10^−6^ m), treosulfan (10 × 10^−6^ m). For cisplatin (5 × 10^−6^ m), vehicle used was NaCl 0.09% (1/1000). Then, cells were harvested at different time points, and messenger RNA (mRNA) or protein extractions were performed.

### Cell Line Approaches—Transient Transfection—For siRNA Experiments

GC1spg cells were transfected with small interfering RNA (siRNA) using interferin (Ozyme, Saint Quentin Yvelines, France) in six‐well plates (50 000 cells per well). The siRNA directed against *Tgr5* and the control siRNA were previously used (see^[^ ^27^ ^]^). The siRNA directed against *Glis2* was purchased to Darhmacon^TM^ (SMART pool siRNA L‐042208‐01‐0005). The siRNA directed against *Tgr5* or *Glis2*, as well as control siRNA, siCtrl (si Gfp), were transfected at 5 ng per well.

### Cell Line Approaches—Transient Transfection—For Overexpression

GC1spg cells were transfected with Jet‐PEI (Ozyme, Saint Quentin Yvelines, France) in six‐well plates (50,000 cells per well). The plasmid vector of GLIS2 (MR208349, Origene, Leiden, Netherlands) or empty vector were transfected at 200 ng per well.

### Cell Line Approaches—Treatments**—**For Chemodrug Alone Experiments

24 h after the transfection cells were starved for 12 h and treated for 6–24 h later with either DMSO (1/1000) or busulfan (200 × 10^−6^ m), cyclophosphamide (75 × 10^−6^ m), hepsulfam (3 × 10^−6^ m), treosulfan (10 × 10^−6^ m), NaCl 0.9% (1/1000) or cisplatin (5 × 10^−6^ m).

### Cell Line Approaches—Treatments—For TGR5 Agonists ± Bu Experiments

Cells were pretreated with vehicle (DMSO, 1/1000), INT‐777 (12.5 µM ou 25 × 10^−6^ m; Sigma‐Aldrich, St. Louis, MO) or OA (25 × 10^−6^ m; Sigma‐Aldrich, St. Louis, MO), and 24 h later with either DMSO (1/1000) or busulfan (200 × 10^−6^ m).

For each kind of experiments, cells were harvested at 6h, 12h, 24h or 48h later, and immunocytochemistry, mRNA or protein extractions were performed.

### Cell Line Approaches—Luciferase Experiments

cAMP‐response element‐luciferase (cAMP‐RE‐luc) reporter plasmid has been previously described.^[^ ^60^ ^]^ GC1spg^KO‐Tgr5^ cells were transfected with jetPEI (Ozyme) in 24‐well plates. cAMP‐RE‐luc construct (360ng) was transfected together with expression plasmid encoding for mouse TGR5 (40ng). The quantity of DNA was maintained constant by addition of empty pCMX vector to a total amount of 1000ng of DNA per well. Twenty‐four hours after transfection, cells were treated with vehicle, INT‐777 (12.5 or 25 µM), or OA (25 µM). Cells were harvested 24 h later and assayed for luciferase. Luciferase values were normalized to protein quantity.

### Cell Line Approaches—BrdU Incorporation

To define proliferation rate, cells were pretreated with INT‐777 (25 × 10^−6^ m) or vehicle (1/1000) for 24 h and then cells were exposed to vehicle (DMSO 1/1000) or Bu (200 µM) for 24 h. Cells were then incubated with BrdU (10 × 10^−6^ m) for the one last hour. Then cells were washed with PBS1X and fixed with methanol. The detection of BrdU was performed using primary antibody anti‐Bromodeoxyuridine (11170376001, Merck) revealed with specific Alexa488‐coupled secondary antibody.

### Cell Line Approaches—TUNEL

For TUNEL experiments, cells were fixed with PFA 4% on 6 well plates and then the experiments were performed as described above in the immunohistochemistry section. The revelation of the staining was visualized by immunofluorescence.

### Real‐Time RT‐PCR

RNA were isolated using RNAzol. cDNA was synthesized from total RNA with the MMLV and random hexamer primers (Promega, Charbonnières‐les‐Bains, France). The real‐time PCR measurement of individual cDNA was performed using SYBR green dye (Master mix Plus for SYBR Assay, Takara Bio) to measure duplex DNA formation with the Eppendorf‐Realplex system. For each experiment, standard curves were generated with pools of cDNA from cells with different genotypes and/or treatments. The results were analyzed using the ΔΔct method. Primers sequences are reported in Table S5 in the Supporting Information.

### Western Blotting

Proteins were extracted from tissues using RIPA lysis buffer completed with protease inhibitors (Roche Diagnostics, Meylan, France). Antibodies were suspended in Tris‐buffered saline, 0.1% Tween, and 5% BSA. The list of antibodies used is given in the Supporting Information (Table S6, Supporting Information). For some analyses, the quantifications of the protein accumulation were done either against houskeeping gene (GAPDH) or against total stained proteins using stain‐free imaging technology from BIO‐RAD. This allows to obtain quantitative western blot data by normalizing bands to total protein in each lane. On each figure of the present manuscript using stain‐free gels, a representative image of total stained proteins on membrane is given with a highlight made with a crop section at around 50kDa for each experiment.

### RNA‐Seq

The experiment was performed on *GC1spg* cells transfected with siRNA‐*Ctrl* or siRNA‐*Tgr5* and treated with vehicle (1/1000) or busulfan (200 × 10^−6^ m). Starting from RNA, all preparations were made using the IGBMC platform (Illkirch). The mRNA‐seq libraries were sequenced (1 × 50 b).

Reads were mapped onto the mm10 assembly of the mouse genome using Hisat2 v2.1.0^[^ ^61^ ^]^ and the BoWtie2 v2.1.0 aligner.^[^ ^62^ ^]^ Only uniquely aligned reads were retained for further analysis.

Quantification of gene expression was performed using HTSeq v0.5.4p^3[^ ^63^ ^]^ using gene annotations from Ensembl release 77.

Read counts were normalized across libraries with the method proposed by Anders and Huber.^[^ ^64^ ^]^ Comparison between groups was performed using the method proposed previously define^[^ ^65^ ^]^ implemented in the DESeq2 Bioconductor library (DESeq2 v1.0.19). Resulting *p* values were adjusted for multiple testing using the method of Benjamini and Hochberg.^[^ ^66^ ^]^

We generated lists of genes (FC>1.25 and *p* < 0.01) that were differentially expressed after Bu exposure in cells transfected with siCtrl or with siTgr5.

The accession number for the RNA‐seq data reported in this paper is **GSE164734**.

### Magnetic Cell Sorting

Testis cell suspensions from adult Wt or Tgr5^–/–^ males were used for THY1+ spermatogonia cell sorting^[^ ^4^, ^67^ ^]^ using antibodies conjugated to MACS microbeads (CD90.2; Miltenyi Biotec) .

### Statistical Analyses—Preprocessing of Data

For some in vivo experiment raw number of positive cells is given for each vehicle groups in dedicated graphs and then to analyze the impact of pharmacological molecules compared to vehicle groups; vehicle groups of each genotype have been arbitrarily set at 1.

### Statistical Analyses—Data Presentation

All numerical data are represented as mean ± SEM. Significant difference was set at P < 0.05.

Sample size (*n*) for each statistical analysis, Number and type of replicates (e.g., technical replicates, independent experiment, number of mice, and number of independent litters) are reported in the figure legends.

### Statistical Analyses—Statistical Methods

Differences between groups were determined by t‐test or two‐way ANOVA. Multiple comparisons were made with Holm‐Sidak's test. Spearman correlation test was used for some analysis.

Statistical analyses were done using Sigmastat3 software.

## Conflict of Interest

The authors declare no conflict of interest.

## Author Contributions

All authors agree with this publication. T.L. and V.D.H. performed and designed the experiments. T.L., H.H., M.M., G.M., d.H.A., D.‐S.C., R.Y., S.J.‐P., P.A., and S.K. performed the experiments. T.L., H.H., R.Y., B.C., and V.D.H. participated to write the paper.

## Supporting information

Supporting Information

Supporting Information

## Data Availability

The data that support the findings of this study are available from the corresponding author upon reasonable request.
