# Correction to “Identification of a Crosstalk Among TGR5, GLIS2, and TP53 Signaling Pathways in the Control of Undifferentiated Germ Cell Homeostasis and Chemoresistance”

**DOI:** 10.1002/advs.74330

**Published:** 2026-02-12

Thirouard L, Holota H, Monrose M, Garcia M, de Haze A, Damon‐Soubeyrand C, Renaud Y, Saru JP, Perino A, Schoonjans K, Beaudoin C, Volle DH. “Identification of a Crosstalk Among TGR5, GLIS2, and TP53 Signaling Pathways in the Control of Undifferentiated Germ Cell Homeostasis and Chemoresistance.” Adv Sci (Weinh). 2022 Jun;9(17):e2200626. doi: 10.1002/advs.202200626. Epub 2022 Apr 18. PMID: 35435331.

In the published article, Figure 12D contains an inadvertent error that occurred during the final stages of manuscript preparation. Figure 12D has an incorrect image that is a duplication of panel 12B, which shows GC1spg cells pre‐exposed for 24 h to INT‐777 and then to Bu for 48 h and stained for TUNEL, when it should be an image of PLZF staining of Wt male testes treated with Bu. We retrieved the original data submitted to Advanced Science in 2021, and the corrected Figure 12D is presented as follows.

**Corrected Figure 12D**


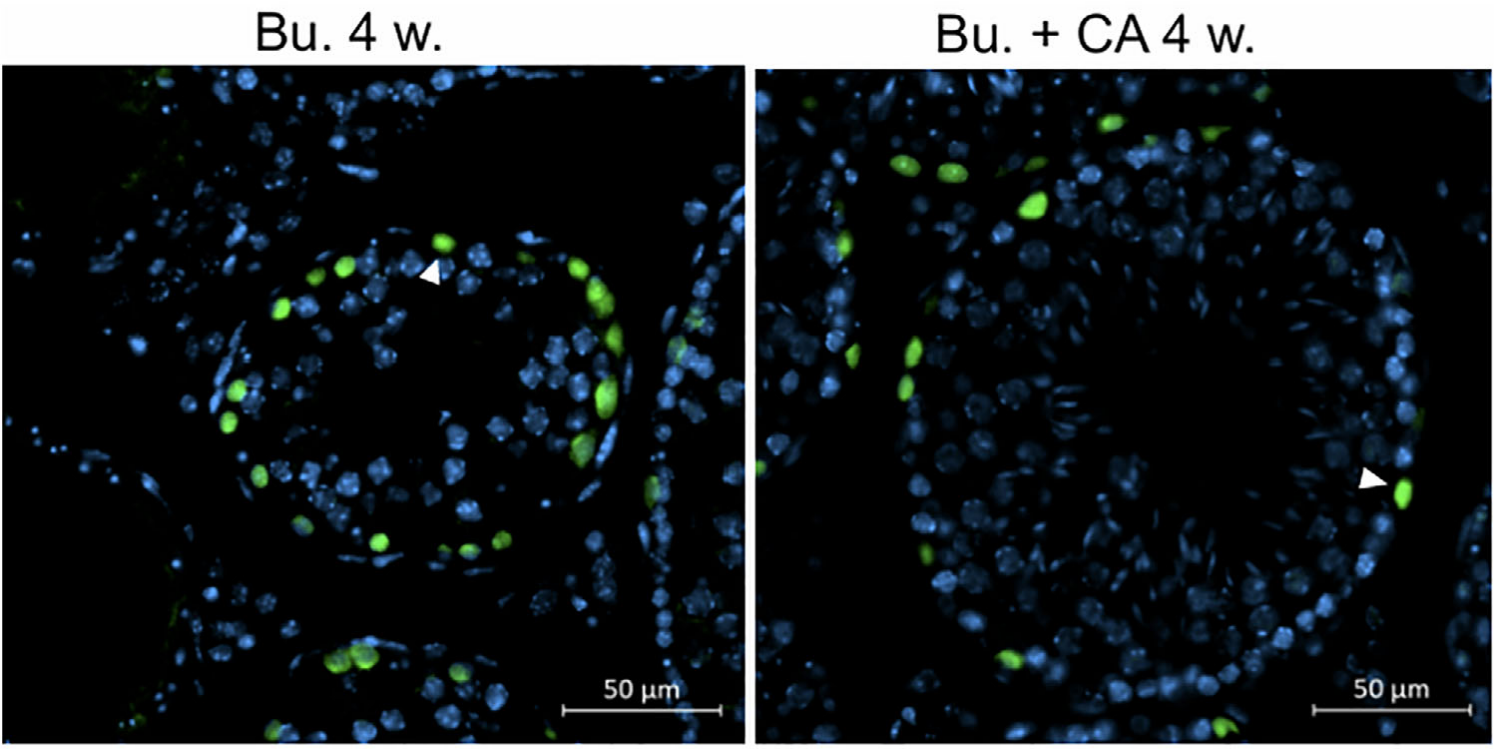


In the published article, Figure 12F contains an inadvertent error that occurred during the final stages of manuscript preparation. Figure 12F has an incorrect image that is a duplication of panel 12E, which shows an immunofluorescence‐stained testis section for acetylated histone H4, a marker for spermatids, when it should be an image of TUNEL staining of mouse testicles exposed to busulfan and CA for 8 weeks. We retrieved the original data submitted to Advanced Science in 2021, and the corrected Figure 12F is shown as follows.

**Corrected Figure 12F**


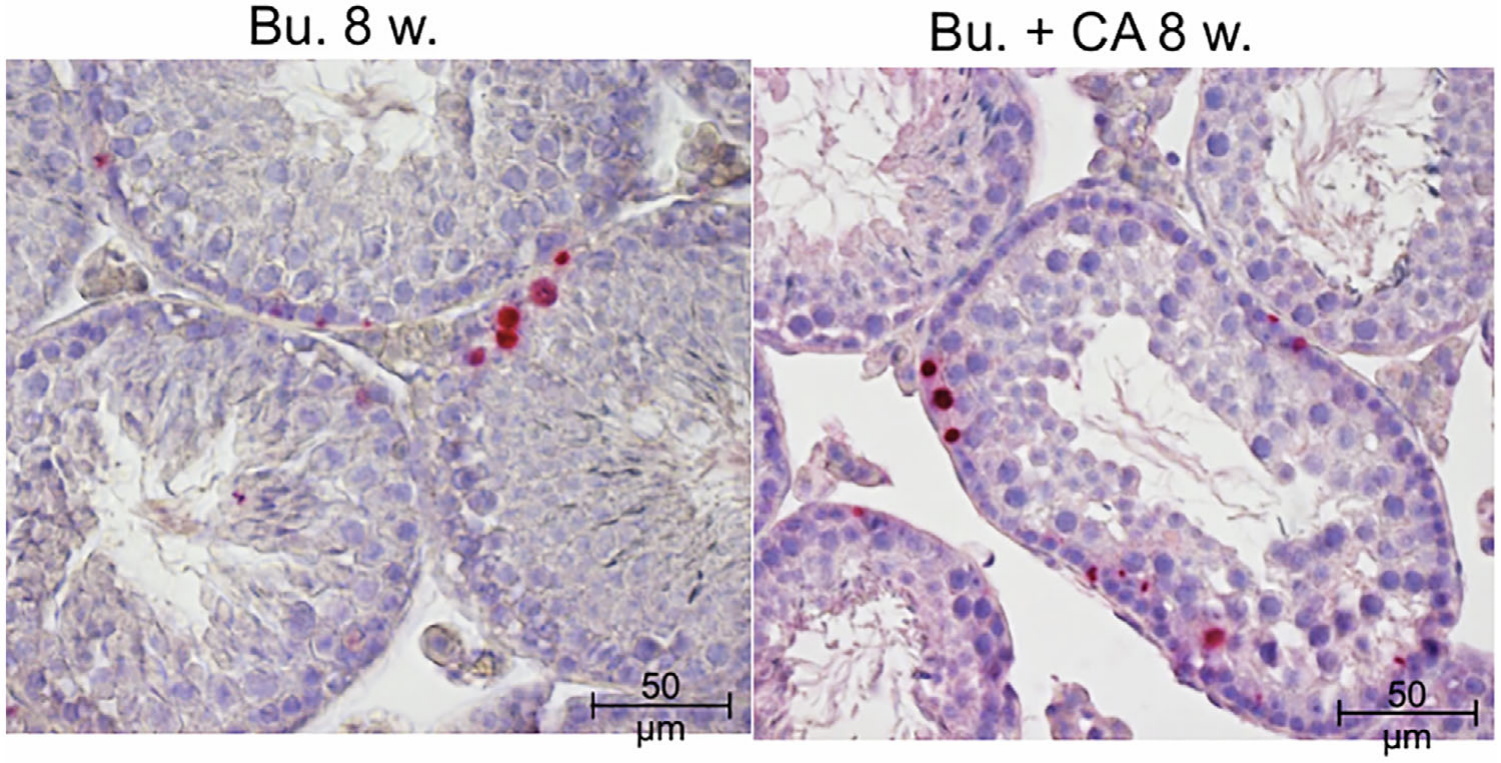


The authors apologize for these errors.

