# Supplementary material for: Identification of a Crosstalk among TGR5, GLIS2, and TP53 Signaling Pathways in the Control of Undifferentiated Germ Cell Homeostasis and Chemoresistance
Source: Adv Sci (Weinh). 2022 Apr 18;9(17):2200626. doi: 10.1002/advs.202200626 (PMC9189661; doi:10.1002/advs.202200626)
Supplement: Supplementary file 1 — Supporting Information [file ADVS-9-2200626-s001.pdf]

ADVANCED SCIENCE
Open Access

# Supporting Information

for *Adv. Sci.*, DOI 10.1002/advs.202200626

Identification of a Crosstalk among TGR5, GLIS2, and TP53 Signaling Pathways in the Control of Undifferentiated Germ Cell Homeostasis and Chemoresistance

*Laura Thirouard, Hélène Holota, Mélusine Monrose, Manon Garcia, Angélique de Haze, Christelle Damon-Soubeyrand, Yoan Renaud, Jean-Paul Saru, Alessia Perino, Kristina Schoonjans, Claude Beaudoin and David H. Volle**

**Table S1a**: List of genes affected by Bu versus veh. in siCtrl-transfected cells.

**Table S1a**: List of genes affected by Bu versus veh. in siCtrl-transfected cells.

| | | | | | | |
|---|---|---|---|---|---|---|
| Scd1 | Gm11400 | Alpl | Serpinf1 | Atp6v0c | Gm44667 | Gm42876 |
| Il1rn | Slc2a4rg-ps | Ing4 | Hnrnph1 | Tnik | Gm45632 | Gm43327 |
| Naaladl2 | Gm12940 | Fgf21 | Fnbp4 | Sorcs2 | Gm45250 | Hk2 |
| Adgrl3 | Gdap10 | L1cam | Glis2 | Plxna4 | AC160637.1 | Slc4a3 |
| Sox5 | 2610037D0 | Adgrg1 | Ahr | St8sia1 | Gm47260 | Esr1 |
| Prkg1 | Gm42664 | Amt | Clk4 | Plxna3 | Gm49284 | Itgb3 |
| Immp2l | Gm43128 | Catsper2 | Limk2 | Pygm | Gm49204 | Cygb |
| Angptl7 | Gm35339 | Adora1 | Rapgef3 | Unc13a | AC105358.1 | Nxnl2 |
| Ung | 9930017N2 | Lrrn4 | Clcn2 | Tmtc2 | Lama4 | Gfra1 |
| Ptprm | AC154232.2 | Cfap46 | Gtpbp2 | Adamts2 | Klhl29 | Itgb8 |
| Gpc6 | Asic1 | 4732471J01 | Atat1 | Fgf1 | Slc17a9 | Nkain4 |
| Gas7 | Agap2 | Izumo4 | Arhgef9 | Arl5c | Gabbr1 | Mmp16 |
| Mgp | Zfr2 | Fam57b | Tmeff2 | Atp5l | Apba1 | Nup210 |
| Kifc2 | Zcchc7 | Snora2b | Rassf8 | Zdhhc1 | Plce1 | Lctl |
| Col20a1 | Pitpnm3 | Celsr2 | Fgfr2 | Cdh18 | Nrp2 | Nktr |
| Cacna1g | Gapdh | 4933439C1 | Ankrd10 | Arglu1 | sept-02 | Igdcc4 |
| Vdr | Trabd2b | Wt1os | 6430548M0 | Tet2 | Dclk2 | Ank2 |
| Cd200 | Snhg17 | 1700020D0 | Hyou1 | Adamts3 | Per3 | Wnk4 |
| Fa2h | Malat1 | Gm49396 | Gmip | 2900005J15 | Pcsk6 | Ints6l |
| Flrt1 | Pdzd2 | Muc16 | Nod1 | Adgrd1 | Tenm3 | Arhgap26 |
| Kcnq3 | Podxl | Gm20219 | Gcnt1 | Rnf182 | Ulk3 | Spred3 |
| Megf6 | Smpd3 | AC122335.1 | Spns2 | Tenm4 | Loxl1 | Tgm2 |
| Zfp773 | mt-Nd6 | AC162302.2 | Atp1b2 | Lingo1 | Pknox2 | Ccr7 |
| 1700001L0 | Ckmt1 | Rrnad1 | Ppp1r3b | Pard3b | Csgalnact1 | Camk4 |
| Sbk3 | Ttc22 | Anxa9 | Prdm9 | Slit3 | Gtdc1 | Ak5 |
| AI480526 | Med12l | P4ha2 | Haghl | Hmcn1 | Stox2 | Ncam1 |
| Gm37893 | Gm28151 | Col6a2 | Msln | Dnm3os | Kazn | Plekhg5 |
| Adgrb2 | Rnf112 | Nid2 | Rab44 | Airn | Atxn1 | Six5 |
| Spag1 | Slc22a17 | 6030458C1 | Tia1 | Prkcg | Sox6 | Kif21b |
| Fzd4 | Usp53 | Ly6f | Emc1 | B930095G1 | D930048N1 | Nemp2 |
| Il33 | Abca9 | Paxbp1 | Sema5a | Wipf3 | Gas1 | Eml6 |
| Rspo1 | Fbxl7 | Krt7 | Adamtsl1 | Gm15675 | Slc4a4 | Ttc41 |
| Fras1 | Snx32 | Wfdc1 | 3110039I08 | 4933407K1 | Maml3 | Magi1 |
| Csdc2 | 5430430B1 | Vegfa | Gm21781 | 2900089D1 | Zfp467 | Gpr68 |
| Kcnb1 | Susd2 | Ikzf2 | Gm20342 | Neat1 | Olfr1372-ps | Gli2 |
| Fbln5 | Rhbdl3 | Ccnt2 | Gm20045 | Gm29666 | Ube2i | Nckap5 |
| Adamts20 | Lcor | Sema6d | Gm44200 | Gm28941 | Cacnb1 | Zfp579 |
| Extl1 | Slco5a1 | Ccnl1 | Xylt1 | Gm38057 | Pabpn1 | Xkr4 |
| Tmem108 | Rnft2 | Plxnb3 | Col4a4 | Gm36936 | Npff | Nav2 |
| Col8a2 | Plekha7 | Car12 | Alcam | Gm36989 | Adamts10 | Tln2 |
| Mir22hg | Fgf18 | Zfc3h1 | Sorcs1 | 6430511E1 | Gpc2 | Grk4 |
| Zfp950 | Pisd-ps1 | Sv2a | Dhrs3 | A930004J17 | Gabre | Nebl |
| Slc1a2 | Dennd6b | Gjb3 | Cfp | Gm38253 | Igsf9 | Slc8a1 |
| Tnfrsf25 | Ager | Synpo | Hoxc8 | Gm38372 | Fam227a | Ppp4r1l-ps |
| Slc27a3 | Rac3 | Il17re | Apoe | Gm37206 | Kcp | Syngap1 |
| Prss12 | Adcy1 | Zbtb37 | Aqp1 | Gm38248 | Actg1 | Msi2 |
| Ttll3 | Dnase1l2 | Sv2c | 1700109H0 | Gm42979 | Grid2 | Prob1 |
| Leng8 | Mapk8ip3 | Dock8 | Matn4 | Gm43759 | Tgfbr3l | Mob3b |
| Atp8a1 | Nudt8 | Ggcx | Hoxc5 | Gm43300 | Pla2g4b | Carns1 |
| Ankrd16 | Lrp2 | Dleu2 | Pcdhgc4 | Gm43059 | Gm37261 | Nrbp2 |

| | | | | | | |
|---|---|---|---|---|---|---|
| Il18bp | Pnisr | Zc3h11a | Sfi1 | Gm42549 | Gm37963 | Smim1 |
| Spaca6 | Pdzk1ip1 | 9330188P0: | Slc25a27 | Gm44597 | Gm42913 | C430049B03Rik |
| D430040D2 | Pi16 | Gm16574 | Eno2 | Dennd4b | Slc16a3 | Rere |
| Gm17491 | Rgs11 | Gm17024 | Pdk1 | Hoxd11 | Mbd6 | Spsb1 |
| 5033428I22 | Mamdc4 | Mir5125 | Runx1t1 | Cilp2 | Pdia4 | Zfhx2 |
| Slc1a5 | Grin1 | Gm26546 | Dqx1 | Insig1 | Clk1 | Cables1 |
| Celf2 | Adam33 | Gm26786 | Taz | Trim39 | Pfkfb2 | Tspyl2 |
| Ppp1cc | Mfsd2a | Gm26890 | Slc9a5 | Col27a1 | Atp1b1 | Cacna2d4 |
| Chrd | Tekt2 | Gm16754 | Dbndd2 | Ankrd34a | Rrbp1 | Bicdl1 |
| Nfib | Morn1 | Gm28043 | Tnfsfm13 | Sox12 | Fam210b | Acacb |
| Appl2 | Ccnl2 | Gm28119 | Pfkl | Trpm3 | Mecom | Msantd2 |
| Trib2 | Mapkapk5 | Gm28730 | Lpin1 | Zfp608 | Olfml3 | Zmym6 |
| Epas1 | Tbx6 | Gm37968 | Mrc2 | 2700081O1! | Pdgfc | 4632404H12Rik |
| Col5a1 | Gdpd3 | Gm37795 | Vash1 | Msi1 | Bnc2 | Safb2 |
| Kcnab1 | Cox6a2 | Gm37154 | Hhipl1 | Ankrd24 | Podn | Trim46 |
| Itpr1 | Cngb1 | Gm38319 | Fam193b | Ddx17 | Slc2a1 | Upk3b |
| Psd3 | Ppp1r9a | Gm37309 | Sla | Zfp607b | Mthfr | Ccdc84 |
| Fchsd2 | Inha | Gm37274 | Chkb | Rnf213 | Clcn6 | Als2cl |
| Aff2 | Ssc5d | Gm38220 | Thpo | Mn1 | Gm996 | Dock5 |
| Prr5l | Rsrp1 | Gm38020 | Lmbr1l | Kcnip3 | Col1a2 | Krt14 |
| Arhgap39 | Pkhd1l1 | Gm38077 | Amhr2 | Tnfsf13os | Adamts9 | Onecut2 |
| Evpl | Shank1 | Gm43858 | Satb1 | Gm38394 | Pianp | Slc25a23 |
| Acot11 | Prelp | Gm43511 | Map4k2 | Ptprv | Rhpn2 | Vat1l |
| Rapgefl1 | Fam129c | Gm43413 | Pcyt2 | Xndc1 | Abcd1 | Khnyn |
| Mllt6 | Exoc3l | Gm43445 | Cfh | Pcdhga5 | Col4a2 | Kmt2d |
| Nos1ap | Casc1 | Gm43071 | Ttc14 | Gmpr | Plpp5 | Hoxb3 |
| Col16a1 | Sh2d5 | Gm42603 | Epb41l4b | Col1a1 | Slc27a1 | Asap2 |
| Plekha6 | Smtnl2 | Gm36535 | Ak4 | Grik5 | Fam46a | Plxnb1 |
| Igsf3 | Rimkla | Gm43080 | Mmp17 | Crtc1 | Man2c1 | Usp13 |
| Nrep | Krt9 | Gm43513 | Gigyf1 | Gys1 | Adamts7 | Kmt5c |
| Rnf150 | Mpp3 | Gm43328 | Dmpk | Stx1a | Zic1 | Rpl3 |
| Ldlrad3 | Lgals4 | Gm43693 | Myo7a | Zmiz1 | Nphp3 | Nr6a1 |
| Mical3 | Cpa1 | Gm42820 | Kifc3 | Vav2 | Mst1r | Tacc1 |
| Phactr1 | 9130017K1: | Gm43029 | Jak3 | Pou6f1 | Abcc10 | Sptbn2 |
| Klf12 | Rnf207 | Gm43481 | Tnxb | Adgrl1 | Lss | Clk2 |
| D030028A0: | Clasrp | Gm43747 | Sfxn5 | Lama5 | Szt2 | Rbm47 |
| P2rx3 | Vmn1r13 | Gm42572 | Ppargc1b | Aldoc | Stard9 | Apcdd1 |
| Il16 | Ankrd23 | Gm42732 | Ypel4 | Kdm6b | Zfp446 | Wdr90 |
| Syn3 | Selenbp1 | Gm44243 | Tspoap1 | Rmnd1 | Ogt | Ptar1 |
| Gm37376 | Acrbp | Gm45838 | Adrb1 | Tns3 | Slc25a37 | 6330403L08Rik |
| Papln | 4930518I15 | Gm45221 | Nat14 | Ern1 | Sirt6 | Bnip3 |
| Aldh1l2 | Pdzd7 | Gm9856 | Nfkbiz | Slc9a3r1 | Tet3 | Rab10os |
| Mylk | Gm2420 | Gm47963 | Crb2 | Usp43 | Pcyt1b | Gm2415 |
| Fam151a | Gm9164 | Gm47113 | Zfp57 | Zfp36l1 | Ror1 | Gm7694 |
| Fmnl1 | Gm5466 | Gm47483 | Ppfibp2 | Srsf5 | Ntng2 | Npy4r |
| Per2 | Gm15344 | Gm33869 | Arhgap33 | Abcd4 | Aldh1b1 | Ces2e |
| Gm38391 | Gm15337 | Gm47583 | Mycn | Ptch1 | Prpf39 | Ly6c1 |
| Srpk3 | Firre | Gm47585 | Bcl9 | Pde4d | Sugp2 | Dusp4 |
| Rpl10 | Gm15513 | Gm47798 | Angptl6 | Hmbox1 | Stag3 | Ly6a |
| Cdh23 | Chn1os3 | Gm49223 | Whrn | Wnt5a | Uvssa | Ccl9 |
| Kpna2 | BC065397 | AC133488.1 | Lrp1 | Sdf2l1 | Ahdc1 | Serpinb9b |
| Cyp46a1 | Gm11266 | Col6a1 | Clcf1 | Map3k8 | Kmt2c | Aldh3a1 |

| | | | | | | |
|---|---|---|---|---|---|---|
| Naprt | C030037D0! | Cldn15 | Crocc | Tcf7l2 | Setd1b | Ankrd1 |
| Pgc | Gm23547 | Hmgn2 | Mill2 | Lrrc45 | Higd1a | Dcxr |
| Cd14 | Rpl37 | Kif18a | Gmnn | Iqgap3 | Fanci | Mylpf |
| Emp1 | S100a13 | Nusap1 | Syngr3 | Smc2 | Neil3 | Ltf |
| 9530053A0 | Rps8 | Bub1 | Oit3 | Zfp37 | Exo1 | Mslnl |
| Gna14 | Gm8730 | Aurka | Abca1 | Ndc1 | Bub1b | Gm11223 |
| Aqp3 | Rpl23 | Fam83d | Gadd45b | Rad54l | Pclaf | Gpr85 |
| S100a7a | Fndc10 | Ccna2 | Brca1 | Stil | Siah1b | Slc29a4 |
| Gsto1 | Mt2 | Depdc1a | Mybl2 | Cdca8 | Kif14 | Defb42 |
| Lamb3 | Slc37a2 | Kif2c | Chtf18 | Cenpa | Clspn | 1700003M07Rik |
| Fgf13 | Svop | Abcb1b | Fbxo5 | Ereg | Tuba1c | Gm17655 |
| Plet1 | Mab21l3 | Aass | Sgk1 | Chek2 | Cdc25c | 9430037O13Rik |
| Acta2 | Sec14l5 | Tuba8 | Mdm2 | Mad2l1 | Cenph | Gria3 |
| Anpep | Eda2r | Ddias | Nfyb | Rad18 | Ticrr | Def6 |
| Cd24a | Gen1 | Cenpu | Txnrd1 | Rad51ap1 | Mis18bp1 | Tbc1d8 |
| Nipal1 | Spag5 | Robo3 | Pttg1 | Blm | Fancb | Hspb1 |
| 1110038B1 | Kifc5b | Mcam | Tubd1 | Synm | Sephs2 | Tek |
| Gdf15 | Polq | BC030867 | Ppm1d | Aen | Syt12 | Cct4 |
| Adgrf4 | Arhgap11a | Gna15 | Rrm2 | Arl6ip1 | Haspin | Snrpb2 |
| Rps20 | Cdca2 | Lrr1 | Top2a | Kif22 | Hyls1 | Hyal1 |
| Rps27l | Wnt4 | Ska1 | Vcan | Plk1 | Ercc6l | Rdm1 |
| Eva1c | Tigit | Tubb4b | Cenpk | Mki67 | Tnfaip6 | Mrpl32 |
| Ecscr | Cdc6 | Dlgap5 | Ska3 | Cenpi | Hmgb2 | Hebp2 |
| Rpl39 | Pimreg | Cdkn3 | Esd | Mak16 | Fam72a | Nudcd2 |
| Rps10 | Ccnb2 | Ckap2 | Pbk | Gins3 | Ier5 | Jpt1 |
| Col14a1 | Ube2c | Zfp365 | Gtse1 | Cenpn | Espl1 | Dnaaf2 |
| Duoxa1 | Spc25 | Ttk | Mis18a | Mmp15 | Tubb2a | Efcab11 |
| Cyp2b10 | Nectin4 | Prc1 | Racgap1 | Mns1 | Zfp958 | Dcaf4 |
| Prr7 | Kif11 | Eme1 | Cdkn1a | Kif23 | Nsl1 | Trip13 |
| Mettl7a3 | Ncapg | Atf7ip2 | Poc1a | Gclc | Tubb4a | Fst |
| Alox5ap | Birc5 | Ccnb1 | Cdca3 | Zwilch | Bub3 | Dnajc9 |
| Tubb3 | Cdk1 | Tubb2b | Dusp1 | Traip | Spdl1 | Diaph3 |
| 4930471E1! | Mtfr2 | Cenpe | Incenp | Srxn1 | Fv1 | Eif3e |
| Dram1 | Ccng1 | Frat2 | Fam111a | C330027C0! | Ccnf | Ly6e |
| Dglucy | Hmmr | Ckap2l | Cep55 | Ttf2 | Ang | Rfc4 |
| Nme4 | Prr11 | Kif18b | Plk4 | Aspm | Tuba1a | Pros1 |
| Cyp2c55 | Aurkb | Spn | Bard1 | Fancd2 | Spc24 | Cbr3 |
| Spint1 | Cenpp | Nrg1 | Tuba4a | Kif4 | Slc4a11 | Thbs2 |
| Rps3 | Depdc1b | Oip5 | Ube2t | Trim59 | Gas2l3 | Pigf |
| Phlda3 | Esco2 | Rprm | Cenpf | Brip1 | Cenpw | Rps14 |
| Ctla2a | Shcbp1 | Aunip | Suv39h2 | Smc4 | Kifc1 | Fas |
| Cd109 | Cenpq | 2010110K1 | Nuf2 | Ncaph | Haus3 | Crcp |
| Gm6030 | Sgo1 | Gm36401 | Cenpl | Nup37 | 2810408I11 | Stk17b |
| Ethe1 | Ndc80 | Cdc45 | Sapcd2 | Parpbp | 9230114K1 | Irf6 |
| Rpl13a | Pmaip1 | S100a3 | Depdc7 | Thyn1 | 2700099C1 | Phospho2 |
| Gm8199 | Cdca5 | Tubb6 | Rad51 | Melk | 2610044O1 | Eif3m |
| Fam26e | Kif20b | Foxm1 | Knl1 | Kif15 | AC129328.1 | Fabp5 |
| Perp | Fosl1 | Tubb5 | Cdc25b | Anln | Slc2a9 | Exosc9 |
| Rspo3 | Sgo2a | Dbf4 | Knstrn | Grhl3 | Slfn3 | Tlr2 |
| Cox7a2l | Serpinb8 | Cse1l | Mcm8 | Tacc3 | Ms4a10 | Polr1e |
| Mmp24 | Nek2 | Kif20a | Tpx2 | Dtl | Pnliprp2 | Prdx1 |
| Rps3a1 | Mcm10 | Nqo1 | Ect2 | Rtkn2 | Casq2 | Stmn1 |

| | | | | | | |
|---|---|---|---|---|---|---|
| Glipr2 | Mastl | Asf1b | Gpsm2 | 1700066M2 | Nppb | Rcc1 |
| Slc19a2 | 1700007K1: | Cdc20 | Sass6 | Sccpdh | Cgref1 | Lzic |
| Triap1 | Zfp850 | | | | | |
| Mphosph10 | Gm20667 | | | | | |
| Ap1s2 | Zfp960 | | | | | |
| Vps36 | | | | | | |
| Mfap3l | | | | | | |
| Tbc1d9 | | | | | | |
| Ccsap | | | | | | |
| Abhd10 | | | | | | |
| Reep4 | | | | | | |
| Tmem5 | | | | | | |
| Celf5 | | | | | | |
| Trim13 | | | | | | |
| Calm2 | | | | | | |
| Cib2 | | | | | | |
| Rpf2 | | | | | | |
| Ephx1 | | | | | | |
| Myl6b | | | | | | |
| Rbm48 | | | | | | |
| Pitpnc1 | | | | | | |
| St8sia4 | | | | | | |
| Gfer | | | | | | |
| Rpl7 | | | | | | |
| Tnfaip8l1 | | | | | | |
| Cxcr4 | | | | | | |
| Dlk2 | | | | | | |
| Mlf1 | | | | | | |
| Fam212b | | | | | | |
| Fam241a | | | | | | |
| Ubald2 | | | | | | |
| Insl6 | | | | | | |
| Nat2 | | | | | | |
| Zfp472 | | | | | | |
| Glipr1 | | | | | | |
| Zfp729b | | | | | | |
| Ube2s | | | | | | |
| Ei24 | | | | | | |
| Arhgap22 | | | | | | |
| Vrk2 | | | | | | |
| Syt11 | | | | | | |
| Zfp874a | | | | | | |
| Tmem19 | | | | | | |
| Nanos1 | | | | | | |
| Slfn2 | | | | | | |
| 5430403G16Rik | | | | | | |
| Osgin1 | | | | | | |
| Smim26 | | | | | | |
| Arrdc3 | | | | | | |
| Rad54b | | | | | | |
| Zfp429 | | | | | | |
| Gm14681 | | | | | | |

**Table S1a-1**: List of genes decreased by Bu versus veh. in siCtrl-transfected cells.

Table S1a-1: List of genes decreased by Bu versus veh. in siCtrl-transfected cells.

| | | | | | | |
|---|---|---|---|---|---|---|
| Scd1 | Gm11400 | Alpl | Serpinf1 | Atp6v0c | Gm44667 | Gm42876 |
| Il1rn | Slc2a4rg-ps | Ing4 | Hnrnph1 | Tnik | Gm45632 | Gm43327 |
| Naaladl2 | Gm12940 | Fgf21 | Fnbp4 | Sorcs2 | Gm45250 | Hk2 |
| Adgrl3 | Gdap10 | L1cam | Glis2 | Plxna4 | AC160637.1 | Slc4a3 |
| Sox5 | 2610037D0: | Adgrg1 | Ahr | St8sia1 | Gm47260 | Esr1 |
| Prkg1 | Gm42664 | Amt | Clk4 | Plxna3 | Gm49284 | Itgb3 |
| Immp2l | Gm43128 | Catsper2 | Limk2 | Pygm | Gm49204 | Cygb |
| Angptl7 | Gm35339 | Adora1 | Rapgef3 | Unc13a | AC105358.1 | Nxnl2 |
| Ung | 9930017N2: | Lrrn4 | Clcn2 | Tmtc2 | Lama4 | Gfra1 |
| Ptprm | AC154232.2 | Cfap46 | Gtpbp2 | Adamts2 | Klhl29 | Itgb8 |
| Gpc6 | Asic1 | 4732471J01 | Atat1 | Fgf1 | Slc17a9 | Nkain4 |
| Gas7 | Agap2 | Izumo4 | Arhgef9 | Arl5c | Gabbr1 | Mmp16 |
| Mgp | Zfr2 | Fam57b | Tmeff2 | Atp5l | Apba1 | Nup210 |
| Kifc2 | Zcchc7 | Snora2b | Rassf8 | Zdhhc1 | Plce1 | Lctl |
| Col20a1 | Pitpnm3 | Celsr2 | Fgfr2 | Cdh18 | Nrp2 | Nktr |
| Cacna1g | Gapdh | 4933439C1( | Ankrd10 | Arglu1 | sept-02 | Igdcc4 |
| Vdr | Trabd2b | Wt1os | 6430548M0 | Tet2 | Dclk2 | Ank2 |
| Cd200 | Snhg17 | 1700020D0: | Hyou1 | Adamts3 | Per3 | Wnk4 |
| Fa2h | Malat1 | Gm49396 | Gmip | 2900005J15 | Pcsk6 | Ints6l |
| Flrt1 | Pdzd2 | Muc16 | Nod1 | Adgrd1 | Tenm3 | Arhgap26 |
| Kcnq3 | Podxl | Gm20219 | Gcnt1 | Rnf182 | Ulk3 | Spred3 |
| Megf6 | Smpd3 | AC122335.1 | Spns2 | Tenm4 | Loxl1 | Tgm2 |
| Zfp773 | mt-Nd6 | AC162302.2 | Atp1b2 | Lingo1 | Pknox2 | Ccr7 |
| 1700001L0! | Ckmt1 | Rrnad1 | Ppp1r3b | Pard3b | Csgalnact1 | Camk4 |
| Sbk3 | Ttc22 | Anxa9 | Prdm9 | Slit3 | Gtdc1 | Ak5 |
| AI480526 | Med12l | P4ha2 | Haghl | Hmcn1 | Stox2 | Ncam1 |
| Gm37893 | Gm28151 | Col6a2 | Msln | Dnm3os | Kazn | Plekhg5 |
| Adgrb2 | Rnf112 | Nid2 | Rab44 | Airn | Atxn1 | Six5 |
| Spag1 | Slc22a17 | 6030458C1: | Tia1 | Prkcg | Sox6 | Kif21b |
| Fzd4 | Usp53 | Ly6f | Emc1 | B930095G1! | D930048N1 | Nemp2 |
| Il33 | Abca9 | Paxbp1 | Sema5a | Wipf3 | Gas1 | Eml6 |
| Rspo1 | Fbxl7 | Krt7 | Adamtsl1 | Gm15675 | Slc4a4 | Ttc41 |
| Fras1 | Snx32 | Wfdc1 | 3110039I08 | 4933407K1: | Maml3 | Magi1 |
| Csdc2 | 5430430B1‹ | Vegfa | Gm21781 | 2900089D1 | Zfp467 | Gpr68 |
| Kcnb1 | Susd2 | Ikzf2 | Gm20342 | Neat1 | Olfr1372-ps: | Gli2 |
| Fbln5 | Rhbdl3 | Ccnt2 | Gm20045 | Gm29666 | Ube2i | Nckap5 |
| Adamts20 | Lcor | Sema6d | Gm44200 | Gm28941 | Cacnb1 | Zfp579 |
| Extl1 | Slco5a1 | Ccnl1 | Xylt1 | Gm38057 | Pabpn1 | Xkr4 |
| Tmem108 | Rnft2 | Plxnb3 | Col4a4 | Gm36936 | Npff | Nav2 |
| Col8a2 | Plekha7 | Car12 | Alcam | Gm36989 | Adamts10 | Tln2 |
| Mir22hg | Fgf18 | Zfc3h1 | Sorcs1 | 6430511E1! | Gpc2 | Grk4 |
| Zfp950 | Pisd-ps1 | Sv2a | Dhrs3 | A930004J17 | Gabre | Nebl |
| Slc1a2 | Dennd6b | Gjb3 | Cfp | Gm38253 | Igsf9 | Slc8a1 |
| Tnfrsf25 | Ager | Synpo | Hoxc8 | Gm38372 | Fam227a | Ppp4r1l-ps |
| Slc27a3 | Rac3 | Il17re | Apoe | Gm37206 | Kcp | Syngap1 |
| Prss12 | Adcy1 | Zbtb37 | Aqp1 | Gm38248 | Actg1 | Msi2 |
| Ttll3 | Dnase1l2 | Sv2c | 1700109H0: | Gm42979 | Grid2 | Prob1 |
| Leng8 | Mapk8ip3 | Dock8 | Matn4 | Gm43759 | Tgfbr3l | Mob3b |
| Atp8a1 | Nudt8 | Ggcx | Hoxc5 | Gm43300 | Pla2g4b | Carns1 |
| Ankrd16 | Lrp2 | Dleu2 | Pcdhgc4 | Gm43059 | Gm37261 | Nrbp2 |
| Il18bp | Pnisr | Zc3h11a | Sfi1 | Gm42549 | Gm37963 | Smim1 |
| Spaca6 | Pdzk1ip1 | 9330188P0: | Slc25a27 | Gm44597 | Gm42913 | C430049B03Rik |

Table S1a-1: List of genes decreased by Bu versus veh. in siCtrl-transfected cells.

| | | | | | | |
|---|---|---|---|---|---|---|
| D430040D2 | Pi16 | Gm16574 | Eno2 | Dennd4b | Slc16a3 | Rere |
| Gm17491 | Rgs11 | Gm17024 | Pdk1 | Hoxd11 | Mbd6 | Spsb1 |
| 5033428I22 | Mamdc4 | Mir5125 | Runx1t1 | Cilp2 | Pdia4 | Zfhx2 |
| Slc1a5 | Grin1 | Gm26546 | Dqx1 | Insig1 | Clk1 | Cables1 |
| Celf2 | Adam33 | Gm26786 | Taz | Trim39 | Pfkfb2 | Tspyl2 |
| Ppp1cc | Mfsd2a | Gm26890 | Slc9a5 | Col27a1 | Atp1b1 | Cacna2d4 |
| Chrd | Tekt2 | Gm16754 | Dbndd2 | Ankrd34a | Rrbp1 | Bicdl1 |
| Nfib | Morn1 | Gm28043 | Tnfsfm13 | Sox12 | Fam210b | Acacb |
| Appl2 | Ccnl2 | Gm28119 | Pfkl | Trpm3 | Mecom | Msantd2 |
| Trib2 | Mapkapk5 | Gm28730 | Lpin1 | Zfp608 | Olfml3 | Zmym6 |
| Epas1 | Tbx6 | Gm37968 | Mrc2 | 2700081O1 | Pdgfc | 4632404H12Rik |
| Col5a1 | Gdpd3 | Gm37795 | Vash1 | Msi1 | Bnc2 | Safb2 |
| Kcnab1 | Cox6a2 | Gm37154 | Hhipl1 | Ankrd24 | Podn | Trim46 |
| Itpr1 | Cngb1 | Gm38319 | Fam193b | Ddx17 | Slc2a1 | Upk3b |
| Psd3 | Ppp1r9a | Gm37309 | Sla | Zfp607b | Mthfr | Ccdc84 |
| Fchsd2 | Inha | Gm37274 | Chkb | Rnf213 | Clcn6 | Als2cl |
| Aff2 | Ssc5d | Gm38220 | Thpo | Mn1 | Gm996 | Dock5 |
| Prr5l | Rsrp1 | Gm38020 | Lmbr1l | Kcnip3 | Col1a2 | Krt14 |
| Arhgap39 | Pkhd1l1 | Gm38077 | Amhr2 | Tnfsf13os | Adamts9 | Onecut2 |
| Evpl | Shank1 | Gm43858 | Satb1 | Gm38394 | Pianp | Slc25a23 |
| Acot11 | Prelp | Gm43511 | Map4k2 | Ptprv | Rhpn2 | Vat1l |
| Rapgefl1 | Fam129c | Gm43413 | Pcyt2 | Xndc1 | Abcd1 | Khnyn |
| Mllt6 | Exoc3l | Gm43445 | Cfh | Pcdhga5 | Col4a2 | Kmt2d |
| Nos1ap | Casc1 | Gm43071 | Ttc14 | Gmpr | Plpp5 | Hoxb3 |
| Col16a1 | Sh2d5 | Gm42603 | Epb41l4b | Col1a1 | Slc27a1 | Asap2 |
| Plekha6 | Smtnl2 | Gm36535 | Ak4 | Grik5 | Fam46a | Plxnb1 |
| Igsf3 | Rimkla | Gm43080 | Mmp17 | Crtc1 | Man2c1 | Usp13 |
| Nrep | Krt9 | Gm43513 | Gigyf1 | Gys1 | Adamts7 | Kmt5c |
| Rnf150 | Mpp3 | Gm43328 | Dmpk | Stx1a | Zic1 | Rpl3 |
| Ldlrad3 | Lgals4 | Gm43693 | Myo7a | Zmiz1 | Nphp3 | Nr6a1 |
| Mical3 | Cpa1 | Gm42820 | Kifc3 | Vav2 | Mst1r | Tacc1 |
| Phactr1 | 9130017K1 | Gm43029 | Jak3 | Pou6f1 | Abcc10 | Sptbn2 |
| Klf12 | Rnf207 | Gm43481 | Tnxb | Adgrl1 | Lss | Clk2 |
| D030028A0 | Clasrp | Gm43747 | Sfxn5 | Lama5 | Szt2 | Rbm47 |
| P2rx3 | Vmn1r13 | Gm42572 | Ppargc1b | Aldoc | Stard9 | Apcdd1 |
| Il16 | Ankrd23 | Gm42732 | Ypel4 | Kdm6b | Zfp446 | Wdr90 |
| Syn3 | Selenbp1 | Gm44243 | Tspoap1 | Rmnd1 | Ogt | Ptar1 |
| Gm37376 | Acrbp | Gm45838 | Adrb1 | Tns3 | Slc25a37 | 6330403L08Rik |
| Papln | 4930518I15 | Gm45221 | Nat14 | Ern1 | Sirt6 | Bnip3 |
| Aldh1l2 | Pdzd7 | Gm9856 | Nfkbiz | Slc9a3r1 | Tet3 | Rab10os |
| Mylk | Gm2420 | Gm47963 | Crb2 | Usp43 | Pcyt1b | Gm2415 |
| Fam151a | Gm9164 | Gm47113 | Zfp57 | Zfp36l1 | Ror1 | Gm7694 |
| Fmnl1 | Gm5466 | Gm47483 | Ppfibp2 | Srsf5 | Ntng2 | |
| Per2 | Gm15344 | Gm33869 | Arhgap33 | Abcd4 | Aldh1b1 | |
| Gm38391 | Gm15337 | Gm47583 | Mycn | Ptch1 | Prpf39 | |
| Srpk3 | Firre | Gm47585 | Bcl9 | Pde4d | Sugp2 | |
| Rpl10 | Gm15513 | Gm47798 | Angptl6 | Hmbox1 | Stag3 | |
| Cdh23 | Chn1os3 | Gm49223 | Whrn | Wnt5a | Uvssa | |
| Kpna2 | BC065397 | AC133488.1 | Lrp1 | Sdf2l1 | Ahdc1 | |
| Cyp46a1 | Gm11266 | Col6a1 | Clcf1 | Map3k8 | Kmt2c | |
| Naprt | C030037D0 | Cldn15 | Crocc | Tcf7l2 | Setd1b | |
| Pgc | Gm23547 | Hmgn2 | Mill2 | Lrrc45 | Higd1a | |

**Table S1a-2**: List of genes increased by Bu versus veh. in siCtrl-transfected cells.

Table S1a-2: List of genes increased by Bu versus veh. in siCtrl-transfected cells.

| | | | | | | |
|---|---|---|---|---|---|---|
| Npy4r | Rpl13a | Pmaip1 | S100a3 | Depdc7 | Thyn1 | 2700099C18Rik |
| Ces2e | Gm8199 | Cdca5 | Tubb6 | Rad51 | Melk | 2610044O15Rik8 |
| Ly6c1 | Fam26e | Kif20b | Foxm1 | Knl1 | Kif15 | AC129328.1 |
| Dusp4 | Perp | Fosl1 | Tubb5 | Cdc25b | Anln | Slc2a9 |
| Ly6a | Rspo3 | Sgo2a | Dbf4 | Knstrn | Grhl3 | Slfn3 |
| Ccl9 | Cox7a2l | Serpinb8 | Cse1l | Mcm8 | Tacc3 | Ms4a10 |
| Serpinb9b | Mmp24 | Nek2 | Kif20a | Tpx2 | Dtl | Pnliprp2 |
| Aldh3a1 | Rps3a1 | Mcm10 | Nqo1 | Ect2 | Rtkn2 | Casq2 |
| Ankrd1 | Glipr2 | Mastl | Asf1b | Gpsm2 | 1700066M | Nppb |
| Dcxr | Slc19a2 | 1700007K1 | Cdc20 | Sass6 | Sccpdh | Cgref1 |
| Cd14 | Rpl37 | Kif18a | Gmnn | Iqgap3 | Fanci | Mylpf |
| Emp1 | S100a13 | Nusap1 | Syngr3 | Smc2 | Neil3 | Ltf |
| 9530053AC | Rps8 | Bub1 | Oit3 | Zfp37 | Exo1 | Mslnl |
| Gna14 | Gm8730 | Aurka | Abca1 | Ndc1 | Bub1b | Gm11223 |
| Aqp3 | Rpl23 | Fam83d | Gadd45b | Rad54l | Pclaf | Gpr85 |
| S100a7a | Fndc10 | Ccna2 | Brca1 | Stil | Siah1b | Slc29a4 |
| Gsto1 | Mt2 | Depdc1a | Mybl2 | Cdca8 | Kif14 | Defb42 |
| Lamb3 | Slc37a2 | Kif2c | Chtf18 | Cenpa | Clspn | 1700003M07Rik |
| Fgf13 | Svop | Abcb1b | Fbxo5 | Ereg | Tuba1c | Gm17655 |
| Plet1 | Mab21l3 | Aass | Sgk1 | Chek2 | Cdc25c | 9430037O13Rik |
| Acta2 | Sec14l5 | Tuba8 | Mdm2 | Mad2l1 | Cenph | Gria3 |
| Anpep | Eda2r | Ddias | Nfyb | Rad18 | Ticrr | Def6 |
| Cd24a | Gen1 | Cenpu | Txnrd1 | Rad51ap1 | Mis18bp1 | Tbc1d8 |
| Nipal1 | Spag5 | Robo3 | Pttg1 | Blm | Fancb | Hspb1 |
| 1110038B1 | Kifc5b | Mcam | Tubd1 | Synm | Sephs2 | Tek |
| Gdf15 | Polq | BC030867 | Ppm1d | Aen | Syt12 | Cct4 |
| Adgrf4 | Arhgap11a | Gna15 | Rrm2 | Arl6ip1 | Haspin | Snrpb2 |
| Rps20 | Cdca2 | Lrr1 | Top2a | Kif22 | Hyls1 | Hyal1 |
| Rps27l | Wnt4 | Ska1 | Vcan | Plk1 | Ercc6l | Rdm1 |
| Eva1c | Tigit | Tubb4b | Cenpk | Mki67 | Tnfaip6 | Mrpl32 |
| Ecscr | Cdc6 | Dlgap5 | Ska3 | Cenpi | Hmgb2 | Hebp2 |
| Rpl39 | Pimreg | Cdkn3 | Esd | Mak16 | Fam72a | Nudcd2 |
| Rps10 | Ccnb2 | Ckap2 | Pbk | Gins3 | Ier5 | Jpt1 |
| Col14a1 | Ube2c | Zfp365 | Gtse1 | Cenpn | Espl1 | Dnaaf2 |
| Duoxa1 | Spc25 | Ttk | Mis18a | Mmp15 | Tubb2a | Efcab11 |
| Cyp2b10 | Nectin4 | Prc1 | Racgap1 | Mns1 | Zfp958 | Dcaf4 |
| Prr7 | Kif11 | Eme1 | Cdkn1a | Kif23 | Nsl1 | Trip13 |
| Mettl7a3 | Ncapg | Atf7ip2 | Poc1a | Gclc | Tubb4a | Fst |
| Alox5ap | Birc5 | Ccnb1 | Cdca3 | Zwilch | Bub3 | Dnajc9 |
| Tubb3 | Cdk1 | Tubb2b | Dusp1 | Traip | Spdl1 | Diaph3 |
| 4930471E1 | Mtfr2 | Cenpe | Incenp | Srxn1 | Fv1 | Eif3e |
| Dram1 | Ccng1 | Frat2 | Fam111a | C330027CC | Ccnf | Ly6e |
| Dglucy | Hmmr | Ckap2l | Cep55 | Ttf2 | Ang | Rfc4 |
| Nme4 | Prr11 | Kif18b | Plk4 | Aspm | Tuba1a | Pros1 |
| Cyp2c55 | Aurkb | Spn | Bard1 | Fancd2 | Spc24 | Cbr3 |
| Spint1 | Cenpp | Nrg1 | Tuba4a | Kif4 | Slc4a11 | Thbs2 |
| Rps3 | Depdc1b | Oip5 | Ube2t | Trim59 | Gas2l3 | Pigf |
| Phlda3 | Esco2 | Rprm | Cenpf | Brip1 | Cenpw | Rps14 |
| Ctla2a | Shcbp1 | Aunip | Suv39h2 | Smc4 | Kifc1 | Fas |
| Cd109 | Cenpq | 2010110K1 | Nuf2 | Ncaph | Haus3 | Crcp |
| Gm6030 | Sgo1 | Gm36401 | Cenpl | Nup37 | 2810408I1 | Stk17b |
| Ethe1 | Ndc80 | Cdc45 | Sapcd2 | Parpbp | 9230114K1 | Irf6 |

Table S1a-2: List of genes increased by Bu versus veh. in siCtrl-transfected cells.

| | |
|---|---|
| Phospho2 | |
| Eif3m | Slfn2 |
| Fabp5 | 5430403G16Rik |
| Exosc9 | Osgin1 |
| Tlr2 | Smim26 |
| Polr1e | Arrdc3 |
| Prdx1 | Rad54b |
| Stmn1 | Zfp429 |
| Rcc1 | Gm14681 |
| Lzic | Gm20667 |
| Triap1 | Zfp960 |
| Mphosph1( | Zfp850 |
| Ap1s2 | |
| Vps36 | |
| Mfap3l | |
| Tbc1d9 | |
| Ccsap | |
| Abhd10 | |
| Reep4 | |
| Tmem5 | |
| Celf5 | |
| Trim13 | |
| Calm2 | |
| Cib2 | |
| Rpf2 | |
| Ephx1 | |
| Myl6b | |
| Rbm48 | |
| Pitpnc1 | |
| St8sia4 | |
| Gfer | |
| Rpl7 | |
| Tnfaip8l1 | |
| Cxcr4 | |
| Dlk2 | |
| Mlf1 | |
| Fam212b | |
| Fam241a | |
| Ubald2 | |
| Insl6 | |
| Nat2 | |
| Zfp472 | |
| Glipr1 | |
| Zfp729b | |
| Ube2s | |
| Ei24 | |
| Arhgap22 | |
| Vrk2 | |
| Syt11 | |
| Zfp874a | |
| Tmem19 | |
| Nanos1 | |

**Table S1b**: List of genes affected by Bu versus veh. in siTgr5-transfected cells.

Table S1b: List of genes affected by Bu versus veh. in siTgr5-transfected cells.

| | | | | | | |
|---|---|---|---|---|---|---|
| Scd1 | Slc2a4rg-ps | Adamts2 | Klhl29 | Itgb8 | Trib2 | Fggy |
| Il1rn | Gm12940 | Fgf1 | Slc17a9 | Nkain4 | Epas1 | Tsc22d3 |
| Naaladl2 | Gdap10 | Arl5c | Gabbr1 | Mmp16 | Col5a1 | Thsd4 |
| Adgrl3 | 2610037D02 | Atp5l | Apba1 | Nup210 | Kcnab1 | Magi2 |
| Sox5 | Gm42664 | Zdhhc1 | Plce1 | Lctl | Itpr1 | Gda |
| Prkg1 | Gm43128 | Cdh18 | Nrp2 | Nktr | Psd3 | Eda |
| Immp2l | Gm35339 | Arglu1 | 37500 | Igdcc4 | Fchsd2 | Cxcl12 |
| Angptl7 | 9930017N22 | Tet2 | Dclk2 | Ank2 | Aff2 | A330074K22Rik |
| Ung | AC154232.2 | Adamts3 | Per3 | Wnk4 | Prr5l | Nav1 |
| Ptprm | Asic1 | 2900005J15 | Pcsk6 | Ints6l | Arhgap39 | Deptor |
| Gpc6 | Agap2 | Adgrd1 | Tenm3 | Arhgap26 | Evpl | Sil1 |
| Gas7 | Zfr2 | Rnf182 | Ulk3 | Spred3 | Acot11 | Clybl |
| Kifc2 | Zcchc7 | Tenm4 | Loxl1 | Tgm2 | Rapgefl1 | Dnm1 |
| Col20a1 | Pitpnm3 | Lingo1 | Pknox2 | Ccr7 | Mllt6 | Thsd7a |
| Cacna1g | Gapdh | Pard3b | Csgalnact1 | Camk4 | Nos1ap | Grip1 |
| Vdr | Trabd2b | Slit3 | Gtdc1 | Ak5 | Col16a1 | Zfp503 |
| Cd200 | Snhg17 | Hmcn1 | Stox2 | Ncam1 | Plekha6 | Ydjc |
| Fa2h | Malat1 | Dnm3os | Kazn | Plekhg5 | Igsf3 | Asxl3 |
| Flrt1 | Pdzd2 | Airn | Atxn1 | Six5 | Nrep | Tmem202 |
| Kcnq3 | Podxl | Prkcg | Sox6 | Kif21b | Rnf150 | Dock1 |
| Megf6 | Smpd3 | B930095G15 | D930048N14 | Nemp2 | Ldlrad3 | Adamts14 |
| Zfp773 | Sema5a | Wipf3 | Gas1 | Eml6 | Mical3 | Efnb3 |
| 1700001L05 | Adamtsl1 | Gm15675 | Slc4a4 | Ttc41 | Phactr1 | Tmem204 |
| Sbk3 | 3110039I08 | 4933407K13 | Maml3 | Magi1 | Klf12 | Gbp8 |
| AI480526 | Gm21781 | 2900089D17 | Zfp467 | Gpr68 | D030028A08 | Bmp7 |
| Gm37893 | Gm20342 | Neat1 | Olfr1372-ps1 | Gli2 | Npy4r | Ppm1l |
| Adgrb2 | Gm20045 | Gm29666 | Ube2i | Nckap5 | Ltbp2 | Cxcl10 |
| Spag1 | Gm44200 | Gm28941 | Cacnb1 | Zfp579 | Eya4 | Tmem255a |
| Fzd4 | Xylt1 | Gm38057 | Pabpn1 | Xkr4 | Gamt | Kctd14 |
| Il33 | Col4a4 | Gm36936 | Npff | Nav2 | Kif26b | Gm14443 |
| Rspo1 | Alcam | Gm36989 | Adamts10 | Tln2 | Lpp | Gm26532 |
| Fras1 | Sorcs1 | 6430511E19 | Gpc2 | Grk4 | Exoc6b | 6720427I07Rik |
| Csdc2 | Dhrs3 | A930004J17 | Gabre | Nebl | Sod3 | Gm44423 |
| Kcnb1 | Cfp | Gm38253 | Igsf9 | Slc8a1 | Large1 | Pisd-ps2 |
| Fbln5 | Hoxc8 | Gm38372 | Fam227a | Ppp4r1l-ps | Ptprg | Igfbp7 |
| Adamts20 | Apoe | Gm37206 | Kcp | Syngap1 | Matn2 | Col8a1 |
| Extl1 | Aqp1 | Gm38248 | Actg1 | Msi2 | Slc39a10 | 9230112E08Rik |
| Tmem108 | 1700109H08 | Gm42979 | Grid2 | Prob1 | Dclk1 | 2900076A07Rik |
| Col8a2 | Matn4 | Gm43759 | Tgfbr3l | Mob3b | Cdk14 | Spib |
| Mir22hg | Hoxc5 | Gm43300 | Pla2g4b | Carns1 | Gmds | Npas3 |
| Zfp950 | Pcdhgc4 | Gm43059 | Gm37261 | Nrbp2 | Plcb4 | Slc7a4 |
| Slc1a2 | Sfi1 | Gm42549 | Gm37963 | Smim1 | Ulk4 | St6gal1 |
| Tnfrsf25 | Slc25a27 | Gm44597 | Gm42913 | C430049B03 | Babam2 | Mapk4 |
| Slc27a3 | Atp6v0c | Gm44667 | Gm42876 | D430040D24 | St6galnac3 | Rabgap1l |
| Prss12 | Tnik | Gm45632 | Gm43327 | Gm17491 | Ffar4 | Kif5c |
| Ttll3 | Sorcs2 | Gm45250 | Hk2 | 5033428I22 | Wwox | Cpxm1 |
| Leng8 | Plxna4 | AC160637.1 | Slc4a3 | Slc1a5 | Tcp11l2 | Col11a1 |
| Atp8a1 | St8sia1 | Gm47260 | Esr1 | Celf2 | Setbp1 | Kcnq5 |
| Ankrd16 | Plxna3 | Gm49284 | Itgb3 | Ppp1cc | Plxna2 | Medag |
| Il18bp | Pygm | Gm49204 | Cygb | Chrd | Frmd5 | Dync1i1 |
| Spaca6 | Unc13a | AC105358.1 | Nxnl2 | Nfib | Adgrl2 | Slc9a9 |
| Gm11400 | Tmtc2 | Lama4 | Gfra1 | Appl2 | Ptprd | Kirrel3 |

Table S1b: List of genes affected by Bu versus veh. in siTgr5-transfected cells.

| | | | | | | |
|---|---|---|---|---|---|---|
| Tle2 | Zfpm2 | Gpr157 | Svop | Aass | Sgk1 | Chek2 |
| B3galt1 | Mtss1 | Prex2 | Mab21l3 | Tuba8 | Mdm2 | Mad2l1 |
| Gm49322 | Cblb | Tnfsf15 | Sec14l5 | Ddias | Nfyb | Rad18 |
| Syt1 | Gpd1 | Lypd6 | Eda2r | Cenpu | Txnrd1 | Rad51ap1 |
| Hist1h1c | Tbc1d5 | Pbx1 | Gen1 | Robo3 | Pttg1 | Blm |
| Tspan15 | Fbxl17 | Ebf4 | Spag5 | Mcam | Tubd1 | Synm |
| Rims2 | C3 | Agap1 | Kifc5b | BC030867 | Ppm1d | Aen |
| Ccser1 | Myo1a | Smyd3 | Polq | Gna15 | Rrm2 | Arl6ip1 |
| Bach2 | Fbxl16 | Maf | Arhgap11a | Lrr1 | Top2a | Kif22 |
| Cfap69 | Cd55 | Cd248 | Cdca2 | Ska1 | Vcan | Plk1 |
| Erc2 | Glul | Nbeal2 | Tigit | Tubb4b | Cenpk | Mki67 |
| Ust | Plxdc2 | Kcnd2 | Cdc6 | Dlgap5 | Ska3 | Cenpi |
| Trappc9 | Rassf2 | Jazf1 | Pimreg | Cdkn3 | Esd | Mak16 |
| Prkca | Nup210l | Slc24a3 | Ccnb2 | Ckap2 | Pbk | Gins3 |
| Cacna1c | Lrba | Fam172a | Ube2c | Zfp365 | Gtse1 | Cenpn |
| Robo2 | Tgfbr3 | Mir186 | Spc25 | Ttk | Mis18a | Mmp15 |
| Atrnl1 | Ephb6 | Samd1 | Nectin4 | Prc1 | Racgap1 | Mns1 |
| Fut2 | Sergef | C230037L18 | Kif11 | Eme1 | Cdkn1a | Kif23 |
| St3gal5 | Hpx | Gm15265 | Ncapg | Atf7ip2 | Poc1a | Gclc |
| Lepr | Porcn | Mypopos | Birc5 | Ccnb1 | Cdca3 | Zwilch |
| Samd12 | Gabra3 | D430018E03 | Cdk1 | Tubb2b | Dusp1 | Traip |
| Unc5c | Arhgap6 | Gm26935 | Mtfr2 | Cenpe | Incenp | Srxn1 |
| Kalrn | Maml2 | Gm27010 | Ccng1 | Frat2 | Fam111a | C330027C09Rik |
| Snx29 | Arhgap44 | Gm37694 | Hmmr | Ckap2l | Cep55 | Ttf2 |
| Mroh2a | Vav3 | Gm37969 | Prr11 | Kif18b | Plk4 | Aspm |
| Col4a3 | Fhod3 | Gm42868 | Aurkb | Spn | Bard1 | Fancd2 |
| 5430405H02 | Diaph2 | BC030343 | Cenpp | Nrg1 | Tuba4a | Kif4 |
| Lrrc32 | Ptpn13 | Gm42748 | Depdc1b | Oip5 | Ube2t | Trim59 |
| Gm26621 | Mitf | Gm43654 | Esco2 | Rprm | Cenpf | Brip1 |
| Tmem147os | Dagla | Gm43268 | Shcbp1 | Aunip | Suv39h2 | Smc4 |
| Gm38190 | Bbs9 | Gm44220 | Cenpq | 2010110K18 | Nuf2 | Ncaph |
| Gm37249 | Dock4 | Gm44321 | Sgo1 | Gm36401 | Cenpl | Nup37 |
| Gm42635 | Mgat5 | Gm44168 | Ndc80 | Cdc45 | Sapcd2 | Parpbp |
| Gm45234 | Prickle1 | 2610028D06 | Pmaip1 | S100a3 | Depdc7 | Thyn1 |
| Gm43890 | Kctd1 | Gm47205 | Cdca5 | Tubb6 | Rad51 | Melk |
| Gm47976 | Zic4 | Gm48804 | Kif20b | Foxm1 | Knl1 | Kif15 |
| AC131339.2 | Fgf2 | mt-Nd6 | Fosl1 | Tubb5 | Cdc25b | Anln |
| Rasa4 | Sh3rf3 | P2rx3 | Sgo2a | Dbf4 | Knstrn | Grhl3 |
| Atp5g1 | Cntln | Ces2e | Serpinb8 | Cse1l | Mcm8 | Tacc3 |
| Cdkal1 | Tbc1d32 | Ly6c1 | Nek2 | Kif20a | Tpx2 | Dtl |
| Ubxn11 | Creb3l2 | Dusp4 | Mcm10 | Nqo1 | Ect2 | Rtkn2 |
| Mertk | Sema6c | Ly6a | Mastl | Asf1b | Gpsm2 | 1700066M21Rik |
| Arnt2 | Kif16b | Ccl9 | 1700007K13 | Cdc20 | Sass6 | Sccpdh |
| B4galt5 | Cacna2d1 | Serpinb9b | Kif18a | Gmnn | Iqgap3 | Fanci |
| Isyna1 | Rfx3 | Aldh3a1 | Nusap1 | Syngr3 | Smc2 | Neil3 |
| Rhobtb1 | Elmo1 | Ankrd1 | Bub1 | Oit3 | Zfp37 | Exo1 |
| Slc46a1 | Dcp1b | Dcxr | Aurka | Abca1 | Ndc1 | Bub1b |
| Fbxl20 | Slc39a11 | Cd14 | Fam83d | Gadd45b | Rad54l | Pclaf |
| Carmil1 | Arl15 | Emp1 | Ccna2 | Brca1 | Stil | Siah1b |
| Fars2 | Prkce | 9530053A07 | Depdc1a | Mybl2 | Cdca8 | Kif14 |
| Atp8a2 | Ctdspl | Mt2 | Kif2c | Chtf18 | Cenpa | Clspn |
| Tdrd3 | Gphn | Slc37a2 | Abcb1b | Fbxo5 | Ereg | Tuba1c |

Table S1b: List of genes affected by Bu versus veh. in siTgr5-transfected cells.

| | | | |
|---|---|---|---|
| Cdc25c | Fgd3 | Cyr61 | Gm44956 |
| Cenph | Acer2 | Gem | A030001D20Rik |
| Ticrr | Mreg | Npr2 | Gm47813 |
| Mis18bp1 | Inhba | Rpa2 | AC130815.2 |
| Fancb | Fut10 | Sesn2 | |
| Sephs2 | Aldh1a1 | Kntc1 | |
| Syt12 | Rnd1 | Cit | |
| Haspin | Ces2f | Siglecg | |
| Hyls1 | 2810403D21 | Eri2 | |
| Ercc6l | Mirt1 | Rbmx2 | |
| Tnfaip6 | 5033406O09 | Fgl1 | |
| Hmgb2 | Gsta3 | Snx20 | |
| Fam72a | Prrg4 | Mb21d1 | |
| Ier5 | Slc7a11 | Recql4 | |
| Espl1 | Gpnmb | Lsm3 | |
| Tubb2a | Mctp2 | G2e3 | |
| Zfp958 | Apol6 | Haus8 | |
| Nsl1 | Tmem71 | Fignl1 | |
| Tubb4a | Gdf5 | Wdr62 | |
| Bub3 | Cd302 | Ncapd2 | |
| Spdl1 | Gm30246 | Atp6v0a4 | |
| Fv1 | Serpina3n | Suv39h1 | |
| Ccnf | Aldh1a7 | Syngr4 | |
| Ang | P2rx7 | Arhgap9 | |
| Tuba1a | Pglyrp3 | Ckap5 | |
| Spc24 | Fgfbp1 | Cep126 | |
| Slc4a11 | Afp | Tnfrsf18 | |
| Gas2l3 | Gm11478 | Ncapg2 | |
| Cenpw | Ifrd1 | Mum1l1 | |
| Kifc1 | Dennd2c | D030056L22Rik | |
| Haus3 | Relt | Cdca4 | |
| 2810408I11 | Steap1 | Krt8 | |
| 9230114K14 | Calr3 | Hmx2 | |
| 2700099C18 | Dcbld1 | Arhgef39 | |
| Veph1 | Ska2 | Nxf3 | |
| Ptx3 | Foxg1 | Lin9 | |
| Ptgs2 | Edn1 | Slco1a5 | |
| Styk1 | Polk | Cenpj | |
| Rbp2 | Plk2 | mt-Tw | |
| Serpinb6c | Grhl2 | Gstm6 | |
| Kcnn4 | Rangap1 | Slfn10-ps | |
| Mt1 | Tuba1b | Gm13230 | |
| Slc30a1 | Cbs | Gm16278 | |
| Flrt3 | Fen1 | Trp53cor1 | |
| Lyz2 | Hells | BC039966 | |
| Has2 | Mybl1 | Snhg5 | |
| Wnt7b | Terf1 | 4930558J18Rik | |
| Col2a1 | Nup35 | 4930461G14Rik | |
| Noct | Gins1 | 2210011K15Rik | |
| Des | Rbm38 | Dchs2 | |
| Rragd | Ddx20 | Gm3716 | |
| Troap | Fam198b | Gm38973 | |

**Table S1b-1**: List of genes decreased by Bu versus veh. in siTgr5-transfected cells.

Table S1b-1: List of genes decreased by Bu versus veh. in siTgr5-transfected cells.

| | | | | | | |
|---|---|---|---|---|---|---|
| Scd1 | Slc2a4rg-ps | Adamts2 | Klhl29 | Itgb8 | Trib2 | Fggy |
| Il1rn | Gm12940 | Fgf1 | Slc17a9 | Nkain4 | Epas1 | Tsc22d3 |
| Naaladl2 | Gdap10 | Arl5c | Gabbr1 | Mmp16 | Col5a1 | Thsd4 |
| Adgrl3 | 2610037D0 | Atp5l | Apba1 | Nup210 | Kcnab1 | Magi2 |
| Sox5 | Gm42664 | Zdhhc1 | Plce1 | Lctl | Itpr1 | Gda |
| Prkg1 | Gm43128 | Cdh18 | Nrp2 | Nktr | Psd3 | Eda |
| Immp2l | Gm35339 | Arglu1 | sept-02 | Igdcc4 | Fchsd2 | Cxcl12 |
| Angptl7 | 9930017N2 | Tet2 | Dclk2 | Ank2 | Aff2 | A330074K22Rik |
| Ung | AC154232.2 | Adamts3 | Per3 | Wnk4 | Prr5l | Nav1 |
| Ptprm | Asic1 | 2900005J15 | Pcsk6 | Ints6l | Arhgap39 | Deptor |
| Gpc6 | Agap2 | Adgrd1 | Tenm3 | Arhgap26 | Evpl | Sil1 |
| Gas7 | Zfr2 | Rnf182 | Ulk3 | Spred3 | Acot11 | Clybl |
| Kifc2 | Zcchc7 | Tenm4 | Loxl1 | Tgm2 | Rapgefl1 | Dnm1 |
| Col20a1 | Pitpnm3 | Lingo1 | Pknox2 | Ccr7 | Mllt6 | Thsd7a |
| Cacna1g | Gapdh | Pard3b | Csgalnact1 | Camk4 | Nos1ap | Grip1 |
| Vdr | Trabd2b | Slit3 | Gtdc1 | Ak5 | Col16a1 | Zfp503 |
| Cd200 | Snhg17 | Hmcn1 | Stox2 | Ncam1 | Plekha6 | Ydjc |
| Fa2h | Malat1 | Dnm3os | Kazn | Plekhg5 | Igsf3 | Asxl3 |
| Flrt1 | Pdzd2 | Airn | Atxn1 | Six5 | Nrep | Tmem202 |
| Kcnq3 | Podxl | Prkcg | Sox6 | Kif21b | Rnf150 | Dock1 |
| Megf6 | Smpd3 | B930095G1 | D930048N1 | Nemp2 | Ldlrad3 | Adamts14 |
| Zfp773 | Sema5a | Wipf3 | Gas1 | Eml6 | Mical3 | Efnb3 |
| 1700001L0 | Adamtsl1 | Gm15675 | Slc4a4 | Ttc41 | Phactr1 | Tmem204 |
| Sbk3 | 3110039I08 | 4933407K1 | Maml3 | Magi1 | Klf12 | Gbp8 |
| AI480526 | Gm21781 | 2900089D1 | Zfp467 | Gpr68 | D030028A0 | Bmp7 |
| Gm37893 | Gm20342 | Neat1 | Olfr1372-ps | Gli2 | Npy4r | Ppm1l |
| Adgrb2 | Gm20045 | Gm29666 | Ube2i | Nckap5 | Ltbp2 | Cxcl10 |
| Spag1 | Gm44200 | Gm28941 | Cacnb1 | Zfp579 | Eya4 | Tmem255a |
| Fzd4 | Xylt1 | Gm38057 | Pabpn1 | Xkr4 | Gamt | Kctd14 |
| Il33 | Col4a4 | Gm36936 | Npff | Nav2 | Kif26b | Gm14443 |
| Rspo1 | Alcam | Gm36989 | Adamts10 | Tln2 | Lpp | Gm26532 |
| Fras1 | Sorcs1 | 6430511E19 | Gpc2 | Grk4 | Exoc6b | 6720427I07Rik |
| Csdc2 | Dhrs3 | A930004J17 | Gabre | Nebl | Sod3 | Gm44423 |
| Kcnb1 | Cfp | Gm38253 | Igsf9 | Slc8a1 | Large1 | Pisd-ps2 |
| Fbln5 | Hoxc8 | Gm38372 | Fam227a | Ppp4r1l-ps | Ptprg | Igfbp7 |
| Adamts20 | Apoe | Gm37206 | Kcp | Syngap1 | Matn2 | Col8a1 |
| Extl1 | Aqp1 | Gm38248 | Actg1 | Msi2 | Slc39a10 | 9230112E08Rik |
| Tmem108 | 1700109H0 | Gm42979 | Grid2 | Prob1 | Dclk1 | 2900076A07Rik |
| Col8a2 | Matn4 | Gm43759 | Tgfbr3l | Mob3b | Cdk14 | Spib |
| Mir22hg | Hoxc5 | Gm43300 | Pla2g4b | Carns1 | Gmds | Npas3 |
| Zfp950 | Pcdhgc4 | Gm43059 | Gm37261 | Nrbp2 | Plcb4 | Slc7a4 |
| Slc1a2 | Sfi1 | Gm42549 | Gm37963 | Smim1 | Ulk4 | St6gal1 |
| Tnfrsf25 | Slc25a27 | Gm44597 | Gm42913 | C430049B0 | Babam2 | Mapk4 |
| Slc27a3 | Atp6v0c | Gm44667 | Gm42876 | D430040D2 | St6galnac3 | Rabgap1l |
| Prss12 | Tnik | Gm45632 | Gm43327 | Gm17491 | Ffar4 | Kif5c |
| Ttll3 | Sorcs2 | Gm45250 | Hk2 | 5033428I22 | Wwox | Cpxm1 |
| Leng8 | Plxna4 | AC160637.1 | Slc4a3 | Slc1a5 | Tcp11l2 | Col11a1 |
| Atp8a1 | St8sia1 | Gm47260 | Esr1 | Celf2 | Setbp1 | Kcnq5 |
| Ankrd16 | Plxna3 | Gm49284 | Itgb3 | Ppp1cc | Plxna2 | Medag |
| Il18bp | Pygm | Gm49204 | Cygb | Chrd | Frmd5 | Dync1i1 |
| Spaca6 | Unc13a | AC105358.1 | Nxnl2 | Nfib | Adgrl2 | Slc9a9 |
| Gm11400 | Tmtc2 | Lama4 | Gfra1 | Appl2 | Ptprd | Kirrel3 |

Table S1b-1: List of genes decreased by Bu versus veh. in siTgr5-transfected cells.

| | | |
|---|---|---|
| Tle2 | Zfpm2 | Gpr157 |
| B3galt1 | Mtss1 | Prex2 |
| Gm49322 | Cblb | Tnfsf15 |
| Syt1 | Gpd1 | Lypd6 |
| Hist1h1c | Tbc1d5 | Pbx1 |
| Tspan15 | Fbxl17 | Ebf4 |
| Rims2 | C3 | Agap1 |
| Ccser1 | Myo1a | Smyd3 |
| Bach2 | Fbxl16 | Maf |
| Cfap69 | Cd55 | Cd248 |
| Erc2 | Glul | Nbeal2 |
| Ust | Plxdc2 | Kcnd2 |
| Trappc9 | Rassf2 | Jazf1 |
| Prkca | Nup210l | Slc24a3 |
| Cacna1c | Lrba | Fam172a |
| Robo2 | Tgfbr3 | Mir186 |
| Atrnl1 | Ephb6 | Samd1 |
| Fut2 | Sergef | C230037L18Rik |
| St3gal5 | Hpx | Gm15265 |
| Lepr | Porcn | Mypopos |
| Samd12 | Gabra3 | D430018E03Rik |
| Unc5c | Arhgap6 | Gm26935 |
| Kalrn | Maml2 | Gm27010 |
| Snx29 | Arhgap44 | Gm37694 |
| Mroh2a | Vav3 | Gm37969 |
| Col4a3 | Fhod3 | Gm42868 |
| 5430405H0 | Diaph2 | BC030343 |
| Lrrc32 | Ptpn13 | Gm42748 |
| Gm26621 | Mitf | Gm43654 |
| Tmem147os | Dagla | Gm43268 |
| Gm38190 | Bbs9 | Gm44220 |
| Gm37249 | Dock4 | Gm44321 |
| Gm42635 | Mgat5 | Gm44168 |
| Gm45234 | Prickle1 | 2610028D06Rik |
| Gm43890 | Kctd1 | Gm47205 |
| Gm47976 | Zic4 | Gm48804 |
| AC131339.2 | Fgf2 | |
| Rasa4 | Sh3rf3 | |
| Atp5g1 | Cntln | |
| Cdkal1 | Tbc1d32 | |
| Ubxn11 | Creb3l2 | |
| Mertk | Sema6c | |
| Arnt2 | Kif16b | |
| B4galt5 | Cacna2d1 | |
| Isyna1 | Rfx3 | |
| Rhobtb1 | Elmo1 | |
| Slc46a1 | Dcp1b | |
| Fbxl20 | Slc39a11 | |
| Carmil1 | Arl15 | |
| Fars2 | Prkce | |
| Atp8a2 | Ctdspl | |
| Tdrd3 | Gphn | |

**Table S1b-2**: List of genes increased by Bu versus veh. in siTgr5-transfected cells.

Table S1b-2: List of genes increased by Bu versus veh. in siTgr5-transfected cells.

| | | | | | | | |
|---|---|---|---|---|---|---|---|
| mt-Nd6 | Fosl1 | Tubb5 | Cdc25b | Anln | Ptgs2 | Edn1 | Slco1a5 |
| P2rx3 | Sgo2a | Dbf4 | Knstrn | Grhl3 | Styk1 | Polk | Cenpj |
| Ces2e | Serpinb8 | Cse1l | Mcm8 | Tacc3 | Rbp2 | Plk2 | mt-Tw |
| Ly6c1 | Nek2 | Kif20a | Tpx2 | Dtl | Serpinb6c | Grhl2 | Gstm6 |
| Dusp4 | Mcm10 | Nqo1 | Ect2 | Rtkn2 | Kcnn4 | Rangap1 | Slfn10-ps |
| Ly6a | Mastl | Asf1b | Gpsm2 | 1700066M2 | Mt1 | Tuba1b | Gm13230 |
| Ccl9 | 1700007K | Cdc20 | Sass6 | Sccpdh | Slc30a1 | Cbs | Gm16278 |
| Serpinb9b | Kif18a | Gmnn | Iqgap3 | Fanci | Flrt3 | Fen1 | Trp53cor1 |
| Aldh3a1 | Nusap1 | Syngr3 | Smc2 | Neil3 | Lyz2 | Hells | BC039966 |
| Ankrd1 | Bub1 | Oit3 | Zfp37 | Exo1 | Has2 | Mybl1 | Snhg5 |
| Dcxr | Aurka | Abca1 | Ndc1 | Bub1b | Wnt7b | Terf1 | 4930558J18Rik |
| Cd14 | Fam83d | Gadd45b | Rad54l | Pclaf | Col2a1 | Nup35 | 4930461G14Rik |
| Emp1 | Ccna2 | Brca1 | Stil | Siah1b | Noct | Gins1 | 2210011K15Rik |
| 9530053A07F | Depdc1a | Mybl2 | Cdca8 | Kif14 | Des | Rbm38 | Dchs2 |
| Mt2 | Kif2c | Chtf18 | Cenpa | Clspn | Rragd | Ddx20 | Gm3716 |
| Slc37a2 | Abcb1b | Fbxo5 | Ereg | Tuba1c | Troap | Fam198l | Gm38973 |
| Svop | Aass | Sgk1 | Chek2 | Cdc25c | Fgd3 | Cyr61 | Gm44956 |
| Mab21l3 | Tuba8 | Mdm2 | Mad2l1 | Cenph | Acer2 | Gem | A030001D20Rik |
| Sec14l5 | Ddias | Nfyb | Rad18 | Ticrr | Mreg | Npr2 | Gm47813 |
| Eda2r | Cenpu | Txnrd1 | Rad51ap1 | Mis18bp1 | Inhba | Rpa2 | AC130815.2 |
| Gen1 | Robo3 | Pttg1 | Blm | Fancb | Fut10 | Sesn2 | |
| Spag5 | Mcam | Tubd1 | Synm | Sephs2 | Aldh1a1 | Kntc1 | |
| Kifc5b | BC030867 | Ppm1d | Aen | Syt12 | Rnd1 | Cit | |
| Polq | Gna15 | Rrm2 | Arl6ip1 | Haspin | Ces2f | Siglecg | |
| Arhgap11a | Lrr1 | Top2a | Kif22 | Hyls1 | 2810403D | Eri2 | |
| Cdca2 | Ska1 | Vcan | Plk1 | Ercc6l | Mirt1 | Rbmx2 | |
| Tigit | Tubb4b | Cenpk | Mki67 | Tnfaip6 | 5033406O | Fgl1 | |
| Cdc6 | Dlgap5 | Ska3 | Cenpi | Hmgb2 | Gsta3 | Snx20 | |
| Pimreg | Cdkn3 | Esd | Mak16 | Fam72a | Prrg4 | Mb21d1 | |
| Ccnb2 | Ckap2 | Pbk | Gins3 | Ier5 | Slc7a11 | Recql4 | |
| Ube2c | Zfp365 | Gtse1 | Cenpn | Espl1 | Gpnmb | Lsm3 | |
| Spc25 | Ttk | Mis18a | Mmp15 | Tubb2a | Mctp2 | G2e3 | |
| Nectin4 | Prc1 | Racgap1 | Mns1 | Zfp958 | Apol6 | Haus8 | |
| Kif11 | Eme1 | Cdkn1a | Kif23 | Nsl1 | Tmem71 | Fignl1 | |
| Ncapg | Atf7ip2 | Poc1a | Gclc | Tubb4a | Gdf5 | Wdr62 | |
| Birc5 | Ccnb1 | Cdca3 | Zwilch | Bub3 | Cd302 | Ncapd2 | |
| Cdk1 | Tubb2b | Dusp1 | Traip | Spdl1 | Gm30246 | Atp6v0a4 | |
| Mtfr2 | Cenpe | Incenp | Srxn1 | Fv1 | Serpina3n | Suv39h1 | |
| Ccng1 | Frat2 | Fam111a | C330027C09 | Ccnf | Aldh1a7 | Syngr4 | |
| Hmmr | Ckap2l | Cep55 | Ttf2 | Ang | P2rx7 | Arhgap9 | |
| Prr11 | Kif18b | Plk4 | Aspm | Tuba1a | Pglyrp3 | Ckap5 | |
| Aurkb | Spn | Bard1 | Fancd2 | Spc24 | Fgfbp1 | Cep126 | |
| Cenpp | Nrg1 | Tuba4a | Kif4 | Slc4a11 | Afp | Tnfrsf18 | |
| Depdc1b | Oip5 | Ube2t | Trim59 | Gas2l3 | Gm11478 | Ncapg2 | |
| Esco2 | Rprm | Cenpf | Brip1 | Cenpw | Ifrd1 | Mum1l1 | |
| Shcbp1 | Aunip | Suv39h2 | Smc4 | Kifc1 | Dennd2c | D030056L22Rik | |
| Cenpq | 2010110K | Nuf2 | Ncaph | Haus3 | Relt | Cdca4 | |
| Sgo1 | Gm36401 | Cenpl | Nup37 | 2810408I11 | Steap1 | Krt8 | |
| Ndc80 | Cdc45 | Sapcd2 | Parpbp | 9230114K14 | Calr3 | Hmx2 | |
| Pmaip1 | S100a3 | Depdc7 | Thyn1 | 2700099C18 | Dcbld1 | Arhgef39 | |
| Cdca5 | Tubb6 | Rad51 | Melk | Veph1 | Ska2 | Nxf3 | |
| Kif20b | Foxm1 | Knl1 | Kif15 | Ptx3 | Foxg1 | Lin9 | |

**Table S1c**: List of genes affected by Bu specifically in siCtrl-transfected cells.

Table S1c: List of genes affected by Bu specifically in siCtrl-transfected cells.

| | | | | | | |
|---|---|---|---|---|---|---|
| Mgp | AC162302.2 | Atp1b2 | Krt9 | Gm43513 | Gigyf1 | Gys1 |
| Ckmt1 | Rrnad1 | Ppp1r3b | Mpp3 | Gm43328 | Dmpk | Stx1a |
| Ttc22 | Anxa9 | Prdm9 | Lgals4 | Gm43693 | Myo7a | Zmiz1 |
| Med12l | P4ha2 | Haghl | Cpa1 | Gm42820 | Kifc3 | Vav2 |
| Gm28151 | Col6a2 | Msln | 9130017K11I | Gm43029 | Jak3 | Pou6f1 |
| Rnf112 | Nid2 | Rab44 | Rnf207 | Gm43481 | Tnxb | Adgrl1 |
| Slc22a17 | 6030458C11I | Tia1 | Clasrp | Gm43747 | Sfxn5 | Lama5 |
| Usp53 | Ly6f | Emc1 | Vmn1r13 | Gm42572 | Ppargc1b | Aldoc |
| Abca9 | Paxbp1 | Il16 | Ankrd23 | Gm42732 | Ypel4 | Kdm6b |
| Fbxl7 | Krt7 | Syn3 | Selenbp1 | Gm44243 | Tspoap1 | Rmnd1 |
| Snx32 | Wfdc1 | Gm37376 | Acrbp | Gm45838 | Adrb1 | Tns3 |
| 5430430B14I | Vegfa | Papln | 4930518I15R | Gm45221 | Nat14 | Ern1 |
| Susd2 | Ikzf2 | Aldh1l2 | Pdzd7 | Gm9856 | Nfkbiz | Slc9a3r1 |
| Rhbdl3 | Ccnt2 | Mylk | Gm2420 | Gm47963 | Crb2 | Usp43 |
| Lcor | Sema6d | Fam151a | Gm9164 | Gm47113 | Zfp57 | Zfp36l1 |
| Slco5a1 | Ccnl1 | Fmnl1 | Gm5466 | Gm47483 | Ppfibp2 | Srsf5 |
| Rnft2 | Plxnb3 | Per2 | Gm15344 | Gm33869 | Arhgap33 | Abcd4 |
| Plekha7 | Car12 | Gm38391 | Gm15337 | Gm47583 | Mycn | Ptch1 |
| Fgf18 | Zfc3h1 | Srpk3 | Firre | Gm47585 | Bcl9 | Pde4d |
| Pisd-ps1 | Sv2a | Rpl10 | Gm15513 | Gm47798 | Angptl6 | Hmbox1 |
| Dennd6b | Gjb3 | Cdh23 | Chn1os3 | Gm49223 | Whrn | Wnt5a |
| Ager | Synpo | Kpna2 | BC065397 | AC133488.1 | Lrp1 | Sdf2l1 |
| Rac3 | Il17re | Cyp46a1 | Gm11266 | Col6a1 | Clcf1 | Map3k8 |
| Adcy1 | Zbtb37 | Naprt | C030037D09I | Cldn15 | Crocc | Tcf7l2 |
| Dnase1l2 | Sv2c | Pgc | Gm23547 | Hmgn2 | Mill2 | Lrrc45 |
| Mapk8ip3 | Dock8 | Pi16 | Gm16574 | Eno2 | Dennd4b | Slc16a3 |
| Nudt8 | Ggcx | Rgs11 | Gm17024 | Pdk1 | Hoxd11 | Mbd6 |
| Lrp2 | Dleu2 | Mamdc4 | Mir5125 | Runx1t1 | Cilp2 | Pdia4 |
| Pnisr | Zc3h11a | Grin1 | Gm26546 | Dqx1 | Insig1 | Clk1 |
| Pdzk1ip1 | 9330188P03I | Adam33 | Gm26786 | Taz | Trim39 | Pfkfb2 |
| Alpl | Serpinf1 | Mfsd2a | Gm26890 | Slc9a5 | Col27a1 | Atp1b1 |
| Ing4 | Hnrnph1 | Tekt2 | Gm16754 | Dbndd2 | Ankrd34a | Rrbp1 |
| Fgf21 | Fnbp4 | Morn1 | Gm28043 | Tnfsfm13 | Sox12 | Fam210b |
| L1cam | Glis2 | Ccnl2 | Gm28119 | Pfkl | Trpm3 | Mecom |
| Adgrg1 | Ahr | Mapkapk5 | Gm28730 | Lpin1 | Zfp608 | Olfml3 |
| Amt | Clk4 | Tbx6 | Gm37968 | Mrc2 | 2700081O15I | Pdgfc |
| Catsper2 | Limk2 | Gdpd3 | Gm37795 | Vash1 | Msi1 | Bnc2 |
| Adora1 | Rapgef3 | Cox6a2 | Gm37154 | Hhipl1 | Ankrd24 | Podn |
| Lrrn4 | Clcn2 | Cngb1 | Gm38319 | Fam193b | Ddx17 | Slc2a1 |
| Cfap46 | Gtpbp2 | Ppp1r9a | Gm37309 | Sla | Zfp607b | Mthfr |
| 4732471J01F | Atat1 | Inha | Gm37274 | Chkb | Rnf213 | Clcn6 |
| Izumo4 | Arhgef9 | Ssc5d | Gm38220 | Thpo | Mn1 | Gm996 |
| Fam57b | Tmeff2 | Rsrp1 | Gm38020 | Lmbr1l | Kcnip3 | Col1a2 |
| Snora2b | Rassf8 | Pkhd1l1 | Gm38077 | Amhr2 | Tnfsf13os | Adamts9 |
| Celsr2 | Fgfr2 | Shank1 | Gm43858 | Satb1 | Gm38394 | Pianp |
| 4933439C10I | Ankrd10 | Prelp | Gm43511 | Map4k2 | Ptprv | Rhpn2 |
| Wt1os | 6430548M08 | Fam129c | Gm43413 | Pcyt2 | Xndc1 | Abcd1 |
| 1700020D05I | Hyou1 | Exoc3l | Gm43445 | Cfh | Pcdhga5 | Col4a2 |
| Gm49396 | Gmip | Casc1 | Gm43071 | Ttc14 | Gmpr | Plpp5 |
| Muc16 | Nod1 | Sh2d5 | Gm42603 | Epb41l4b | Col1a1 | Slc27a1 |
| Gm20219 | Gcnt1 | Smtnl2 | Gm36535 | Ak4 | Grik5 | Fam46a |
| AC122335.1 | Spns2 | Rimkla | Gm43080 | Mmp17 | Crtc1 | Man2c1 |

Table S1c: List of genes affected by Bu specifically in siCtrl-transfected cells.

| | | | | |
|---|---|---|---|---|
| Adamts7 | Kmt5c | Gm6030 | Efcab11 | Dlk2 |
| Zic1 | Rpl3 | Ethe1 | Dcaf4 | Mlf1 |
| Nphp3 | Nr6a1 | Rpl13a | Trip13 | Fam212b |
| Mst1r | Tacc1 | Gm8199 | Fst | Fam241a |
| Abcc10 | Sptbn2 | Fam26e | Dnajc9 | Ubald2 |
| Lss | Clk2 | Perp | Diaph3 | Insl6 |
| Szt2 | Rbm47 | Rspo3 | Eif3e | Nat2 |
| Stard9 | Apcdd1 | Cox7a2l | Ly6e | Zfp472 |
| Zfp446 | Wdr90 | Mmp24 | Rfc4 | Glipr1 |
| Ogt | Ptar1 | Rps3a1 | Pros1 | Zfp729b |
| Slc25a37 | 6330403L08R | Glipr2 | Cbr3 | Ube2s |
| Sirt6 | Bnip3 | Slc19a2 | Thbs2 | Ei24 |
| Tet3 | Rab10os | Rpl37 | Pigf | Arhgap22 |
| Pcyt1b | Gm2415 | S100a13 | Rps14 | Vrk2 |
| Ror1 | Gm7694 | Rps8 | Fas | Syt11 |
| Ntng2 | Gna14 | Gm8730 | Crcp | Zfp874a |
| Aldh1b1 | Aqp3 | Rpl23 | Stk17b | Tmem19 |
| Prpf39 | S100a7a | Fndc10 | Irf6 | Nanos1 |
| Sugp2 | Gsto1 | Wnt4 | Phospho2 | Slfn2 |
| Stag3 | Lamb3 | 2610044O15 | Eif3m | 5430403G16Rik |
| Uvssa | Fgf13 | AC129328.1 | Fabp5 | Osgin1 |
| Ahdc1 | Plet1 | Slc2a9 | Exosc9 | Smim26 |
| Kmt2c | Acta2 | Slfn3 | Tlr2 | Arrdc3 |
| Setd1b | Anpep | Ms4a10 | Polr1e | Rad54b |
| Higd1a | Cd24a | Pnliprp2 | Prdx1 | Zfp429 |
| Rere | Nipal1 | Casq2 | Stmn1 | Gm14681 |
| Spsb1 | 1110038B12 | Nppb | Rcc1 | Gm20667 |
| Zfhx2 | Gdf15 | Cgref1 | Lzic | Zfp960 |
| Cables1 | Adgrf4 | Mylpf | Triap1 | Zfp850 |
| Tspyl2 | Rps20 | Ltf | Mphosph10 | |
| Cacna2d4 | Rps27l | Mslnl | Ap1s2 | |
| Bicdl1 | Eva1c | Gm11223 | Vps36 | |
| Acacb | Ecscr | Gpr85 | Mfap3l | |
| Msantd2 | Rpl39 | Slc29a4 | Tbc1d9 | |
| Zmym6 | Rps10 | Defb42 | Ccsap | |
| 4632404H12 | Col14a1 | 1700003M07 | Abhd10 | |
| Safb2 | Duoxa1 | Gm17655 | Reep4 | |
| Trim46 | Cyp2b10 | 9430037O13 | Tmem5 | |
| Upk3b | Prr7 | Gria3 | Celf5 | |
| Ccdc84 | Mettl7a3 | Def6 | Trim13 | |
| Als2cl | Alox5ap | Tbc1d8 | Calm2 | |
| Dock5 | Tubb3 | Hspb1 | Cib2 | |
| Krt14 | 4930471E19R | Tek | Rpf2 | |
| Onecut2 | Dram1 | Cct4 | Ephx1 | |
| Slc25a23 | Dglucy | Snrpb2 | Myl6b | |
| Vat1l | Nme4 | Hyal1 | Rbm48 | |
| Khnyn | Cyp2c55 | Rdm1 | Pitpnc1 | |
| Kmt2d | Spint1 | Mrpl32 | St8sia4 | |
| Hoxb3 | Rps3 | Hebp2 | Gfer | |
| Asap2 | Phlda3 | Nudcd2 | Rpl7 | |
| Plxnb1 | Ctla2a | Jpt1 | Tnfaip8l1 | |
| Usp13 | Cd109 | Dnaaf2 | Cxcr4 | |

**Table S1c-1**: List of genes specifically decreased by Bu in siCtrl-transfected cells.

Table S1c-1: List of genes specifically decreased by Bu in siCtrl-transfected cells.

| | | | | | | |
|---|---|---|---|---|---|---|
| Mgp | AC162302.2 | Atp1b2 | Krt9 | Gm43513 | Gigyf1 | Gys1 |
| Ckmt1 | Rrnad1 | Ppp1r3b | Mpp3 | Gm43328 | Dmpk | Stx1a |
| Ttc22 | Anxa9 | Prdm9 | Lgals4 | Gm43693 | Myo7a | Zmiz1 |
| Med12l | P4ha2 | Haghl | Cpa1 | Gm42820 | Kifc3 | Vav2 |
| Gm28151 | Col6a2 | Msln | 9130017K11I | Gm43029 | Jak3 | Pou6f1 |
| Rnf112 | Nid2 | Rab44 | Rnf207 | Gm43481 | Tnxb | Adgrl1 |
| Slc22a17 | 6030458C11I | Tia1 | Clasrp | Gm43747 | Sfxn5 | Lama5 |
| Usp53 | Ly6f | Emc1 | Vmn1r13 | Gm42572 | Ppargc1b | Aldoc |
| Abca9 | Paxbp1 | Il16 | Ankrd23 | Gm42732 | Ypel4 | Kdm6b |
| Fbxl7 | Krt7 | Syn3 | Selenbp1 | Gm44243 | Tspoap1 | Rmnd1 |
| Snx32 | Wfdc1 | Gm37376 | Acrbp | Gm45838 | Adrb1 | Tns3 |
| 5430430B14I | Vegfa | Papln | 4930518I15R | Gm45221 | Nat14 | Ern1 |
| Susd2 | Ikzf2 | Aldh1l2 | Pdzd7 | Gm9856 | Nfkbiz | Slc9a3r1 |
| Rhbdl3 | Ccnt2 | Mylk | Gm2420 | Gm47963 | Crb2 | Usp43 |
| Lcor | Sema6d | Fam151a | Gm9164 | Gm47113 | Zfp57 | Zfp36l1 |
| Slco5a1 | Ccnl1 | Fmnl1 | Gm5466 | Gm47483 | Ppfibp2 | Srsf5 |
| Rnft2 | Plxnb3 | Per2 | Gm15344 | Gm33869 | Arhgap33 | Abcd4 |
| Plekha7 | Car12 | Gm38391 | Gm15337 | Gm47583 | Mycn | Ptch1 |
| Fgf18 | Zfc3h1 | Srpk3 | Firre | Gm47585 | Bcl9 | Pde4d |
| Pisd-ps1 | Sv2a | Rpl10 | Gm15513 | Gm47798 | Angptl6 | Hmbox1 |
| Dennd6b | Gjb3 | Cdh23 | Chn1os3 | Gm49223 | Whrn | Wnt5a |
| Ager | Synpo | Kpna2 | BC065397 | AC133488.1 | Lrp1 | Sdf2l1 |
| Rac3 | Il17re | Cyp46a1 | Gm11266 | Col6a1 | Clcf1 | Map3k8 |
| Adcy1 | Zbtb37 | Naprt | C030037D09I | Cldn15 | Crocc | Tcf7l2 |
| Dnase1l2 | Sv2c | Pgc | Gm23547 | Hmgn2 | Mill2 | Lrrc45 |
| Mapk8ip3 | Dock8 | Pi16 | Gm16574 | Eno2 | Dennd4b | Slc16a3 |
| Nudt8 | Ggcx | Rgs11 | Gm17024 | Pdk1 | Hoxd11 | Mbd6 |
| Lrp2 | Dleu2 | Mamdc4 | Mir5125 | Runx1t1 | Cilp2 | Pdia4 |
| Pnisr | Zc3h11a | Grin1 | Gm26546 | Dqx1 | Insig1 | Clk1 |
| Pdzk1ip1 | 9330188P03I | Adam33 | Gm26786 | Taz | Trim39 | Pfkfb2 |
| Alpl | Serpinf1 | Mfsd2a | Gm26890 | Slc9a5 | Col27a1 | Atp1b1 |
| Ing4 | Hnrnph1 | Tekt2 | Gm16754 | Dbndd2 | Ankrd34a | Rrbp1 |
| Fgf21 | Fnbp4 | Morn1 | Gm28043 | Tnfsfm13 | Sox12 | Fam210b |
| L1cam | Glis2 | Ccnl2 | Gm28119 | Pfkl | Trpm3 | Mecom |
| Adgrg1 | Ahr | Mapkapk5 | Gm28730 | Lpin1 | Zfp608 | Olfml3 |
| Amt | Clk4 | Tbx6 | Gm37968 | Mrc2 | 2700081O15I | Pdgfc |
| Catsper2 | Limk2 | Gdpd3 | Gm37795 | Vash1 | Msi1 | Bnc2 |
| Adora1 | Rapgef3 | Cox6a2 | Gm37154 | Hhipl1 | Ankrd24 | Podn |
| Lrrn4 | Clcn2 | Cngb1 | Gm38319 | Fam193b | Ddx17 | Slc2a1 |
| Cfap46 | Gtpbp2 | Ppp1r9a | Gm37309 | Sla | Zfp607b | Mthfr |
| 4732471J01F | Atat1 | Inha | Gm37274 | Chkb | Rnf213 | Clcn6 |
| Izumo4 | Arhgef9 | Ssc5d | Gm38220 | Thpo | Mn1 | Gm996 |
| Fam57b | Tmeff2 | Rsrp1 | Gm38020 | Lmbr1l | Kcnip3 | Col1a2 |
| Snora2b | Rassf8 | Pkhd1l1 | Gm38077 | Amhr2 | Tnfsf13os | Adamts9 |
| Celsr2 | Fgfr2 | Shank1 | Gm43858 | Satb1 | Gm38394 | Pianp |
| 4933439C10I | Ankrd10 | Prelp | Gm43511 | Map4k2 | Ptprv | Rhpn2 |
| Wt1os | 6430548M08 | Fam129c | Gm43413 | Pcyt2 | Xndc1 | Abcd1 |
| 1700020D05I | Hyou1 | Exoc3l | Gm43445 | Cfh | Pcdhga5 | Col4a2 |
| Gm49396 | Gmip | Casc1 | Gm43071 | Ttc14 | Gmpr | Plpp5 |
| Muc16 | Nod1 | Sh2d5 | Gm42603 | Epb41l4b | Col1a1 | Slc27a1 |
| Gm20219 | Gcnt1 | Smtnl2 | Gm36535 | Ak4 | Grik5 | Fam46a |
| AC122335.1 | Spns2 | Rimkla | Gm43080 | Mmp17 | Crtc1 | Man2c1 |

Table S1c-1: List of genes specifically decreased by Bu in siCtrl-transfected cells.

| | |
|---|---|
| Adamts7 | Kmt5c |
| Zic1 | Rpl3 |
| Nphp3 | Nr6a1 |
| Mst1r | Tacc1 |
| Abcc10 | Sptbn2 |
| Lss | Clk2 |
| Szt2 | Rbm47 |
| Stard9 | Apcdd1 |
| Zfp446 | Wdr90 |
| Ogt | Ptar1 |
| Slc25a37 | 6330403L08Rik |
| Sirt6 | Bnip3 |
| Tet3 | Rab10os |
| Pcyt1b | Gm2415 |
| Ror1 | Gm7694 |
| Ntng2 | |
| Aldh1b1 | |
| Prpf39 | |
| Sugp2 | |
| Stag3 | |
| Uvssa | |
| Ahdc1 | |
| Kmt2c | |
| Setd1b | |
| Higd1a | |
| Rere | |
| Spsb1 | |
| Zfhx2 | |
| Cables1 | |
| Tspyl2 | |
| Cacna2d4 | |
| Bicdl1 | |
| Acacb | |
| Msantd2 | |
| Zmym6 | |
| 4632404H12Rik | |
| Safb2 | |
| Trim46 | |
| Upk3b | |
| Ccdc84 | |
| Als2cl | |
| Dock5 | |
| Krt14 | |
| Onecut2 | |
| Slc25a23 | |
| Vat1l | |
| Khnyn | |
| Kmt2d | |
| Hoxb3 | |
| Asap2 | |
| Plxnb1 | |
| Usp13 | |

**Table S1c-2**: List of genes specifically increased by Bu in siCtrl-transfected cells.

Table S1c-2: List of genes specifically increased by Bu in siCtrl-transfected cells.

| | | | |
|---|---|---|---|
| Gna14 | Gm8730 | Crcp | Zfp874a |
| Aqp3 | Rpl23 | Stk17b | Tmem19 |
| S100a7a | Fndc10 | Irf6 | Nanos1 |
| Gsto1 | Wnt4 | Phospho2 | Slfn2 |
| Lamb3 | 2610044O15I | Eif3m | 5430403G16Rik |
| Fgf13 | AC129328.1 | Fabp5 | Osgin1 |
| Plet1 | Slc2a9 | Exosc9 | Smim26 |
| Acta2 | Slfn3 | Tlr2 | Arrdc3 |
| Anpep | Ms4a10 | Polr1e | Rad54b |
| Cd24a | Pnliprp2 | Prdx1 | Zfp429 |
| Nipal1 | Casq2 | Stmn1 | Gm14681 |
| 1110038B12I | Nppb | Rcc1 | Gm20667 |
| Gdf15 | Cgref1 | Lzic | Zfp960 |
| Adgrf4 | Mylpf | Triap1 | Zfp850 |
| Rps20 | Ltf | Mphosph10 | |
| Rps27l | Mslnl | Ap1s2 | |
| Eva1c | Gm11223 | Vps36 | |
| Ecscr | Gpr85 | Mfap3l | |
| Rpl39 | Slc29a4 | Tbc1d9 | |
| Rps10 | Defb42 | Ccsap | |
| Col14a1 | 1700003M07 | Abhd10 | |
| Duoxa1 | Gm17655 | Reep4 | |
| Cyp2b10 | 9430037O13I | Tmem5 | |
| Prr7 | Gria3 | Celf5 | |
| Mettl7a3 | Def6 | Trim13 | |
| Alox5ap | Tbc1d8 | Calm2 | |
| Tubb3 | Hspb1 | Cib2 | |
| 4930471E19F | Tek | Rpf2 | |
| Dram1 | Cct4 | Ephx1 | |
| Dglucy | Snrpb2 | Myl6b | |
| Nme4 | Hyal1 | Rbm48 | |
| Cyp2c55 | Rdm1 | Pitpnc1 | |
| Spint1 | Mrpl32 | St8sia4 | |
| Rps3 | Hebp2 | Gfer | |
| Phlda3 | Nudcd2 | Rpl7 | |
| Ctla2a | Jpt1 | Tnfaip8l1 | |
| Cd109 | Dnaaf2 | Cxcr4 | |
| Gm6030 | Efcab11 | Dlk2 | |
| Ethe1 | Dcaf4 | Mlf1 | |
| Rpl13a | Trip13 | Fam212b | |
| Gm8199 | Fst | Fam241a | |
| Fam26e | Dnajc9 | Ubald2 | |
| Perp | Diaph3 | Insl6 | |
| Rspo3 | Eif3e | Nat2 | |
| Cox7a2l | Ly6e | Zfp472 | |
| Mmp24 | Rfc4 | Glipr1 | |
| Rps3a1 | Pros1 | Zfp729b | |
| Glipr2 | Cbr3 | Ube2s | |
| Slc19a2 | Thbs2 | Ei24 | |
| Rpl37 | Pigf | Arhgap22 | |
| S100a13 | Rps14 | Vrk2 | |
| Rps8 | Fas | Syt11 | |

**Table S2**: List of genes specifically increased by Bu in siCtrl-transfected cells and determined as TP53 associated genes using I-Cistarget.

Table S2: List of genes specifically increased by Bu in siCtrl-transfected cells and determined as TP53 associated genes using I-Cistarget.

| | | |
|---|---|---|
| Fam212b | Mylpf | Phospho2 |
| Rps27l | Rps8 | Eif3e |
| Cd109 | Tmem19 | |
| Fst | Tbc1d9 | |
| Fas | Nipal1 | |
| Prr7 | Vps36 | |
| 1700003M07 | Gsto1 | |
| Def6 | Rps10 | |
| Casq2 | Cd24a | |
| Phlda3 | Slfn2 | |
| Pnliprp2 | Gria3 | |
| Mfap3l | Vrk2 | |
| Reep4 | Spint1 | |
| Gpr85 | Rps3a1 | |
| Stk17b | Ephx1 | |
| Mmp24 | Rps14 | |
| Ap1s2 | Acta2 | |
| Fgf13 | Celf5 | |
| Gdf15 | Rpl7 | |
| Rspo3 | Insl6 | |
| Ei24 | Snrpb2 | |
| Slc2a9 | Mslnl | |
| Cxcr4 | Eif3m | |
| Perp | Abhd10 | |
| Ecscr | Tmem5 | |
| Wnt4 | Nudcd2 | |
| Ms4a10 | Cbr3 | |
| Dram1 | Trim13 | |
| Lamb3 | St8sia4 | |
| Duoxa1 | Gna14 | |
| Hyal1 | Rfc4 | |
| Dlk2 | Mlf1 | |
| Plet1 | Dnajc9 | |
| Calm2 | Rdm1 | |
| Eva1c | Tlr2 | |
| Col14a1 | Adgrf4 | |
| Glipr2 | Hspb1 | |
| Arhgap22 | Rcc1 | |
| Anpep | Fam26e | |
| Slc19a2 | Ly6e | |
| Dnaaf2 | Tnfaip8l1 | |
| Aqp3 | Fabp5 | |
| S100a7a | Thbs2 | |
| Tek | Crcp | |
| Tbc1d8 | Glipr1 | |
| Osgin1 | Pros1 | |
| Pitpnc1 | Cgref1 | |
| Ubald2 | Prdx1 | |
| Ltf | Stmn1 | |
| Irf6 | Cyp2b10 | |
| Dcaf4 | Rpl39 | |
| Tubb3 | Rbm48 | |

**Table S3**: List of genes specifically decreased by Bu in siCtrl-transfected cells and determined as GLIS2 associated genes using I-Cistarget.

Table S3: List of genes specifically decreased by Bu in siCtrl-transfected cells and determined as GLIS2 associated genes using I-Cistarget.

| | | | | |
|---|---|---|---|---|
| 4732471J01R | Crb2 | Krt14 | Ppfibp2 | Trim46 |
| 4933439C10I | Crocc | Krt9 | Ppp1r3b | Trpm3 |
| 6430548M08 | Crtc1 | L1cam | Ppp1r9a | Ttc14 |
| 9330188P03I | Cyp46a1 | Lama5 | Prelp | Ttc22 |
| Abcd4 | Dbndd2 | Lcor | Ptar1 | Upk3b |
| Acacb | Dennd4b | Limk2 | Ptch1 | Usp13 |
| Adam33 | Dleu2 | Lmbr1l | Ptprv | Usp43 |
| Adamts7 | Dmpk | Lrp1 | Rab44 | Vash1 |
| Adamts9 | Dock5 | Lrp2 | Rapgef3 | Vav2 |
| Adcy1 | Dock8 | Lss | Rbm47 | Vegfa |
| Adgrg1 | Dqx1 | Man2c1 | Rere | Whrn |
| Adgrl1 | Eno2 | Mbd6 | Rhbdl3 | Wnt5a |
| Adora1 | Ern1 | Mecom | Rnf207 | Wt1os |
| Adrb1 | Exoc3l | Med12l | Ror1 | Ypel4 |
| Ager | Fam129c | Mfsd2a | Rrbp1 | Zbtb37 |
| Ahdc1 | Fam193b | Mgp | Runx1t1 | Zfc3h1 |
| Ahr | Fam210b | Mmp17 | Satb1 | Zfhx2 |
| Aldh1l2 | Fam57b | Mn1 | Sdf2l1 | Zfp36l1 |
| Aldoc | Fbxl7 | Morn1 | Selenbp1 | Zfp608 |
| Alpl | Fgf18 | Mpp3 | Sema6d | Zic1 |
| Als2cl | Fgfr2 | Mrc2 | Serpinf1 | Zmiz1 |
| Amhr2 | Firre | Msi1 | Setd1b | |
| Ankrd34a | Fmnl1 | Mst1r | Sfxn5 | |
| Anxa9 | Gcnt1 | Mycn | Sh2d5 | |
| Arhgap33 | Gdpd3 | Mylk | Sla | |
| Arhgef9 | Gigyf1 | Myo7a | Slc16a3 | |
| Atp1b1 | Gjb3 | Nat14 | Slc22a17 | |
| Atp1b2 | Glis2 | Nfkbiz | Slc25a23 | |
| BC065397 | Gm20219 | Nid2 | Slc25a37 | |
| Bcl9 | Gm7694 | Nod1 | Slc2a1 | |
| Bnc2 | Gmip | Nphp3 | Slc9a3r1 | |
| Bnip3 | Gmpr | Nr6a1 | Slc9a5 | |
| C030037D09I | Grik5 | Ntng2 | Slco5a1 | |
| Cables1 | Grin1 | Ogt | Smtnl2 | |
| Car12 | Gys1 | Olfml3 | Spns2 | |
| Ccnt2 | Hhipl1 | Onecut2 | Spsb1 | |
| Cdh23 | Higd1a | Papln | Sptbn2 | |
| Celsr2 | Hmgn2 | Pcyt1b | Srsf5 | |
| Chn1os3 | Hnrnph1 | Pde4d | Ssc5d | |
| Cilp2 | Hoxb3 | Pdgfc | Stx1a | |
| Ckmt1 | Hoxd11 | Pdzd7 | Sv2a | |
| Clcf1 Clcf1-po | Hyou1 | Pdzk1ip1 | Sv2c | |
| Clcn2 | Ikzf2 | Per2 | Synpo | |
| Cldn15 | Il16 | Pianp | Szt2 | |
| Clk1 | Ing4 | Pkhd1l1 | Tacc1 | |
| Clk4 | Insig1 | Plekha7 | Tbx6 | |
| Col1a1 | Jak3 | Plxnb1 | Tcf7l2 | |
| Col1a2 | Kcnip3 | Plxnb3 | Tekt2 | |
| Col27a1 | Kdm6b | Pnisr | Thpo | |
| Col4a2 | Khnyn | Podn | Tia1 | |
| Col6a1 | Kifc3 | Pou6f1 | Tns3 | |
| Cpa1 | Kmt2d | Ppargc1b | Tnxb | |

**Table S4**: List of antibodies used in this study for immunohistochemistry.

| Antibody | Supplier | Reference | Species | Amplification system | Dilution |
|---|---|---|---|---|---|
| Acetylated H4 | Millipore | 06-946 | Rabbit | No | 1/1000 |
| G9A | Cell Signaling | C6H3 | Rabbit | Yes | 1/50 |
| PLZF | Santa Cruz | SC-22839 | Rabbit | No | 1/200 |
| PCNA | Santa Cruz | SC-56 | Mouse | No | 1/500 |
| GFP | Invitrogen | A11122 | Rabbit | NO | 1/200 |

**Table S5**: List of primers used in this study.

| Gene | Fw | RV | |
|---|---|---|---|
| Actin | TCATCACTATTGGCAACGAGC | AGTTTCATGGATGCCACAGG | qPCR |
| Gfra1 | TACCACCAGCATGTCCAATGAA | GTAGCTGTGCTTGGCTGGAACT | qPCR |
| Tgr5 | GCTCCTGTCAGTCTTGGCCTAT | TTCCTCGAAGCACTCGTAGACA | qPCR |
| Perp | CACATCCAGACATCGTCGCT | AATATGGCAGCCAGTGCGAG | qPCR |
| Phlda3 | CATGTCAGCTTCTCTGTCCACT | GACCAGGTAAGGCCCAAGTC | qPCR |
| Mmp24 | AAGGGGCCTTCATCAGCAAG | CTCCACGCTCAGTTTCTGGT | qPCR |
| Glis2 | TCGATGGGCCAAGTGTAACC | GGCGGGAGAAGCTCTTGTTA | qPCR |
| Fgfr2 | GAGTCCAGCTCCTCCATGAAC | TTTGCCCAGCGTCAGCTTAT | qPCR |
| Izumo4 | TGGGTGACATTCCTGTGTCG | GACAGTCAATGCGGCTCTCT | qPCR |
| Adgrg1 | GTGCAAGGTGCACACAGTGG | CCATAGCGCAAGTGGAGTCT | qPCR |
| Wfdc1 | CTCGTGCAGAAGGAAACCCT | CAACCTCTTCAGCTCGGGAC | qPCR |
| Cyp11a1 | CTGCCTCCAGACTTCTTTCG | TTCTTGAAGGGCAGCTTGTT | qPCR |
| Fshr | GTGCTCACCAAGCTTCGAGTCAT | AAGGCCTCAGGGTTGATGTACAG | qPCR |
| Neurog3 | GCCTCATTGGAGGAATTCC | AGATGCTTGAGAGCCTCCAC | qPCR |
| Miwi2 | CAAAGGCAGACAGGACTTCG | ACTGGTACAGCTGCCAATCC | qPCR |
| Plzf | GCAGGAGCCAGCAAAGGCGA | GCAGAGACCCCAGGGAGGGG | qPCR |
| Id4 | GAGACTCACCCTGCTTTGCT | ATGCTGTCACCCTGCTTGTT | qPCR |
| Thy1 | GGCTGCTTCTGATTATTTAGTTTGTTC | ACCCACCATACGCCCTTATG | qPCR |
| Tgr5 | TGATGACACCCAACAGCACT | TAGGCTTAGGAAGAAGCAGCCA | CRISPR/Cas9 RT-PCR |

| | | | |
|---|---|---|---|
| Tgr5 | TGATGACACCCAACAGCACT | ACACTGCCATGTAGCGTTCC | CRISPR/Cas9 Genotyping |

**Table 6**: List of antibodies used in this study for western blot experiments.

| Antibody | Supplier | Reference | Species | Dilution |
|---|---|---|---|---|
| GAPDH | MC2-Sigma | G9545 | Rabbit | 1/10000 |
| P-TP53 | Cell Signaling | 9284S | Rabbit | 1/5000 |
| TP53 | Cell Signaling | 9282S | Rabbit | 1/5000 |
| GLIS2 | Novus Biologicals | NBP2-41311 | Rabit | 1/1000 |
