# Supplementary material for: Identification of a Crosstalk among TGR5, GLIS2, and TP53 Signaling Pathways in the Control of Undifferentiated Germ Cell Homeostasis and Chemoresistance
Source: Adv Sci (Weinh). 2022 Apr 18;9(17):2200626. doi: 10.1002/advs.202200626 (PMC9189661; doi:10.1002/advs.202200626)
Supplement: Supplementary file 2 — Supporting Information [file ADVS-9-2200626-s002.pdf]

ADVANCED SCIENCE
Open Access

Supporting Information

for *Adv. Sci.*, DOI 10.1002/advs.202200626

Identification of a Crosstalk among TGR5, GLIS2, and TP53 Signaling Pathways in the Control of Undifferentiated Germ Cell Homeostasis and Chemoresistance

*Laura Thirouard, Hélène Holota, Mélusine Monrose, Manon Garcia, Angélique de Haze, Christelle Damon-Soubeyrand, Yoan Renaud, Jean-Paul Saru, Alessia Perino, Kristina Schoonjans, Claude Beaudoin and David H. Volle**

Identification of a crosstalk between TGR5, GLIS2 and TP53 signaling pathways in the control of undifferentiated germ cell homeostasis and chemoresistance.

Thirouard Laura[1], Holota Hélène[1], Monrose Mélusine[1], Garcia Manon[1], de Haze Angélique[1], Damon-Soubeyrand Christelle[1], Renaud Yoan[2], Saru Jean-Paul[1], Perino Alessia[3], Schoonjans Kristina3, Beaudoin Claude[1] and Volle David H[1].*

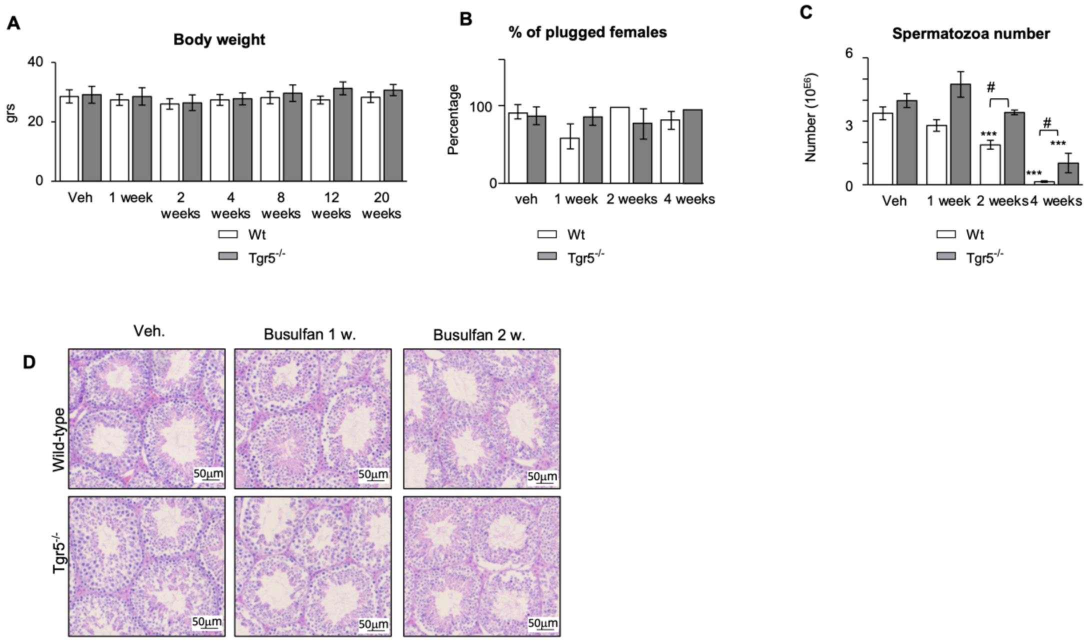

Figure S1

Figure S1.

(A) Body weight of Wt or *Tgr5*$^{-/-}$ males treated with the vehicle or busulfan for 1, 2, 4, 8, 12 or 20 weeks.

(B) Relative percentage of C57BL/6J females plugged (visualized by vaginal plug) after 10 days of reproduction with Wt or *Tgr5*$^{-/-}$ males 1, 2, 4 weeks after busulfan or vehicle treatments.

(C) Sperm count in the epididymis head of Wt or *Tgr5*$^{-/}$ males 1, 2, 4 weeks after busulfan or vehicle treatments.

(D) Representative micrographs of hematoxylin/eosin-stained testes of Wt or *Tgr5*$^{-/-}$ males treated with the vehicle or 1, 2 weeks after busulfan treatment.

In all panels, n=9-50 from 3 to 6 independent experiments. Data are expressed as the means ± SEM. ANOVA2 followed by Holm-Sidak's test for multiple comparisons. ***, $p<0.005$ *vs* respective vehicle group for each genotype. #, $p<0.05$ *between genotypes exposed to same treatments*. The horizontal square brackets underline the groups statistically compared between two conditions of different genotypes.

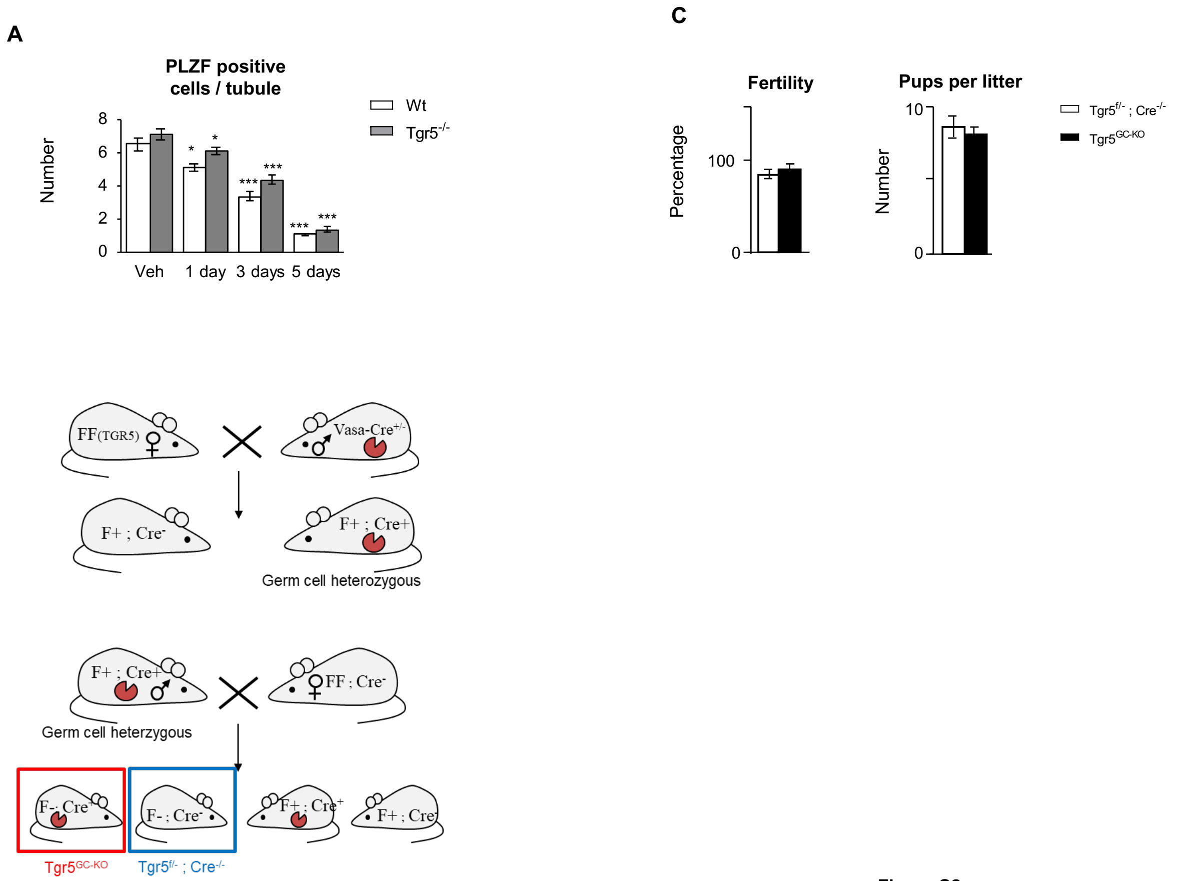

Figure S2

Figure S2.

(A) Quantification of the raw number of positive PLZF cells per seminiferous tubule in testes of *Wt* or *Tgr5*$^{-/-}$ males treated with the vehicle or busulfan (1 day, 3 days or 5 days after treatment).

(B) Schematic representation of the generation of *Tgr5* specific germ cell knockout (*Tgr5*$^{GC\text{-}KO}$).

(C) (Left) Percentage of fertile *Tgr5*$^{f/-}$*; Cre*$^{-/-}$ *and Tgr5*$^{GC\text{-}KO}$ males after vehicle treatment and breeding with C57Bl6J females for 10 days. **(Right)** Number of pups per litter obtained in breeding C57Bl6J females with *Tgr5*$^{f/-}$*; Cre*$^{-/-}$ and *Tgr5*$^{GC\text{-}KO}$ males after vehicle treatment. n=6-15 from 3 to 6 independent experiments. Data are expressed as the means ± SEM.

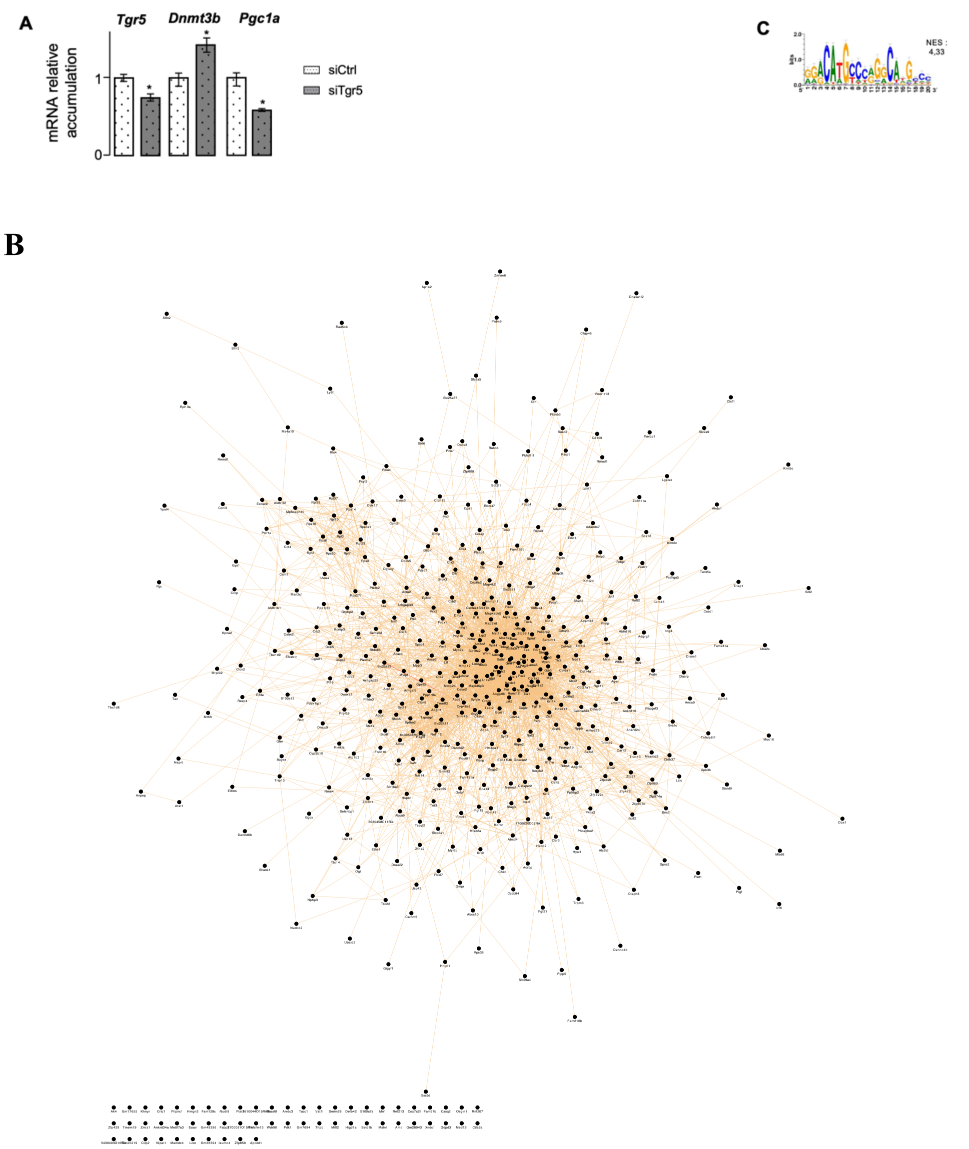

Figure S3.

(A) Relative *Tgr5, Dnmt3b and Pgc1a* mRNA accumulations normalized to β-actin in GC1spg cells transfected with siCtrl or siTgr5. 48h post transfection (at the time of treatment with Bu). n=12 from 3 to 6 independent experiments. Vehicle treated groups was arbitrary set at 1. Data are expressed as the means ± SEM. Statistical analysis: *, p<0.05 respective vehicle group for each genotype.

(B) Analysis of the interconnections of genes sorted either upwards or downwards, regulated by Bu specifically in cells transfected by siCtrl using GeneMania®.

(C) Sequences of the DNA binding site defined by CisTarget for TP53 in the genes positively regulated by Bu in siCtrl-transfected cells only.

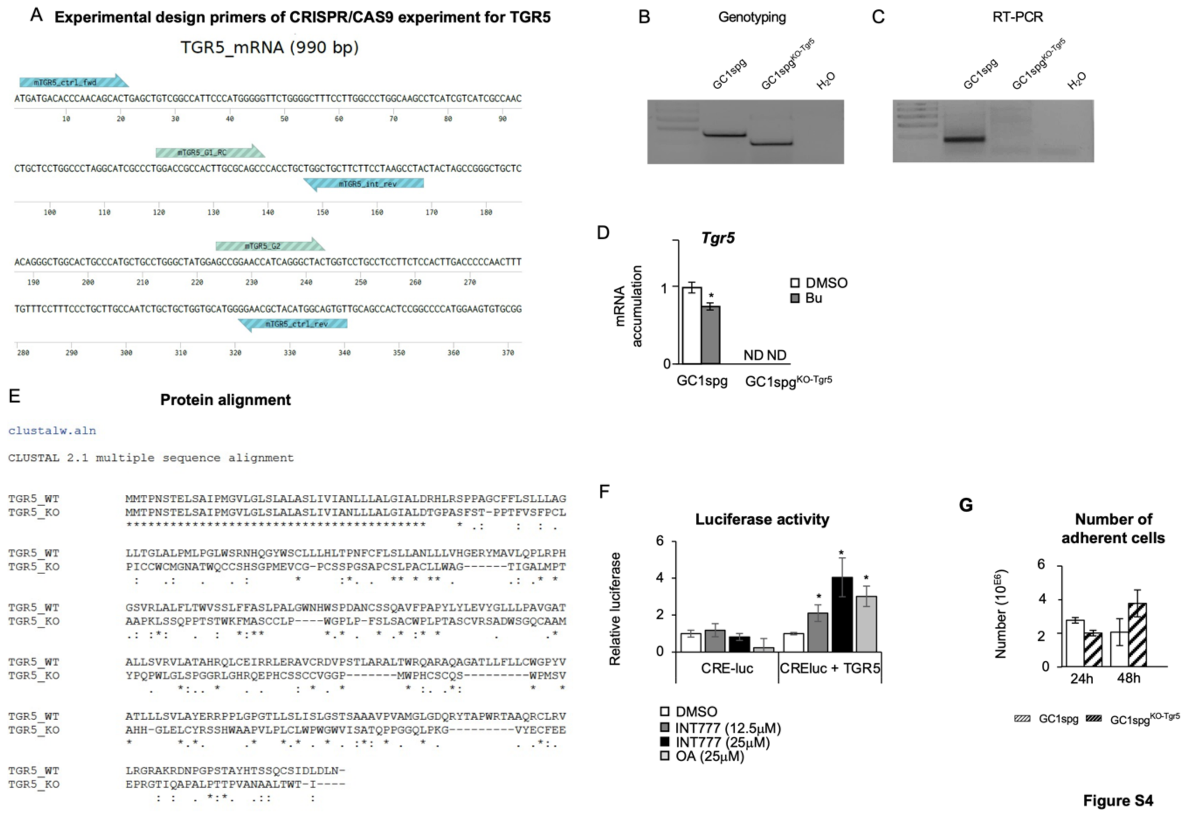

Figure S4.
(A) Schematic representation of the experimental design (primers) of Crispr/CAS9 experiment for the deletion of TGR5.

(B) Representative images of genotyping PCR.

(C) *Tgr5* mRNA accumulation observed in GC1spg and Crispr/Cas9 Tgr5 knockout GC1spg cells (GC1spg$^{KO\text{-}Tgr5}$) as observed by RT-PCR.

(D) *Tgr5* mRNA accumulation observed in GC1spg and Crispr/Cas9 Tgr5 knockout GC1spg cells (GC1spg$^{KO\text{-}Tgr5}$) as observed by RT-qPCR. Vehicle treated GC1spg cells were arbitrarily set at 1.

(E) Protein alignment between wild type TGR5 protein and the truncated protein obtained following Crispr/CAS9 protocol.

(F) Analysis of luciferase activity normalized to protein quantity in GC1spg$^{KO\text{-}Tgr5}$ transfected with cAMP-response element-luciferase (*cAMP-RE-luc)* reporter plasmid with or without co-transfection with a plasmid encoding the mouse TGR5 and treated 24 hours with vehicle, INT-777, or OA. n=12 from 3 independent experiments. Vehicle (DMSO) groups were arbitrarily set at 1. Data are expressed as the means ± SEM. ANOVA2 followed by Holm-Sidak's test for multiple comparisons. *, $p<0.05$ vehicle group for each genotype.

(**G**) Number of adherent cells in GC1spg and GC1spg$^{KO\text{-}Tgr5}$ cells exposed for 24h with vehicle for 24 or 48 hours. =12 to 15 from 3 independent experiments. Vehicle groups were arbitrarily set at 1. Data are expressed as the means ± SEM. ANOVA2 followed by Holm-Sidak's test for multiple comparisons.

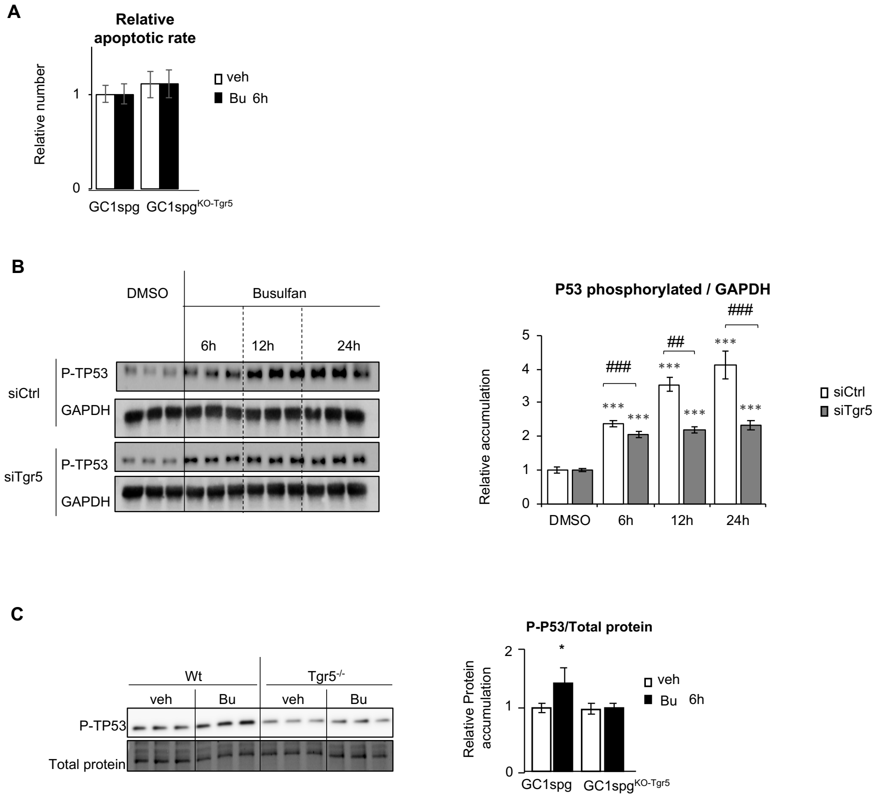

Figure S5

Figure S5.

(A) Quantification of the relative number of TUNEL positive cells of *GC1spg and* $GC1spg^{KO-Tgr5}$ cells treated with vehicle or busulfan (200µM) for 6 hours.

(B) Representative western blots of P-TP53 and GAPDH and quantification of ratios in GC1spg transfected with siCtrl or siTgr5 and treated with vehicle or Bu (200µM) for 6, 12 or 24 hours.

(C) Representative western blots of P-TP53 and quantification of ratios in GC1spg and $GC1spg^{KO-Tgr5}$ cells treated with vehicle or Bu (200µM) for 6 hours.

n=12 from 3 independent experiments. Vehicle groups were arbitrarily set at 1. Data are expressed as the means ± SEM. ANOVA2 followed by Holm-Sidak's test for multiple comparisons. *, p<0.05; ***, p<0.005 *vs* respective vehicle group for each genotype. ###, p<0.005 *between genotypes exposed to same treatments*. The horizontal square brackets underline the groups statistically compared between two conditions of different genotypes.

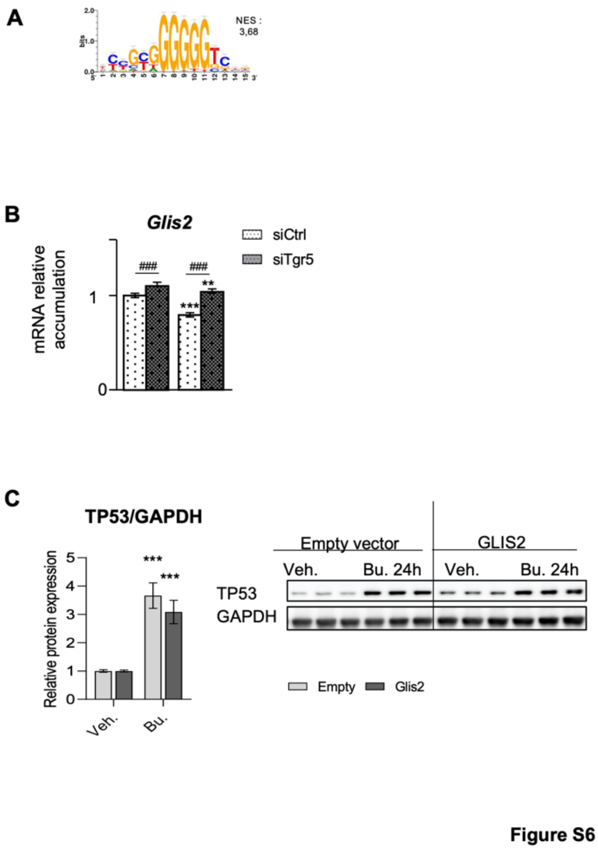

Figure S6.

(A) Sequences of the DNA binding site defined by CisTarget for GLIS2 in the genes negatively regulated by Bu in siCtrl-transfected cells specifically.

(B) *Glis2* mRNA accumulation observed in the RNAseq data from GC1spg cells transfected with siCtrl or siTgr5 and treated 24 hours with vehicle or with 200µM of Bu. Vehicle groups were arbitrarily set at 1.

(C) Representative western blots of TP53 and GAPDH and quantification of ratios in GC1spg cells transfected with empty vector or overexpression vector of GLIS2 and treated with vehicle or Bu (200µM) for 24 hours. Vehicle groups were arbitrarily set at 1.

In panels B and C, n=10. Data are expressed as the means ± SEM. ANOVA2 followed by Holm-Sidak's test for multiple comparisons. **, p<0.01; ***, p<0.005 *vs* respective vehicle group for each genotype. ###, p<0.005 *between genotypes exposed same treatments*. The horizontal square brackets underline the groups statistically compared between two conditions of different genotypes.

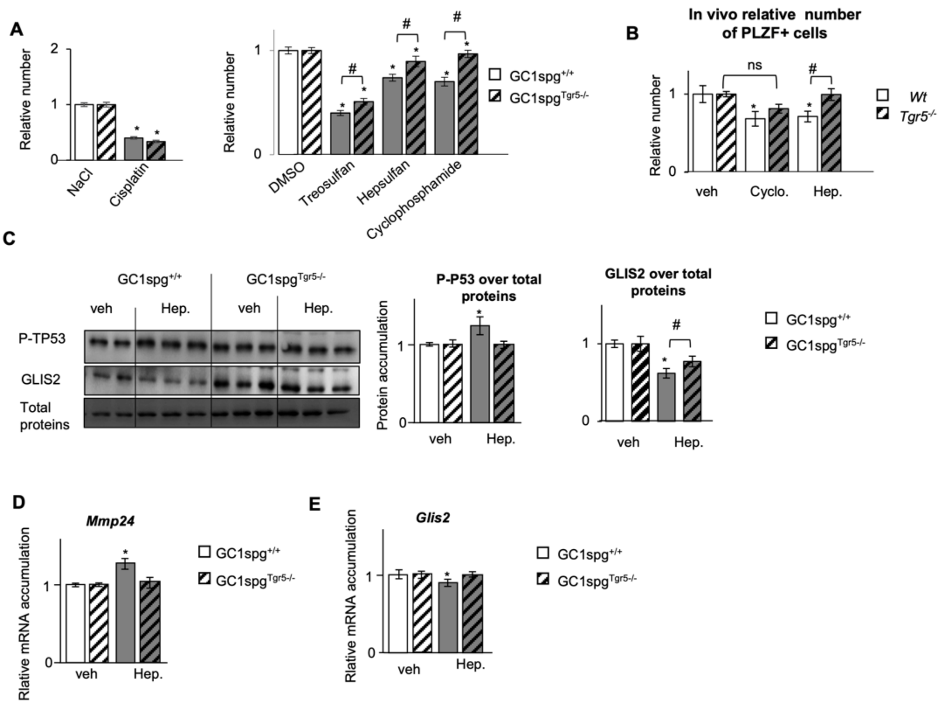

Figure S7

Figure S7.

(A) Relative number of adherent cells in GC1spg and GC1spg$^{KO-Tgr5}$ cells exposed with DMSO (1/1000), cyclophosphamide (**75µM**), hepsulfam (**3µM**), treosulfan (**10µM**), as well as with NaCl (0.9%) (1/1000) or cisplatin (**5µM**) 24 hours after treatment. n=15 from 3 independent experiments. Vehicle groups were arbitrarily set at 1. Data are expressed as the means ± SEM. ANOVA2 followed by Holm-Sidak's test for multiple comparisons. * p<0.05*vs* respective vehicle group for each genotype. #, p<0.05 *between genotypes exposed same treatments*. The horizontal square brackets underline the groups statistically compared between two conditions of different genotypes.

(B) Quantification of the relative number of PLZF positive cells per seminiferous tubule in *Wt* or *Tgr5*$^{-/-}$ testis treated with vehicle, cyclophosphamide or hepsulfam (1 week after treatment). Vehicle treated groups of each genotype were arbitrarily set at 1 for analysis. n=10. Data are expressed as the means ± SEM. ANOVA2 followed by Holm-Sidak's test for multiple comparisons. *, p<0.05*vs* respective vehicle group for each genotype. #, p<0.05 *between genotypes exposed same treatments*. The horizontal square brackets underline the groups statistically compared between two conditions of different genotypes.

(C) Representative western blots of P-TP53 and GLIS2 and quantification of ratios in GC1spg and GC1spg$^{KO\text{-}Tgr5}$ cells exposed with DMSO or hepsulfam for 24 hours. Vehicle groups were arbitrarily set at 1.

(D) *Mmp24* mRNA accumulations in GC1spg and GC1spg$^{KO\text{-}Tgr5}$ cells exposed with vehicle or hepsulfam for 24 hours. Vehicle groups were arbitrarily set at 1.

(E) Glis2 mRNA accumulation in GC1spg and GC1spg$^{KO\text{-}Tgr5}$ cells exposed with vehicle or hepsulfam for 24 hours. Vehicle groups were arbitrarily set at 1.

In panel A, C, D and E, n=15 from 3 independent experiments. Data are expressed as the means ± SEM. ANOVA2 followed by Holm-Sidak's test for multiple comparisons. *, $p<0.05$.

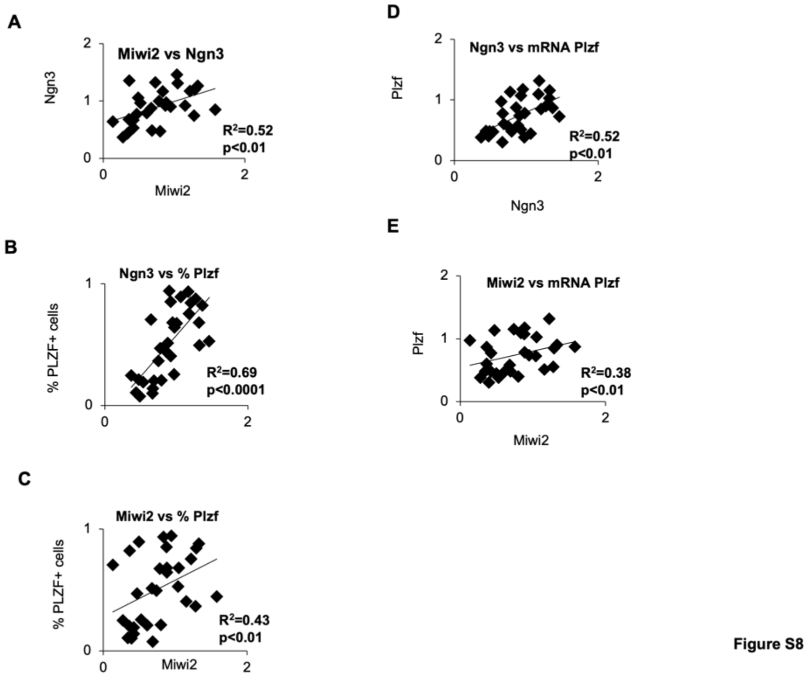

Figure S8.

(A) *Correlation analyses of the mRNA levels of Miwi2 with Ngn3 mRNA accumulations* normalized to β-actin in testis of Wt mice 5 days after the treatment with vehicle or Bu.

(B) *Correlation analyses of the mRNA levels of Ngn3 with the % of PLZF + cells* in testis of Wt mice 5 days after the treatment with vehicle or Bu.

(C) *Correlation analyses of the mRNA levels of Miwi2 with the % of PLZF + cells* in testis of Wt mice 5 days after the treatment with vehicle or Bu.

(D) *Correlation analyses of the mRNA levels of Ngn3 with Plzf mRNA accumulation*s normalized to β-actin in testis of Wt mice 5 days after the treatment with vehicle or Bu.

(E) *Correlation analyses of the mRNA levels of Miwi2 with Plzf mRNA accumulations* normalized to β-actin in testis of Wt mice 5 days after the treatment with vehicle or Bu.

In all panels n= at least 15 from 3 independent experiments. Spearman Statistical analysis: *, $p<0.05$.

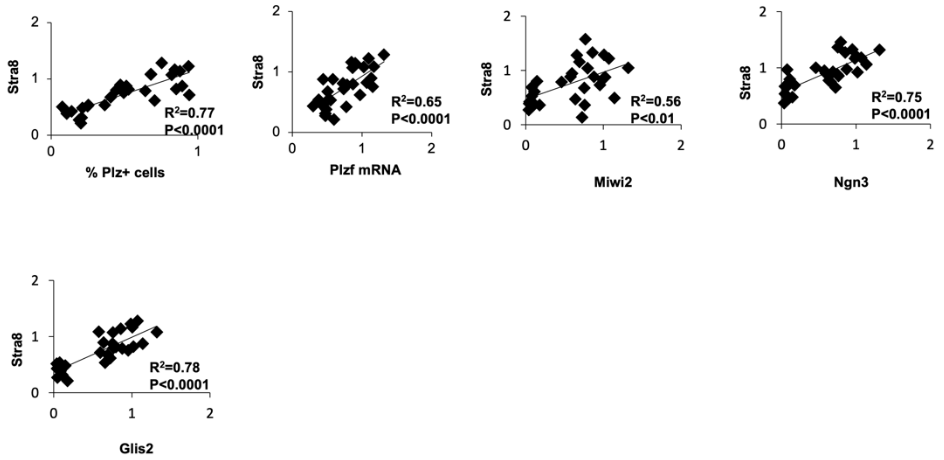

Figure S9

Figure S9.

Correlation analyses of the mRNA levels of Stra8 with the % of PLZF + cells or Plzf or Ngn3 or Glis2 or Miwi2 mRNA accumulations normalized to β-actin in testis of Wt mice 5 days after the treatment with vehicle or Bu**.**

In all panels n= at least 15 from 3 independent experiments. Spearman Statistical analysis: *, $p<0.05$. Veh: vehicle and Bu: Busulfan.

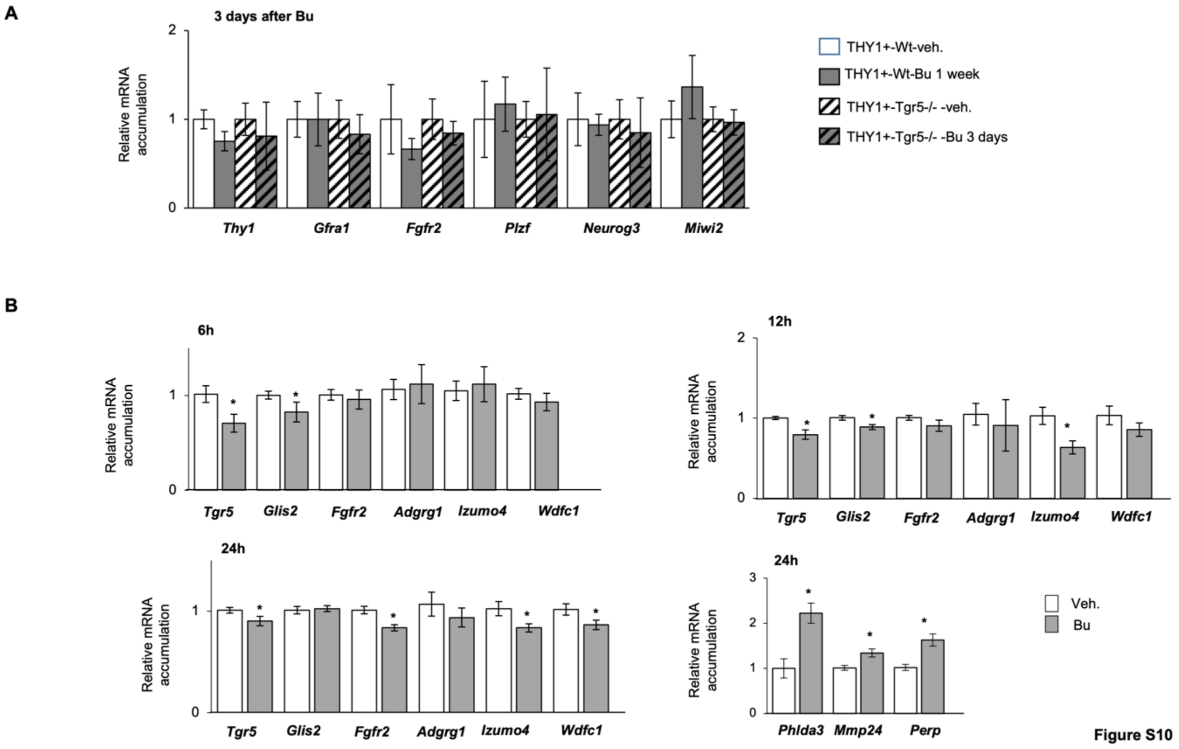

Figure S10.

(A) *Thy1, Gfra1, Glis2*, *Fgfr2*, Plzf, Ngn3 and Miwi2 mRNA accumulations normalized to β-actin in THY1+ isolated spermatogonia of adult Wt and Tgr5$^{-/-}$ mice 3 days or 1 week after the treatment with vehicle or Bu**.** In panel, n=10 to 15 from 3 independent experiments. Vehicle groups were arbitrarily set at 1.

(B*) Tgr5, Glis2*, *Fgfr2*, Izumo4, Wfdc1 mRNA accumulations normalized to β-actin in GC1spg cells transfected with siCtrl 6 h, 12 h or 24 h after the treatment with vehicle or Bu**.** *Phlda3, Mmp24 and Perp* mRNA accumulations normalized to β-actin in GC1spg cells transfected with siCtrl 24 h after the treatment with vehicle or Bu**.** siCtrl-transfected cells were set at 1.

n=15, Vehicle groups were arbitrarily set at 1. Data are expressed as the means ± SEM. Statistical analysis: *, $p<0.05$ *vs* respective vehicle group for each genotype.

Analyzed sequences

		Accession numbers	Number of potential CREB binding site in individual sequences
**Mouse Glis2**	**Gene Id 83396**	GXP_129869	9
		GXP_3071747	7
		GXP_3071748	4
		GXP_3071748	8
		GXP_3071750	11
		GXP_6737696	11
**Human GLIS2**	**Gene Id 84662**	GXP_6059980	5
		GXP_5144025	5
		GXP_9528850	14
		GXP_200804	5
		GXP_5121072	2
		GXP_200805	5
		GXP_9509639	14
	Number of potential CREB binding sites in all the sequences analyzed	**Match Total**	100

	Significance value given for common TF bnding s overall the sequences analyzed	Number of potential CREB binding sites in all the sequences analyzed	Number of common CREB sequences in all the sequences analyzed
**Matrix Family**	**p-value**	**Match Total**	**Common to the sequences**
V$CREB	0,0270873	100	13

Data on interaction TGR5 GLIS2: potential CREB binding sites

Figure S11

Figure S11

Analysis of Glis2 5’ regulatory sequences (-2000pb; +500bp) of different species using Genomatix Matinspector.

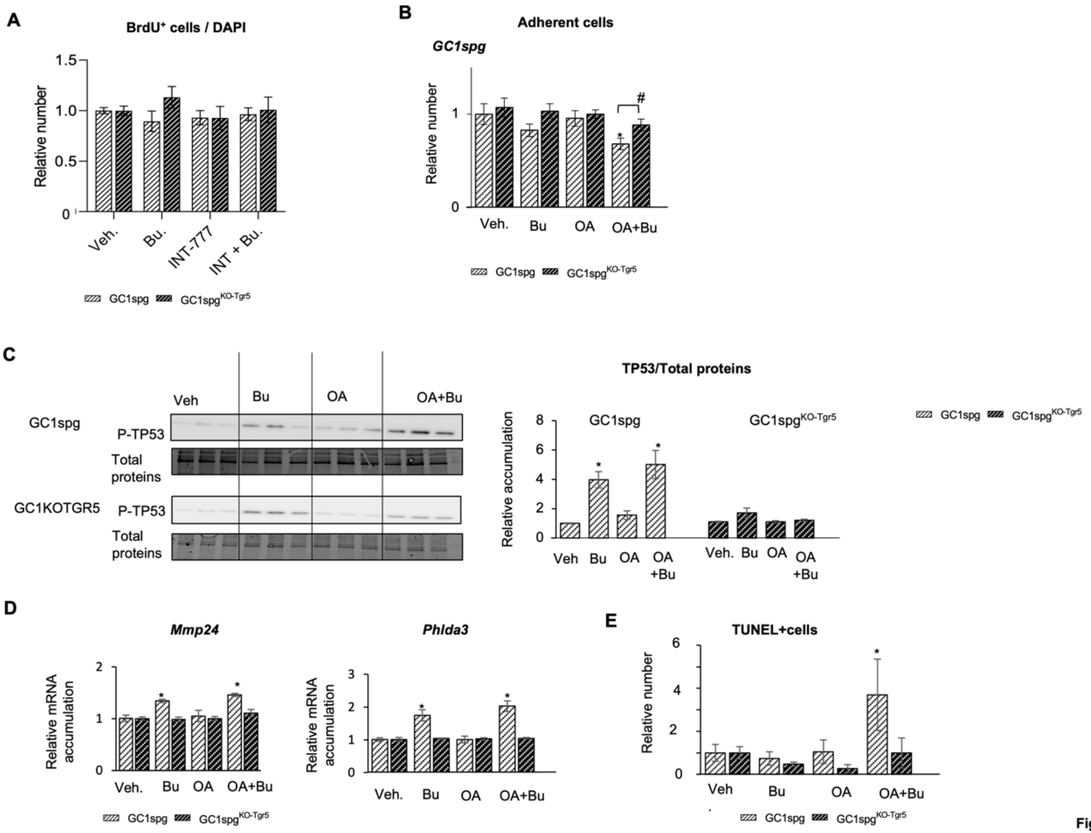

Figure S12

Figure S12.

(A) Quantification of the relative number of BrdU positive GC1spg and GC1spg$^{KO-Tgr5}$ cells pre-exposed 24 h with vehicle or INT-777 and then to vehicle or Bu (200µM) for 24 h**.** n=15 to 21 from 3 to 6 independent experiments. Veh.: vehicle; Bu: Busulfan. Vehicle treated cells were arbitrarily set at 1.

(B) Number of adherent cells in GC1spg and GC1spg$^{KO-Tgr5}$ cells pre-treated for 24 h with vehicle or OA and then for 24 hours with vehicle of Bu. Vehicle treated cells were arbitrarily set at 1.

(C) Representative western blots of P-TP53 and quantification of ratios in GC1spg and GC1spg$^{KO-Tgr5}$ cells pre-treated for 24 h with vehicle or OA and then for 24 h with vehicle of Bu. Vehicle treated cells were arbitrarily set at 1.

(D) *Mmp24* and *Phlda3* mRNA accumulations on *GC1spg and GC1spg$^{KO-Tgr5}$* pre-treated for 24 h with vehicle or OA and then for 24 h with vehicle of Bu. Vehicle treated cells were arbitrarily set at 1.

(E) Quantification of the relative number of TUNEL positive cells per seminiferous tubules in testes of *GC1spg and GC1spg$^{KO-Tgr5}$* cells pre-treated for 24 h with vehicle or OA and then for 24 h with vehicle of Bu.

In all panels, n= 12 to 15 from 3 to 5 independent experiments. Data are expressed as the means ± SEM. ANOVA2 followed by Holm-Sidak's test for multiple comparisons. * $p<0.05$; *vs* respective vehicle group for each genotype. #, $p<0.05$ *between genotypes exposed same treatments*. The horizontal square brackets underline the groups statistically compared between two conditions of different genotypes.

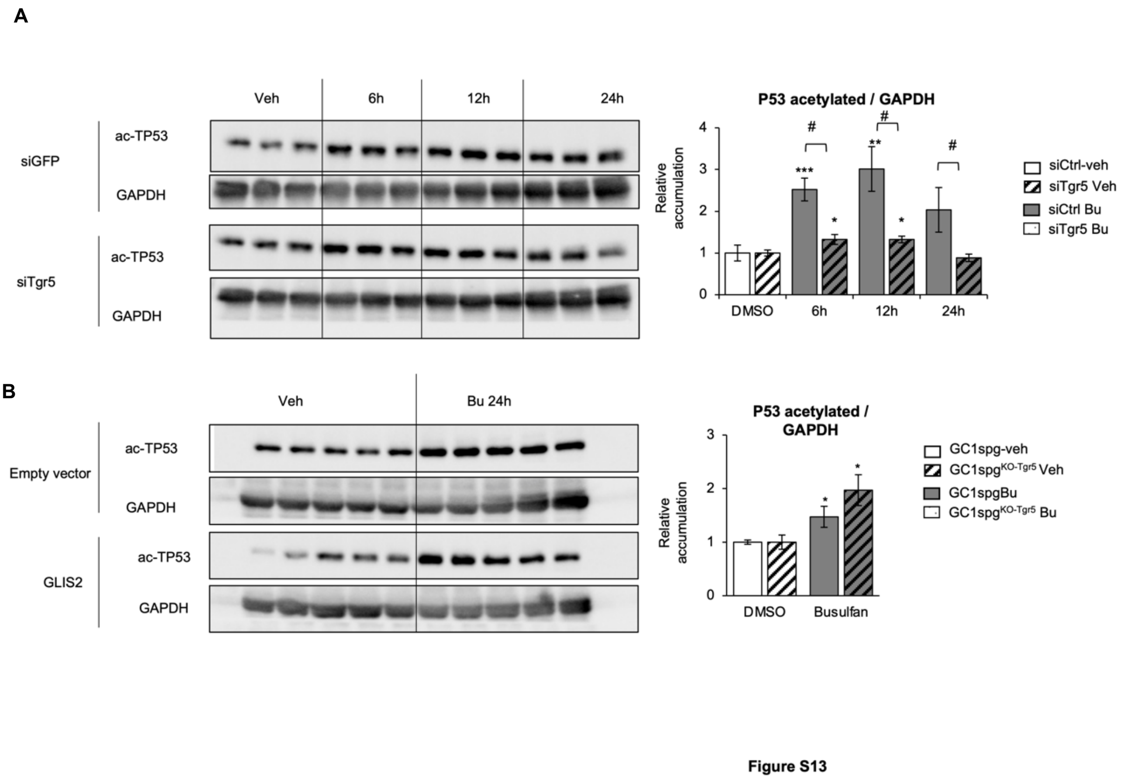

Figure S13.

(A) Representative western blots of GAPDH, and acetlylated-TP53 (as-TP53), and quantification of ratios in GC1spg cells transfected with siCtrl and siTgr5 and treated with vehicle or Bu for 6 h, 12 h or 24 h. Vehicle treated cells were arbitrarily set at 1.

(B) Representative western blots of GAPDH and acetylated-TP53 (ac-TP53), and quantification of ratios in GC1spg cells transfected with an empty vector or a vector for overexpression of GLIS2 and treated with vehicle or Bu for 24 hours. Vehicle treated cells were arbitrarily set at 1.

In all panels n=15, Data are expressed as the means ± SEM. ANOVA2 followed by Holm-Sidak's test for multiple comparisons. *, p<0.05; ** p<0.01; *** p<0.005 *vs* respective vehicle group for each genotype. #, p<0.05 *between genotypes exposed same treatments*. The horizontal square brackets underline the groups statistically compared between two conditions of different genotypes.

Table S1a: List of genes affected by Bu versus veh. in siCtrl-transfected cells.

Table S1a-1: List of genes decreased by Bu versus veh. in siCtrl-transfected cells.

Table S1a-2: List of genes increased by Bu versus veh. in siCtrl-transfected cells.

Table S1b: List of genes affected by Bu versus veh. in siTgr5-transfected cells.

Table S1b-1: List of genes decreased by Bu versus veh. in siTgr5-transfected cells.

Table S1b-2: List of genes increased by Bu versus veh. in siTgr5-transfected cells..

Table S1c: List of genes affected by Bu specifically in siCtrl-transfected cells.

Table S1c-1: List of genes specifically decreased by Bu in siCtrl-transfected cells.

Table S1c-2: List of genes specifically increased by Bu in siCtrl-transfected cells.

Table S2: List of genes specifically increased by Bu in siCtrl-transfected cells and determined as TP53 associated genes using I-Cistarget.

Table S3: List of genes specifically decreased by Bu in siCtrl-transfected cells and determined as GLIS2 associated genes using I-Cistarget.

Table S4: List of antibodies used in this study for immunohistochemistry.

Table S5: List of primers used in this study.

Table S6: List of antibodies used in this study for western blot experiments.
